# Evidence Mapping and Quality Analysis of Systematic Reviews on Various Aspects Related to Cleft Lip and Palate

**DOI:** 10.3390/jcm12186002

**Published:** 2023-09-16

**Authors:** Sukeshana Srivastav, Nitesh Tewari, Gregory S. Antonarakis, Ritu Duggal, Seba Saji, Amol Kumar Lokade, Rahul Yadav

**Affiliations:** 1 Section of Orthodontics, Department of Dentistry and Oral Health, Aarhus University, 8000 Aarhus, Denmark; 2 Division of Paediatric and Preventive Dentistry, Centre for Dental Education and Research, All India Institute of Medical Sciences, New Delhi 110029, India; 3 Division of Orthodontics, University Clinics of Dental Medicine, University of Geneva, 1205 Geneva, Switzerland; 4 Division of Orthodontics and Dentofacial Deformities, Centre for Dental Education and Research, All India Institute of Medical Sciences, New Delhi 110029, India; 5 Division of Oral and Maxillofacial Surgery, Centre for Dental Education and Research, All India Institute of Medical Sciences, New Delhi 110029, India

**Keywords:** cleft lip and palate, evidence-based medicine, systematic reviews, evidence mapping, risk of bias

## Abstract

Background: Management of cleft lip and palate is interdisciplinary. An evidence-mapping approach was envisaged to highlight the existing gaps in this field, using only the highest level of evidence. Objectives: To conduct evidence mapping and quality analysis of systematic reviews and meta-analyses related to any aspect of cleft lip and palate. Search Methods: The cleft lip and palate field was divided into 9 domains and 50 subdomains and a method of categorization of systematic reviews was established. A comprehensive search strategy was carried out in seven databases along with the search of gray literature and references of included articles. Selection criteria: Systematic reviews related to any aspect of cleft lip and palate, conducted by a minimum of two reviewers, with a comprehensive search strategy and adequate quality analysis were included. Data collection and analysis: A self-designed, pre-piloted data-extraction sheet was used to collect information that was analyzed through an expert group discussion. Quality analysis was performed using ROBIS-I, AMSTAR 2, and the PRISMA checklist. Results: A total of 144 systematic reviews published between 2008 and 2022 were included. The largest number of these could be categorized in the therapeutic domain (n = 58). A total of 27% of the studies were categorized as inconclusive, 40% as partially conclusive, and 33% as conclusive. As per ROBIS-I, 77% of reviews had high risk of bias while 58% were graded as critically low in quality as per AMSTAR 2. The majority of systematic reviews showed low reporting errors. Conclusions: The majority of systematic reviews related to cleft lip and palate relate to therapeutic and prognostic domains and show high risk of bias and critically low quality regardless of the source journal. The results of this paper might serve as a starting point encouraging authors to carry out high-quality research where evidence is lacking. Registration: A multidisciplinary expert-group formulated an a priori protocol, registered in Open Science Framework (DOI 10.17605/OSF.IO/NQDV2).

## 1. Introduction

Cleft lip and palate (CLP) is the most common congenital craniofacial anomaly, which affects roughly 1 in 700 live births globally [1,2]. It has diverse morphologic manifestations affecting the structures of the naso-maxillary complex to varying degrees of severity [1]. Its etiology is multifactorial and the association of various genetic factors has been found, for example, the involvement of genes such as MSX1, PAX9, TGF-B, and IRF-6. [2] Nonetheless, a lack of complete concordance in monozygotic twin explains the crucial role of environmental factors [2]. The management of these individuals requires interdisciplinary collaboration involving the expertise of numerous specialists such as plastic and pediatric surgeons, orthodontists, pediatric dentists, otorhinolaryngologists, speech therapists, audiologists, pediatricians, oral and maxillofacial surgeons, and geneticists. As per the degree of involvement, functional limitations prompt families of affected patients to seek care from one of the concerned specialists or ideally from an interdisciplinary team [1].

Any clinical question requires a thorough evaluation of the existing evidence in order to reach a meaningful recommendation. This was the reason for the advent, diversification, and wider acceptance of evidence-based medicine (EBM) [3]. Systematic reviews (SRs) and meta-analyses (MAs) have been placed at the highest level in the evidence pyramid and are also an indicator for the quality of primary research in any field [4] if strictly conducted based on recommended guidelines. However, the last few years have seen an escalation in the number of SRs and MAs and several researchers have raised concerns about their quality and conclusiveness [5]. Evidence mapping (EM) of SRs is an unbiased approach that follows a process of collecting and analyzing scientific evidence, systematically targeting the attainment of useful decision-making information. It helps in identifying, organizing, and summarizing the available scientific evidence on a broad topic [6]. In 2007, the protocol for EM was formulated as a part of the Global Evidence Mapping (GEM) initiative in order to map the existing literature on traumatic brain and spinal cord injuries [6]. Since then, EM has become an established method used to identify gaps in the literature and provide valuable suggestions based on needful priorities [7].

In oral health science research, EM with an additional component of quality analysis has been carried out in the fields of dental traumatology and pediatric dentistry [8,9]. Since CLP is a complex, specialized, and interdisciplinary field, performing the mapping of its evidence is difficult yet essential. It was envisaged that an EM approach would be able to identify the less-explored areas and fill the existing gaps related to CLP and develop recommendations for future research. Therefore, the aim of the present study was to conduct an EM and quality analysis of SRs and MAs existing in the literature related to any aspect of CLP.

## 2. Materials and Methods

An expert group comprising orthodontists, pediatric dentists, oral and maxillofacial surgeons, public health dentists, and library science experts was created before commencing the study (Supplementary File S1) [6]. The involved persons had prior training and experience in conducting SRs and MAs. Additional consultations with allied specialists such as audiologists, pediatric surgeons, neonatologists, geneticists, and plastic surgeons were sought at several stage of the planning and execution of the review. An a priori protocol based upon the guidelines of GEM (Supplementary Material S1) [6] and the Preferred Reporting Items for SRs and MAs (PRISMA) [10] was prepared and registered in the Open Science Framework (DOI 10.17605/OSF.IO/NQDV2). 

### 2.1. Establishment of Domains and Subdomains

During the first mapping workshop conducted by a professional facilitator, the nominal group technique was used and all the reviewers were asked to list down the possible domains, related to CLP, through anonymous generation of ideas. Various possible aspects such as growth and development, clinical questions, diagnosis, and contextual social and demographic aspects were covered. Subsequently, domains from the list were scored by two reviewers, based upon importance, novelty, and clarity (1—disagree, 2—agree, 3—agree with revision). They further ranked them as high or low priority. Two of the reviewers consulted various textbooks on CLP [11,12,13,14,15,16,17] and chapters in several reference books to verify the list of domains and subdomains, and additional amendments were suggested to the expert group.

Ten experts on CLP who were unaware of the objectives of the paper were contacted to validate the established domains and subdomains. These exercises resulted in the final establishment of 9 domains and 50 subdomains (Table 1). The field of CLP could be divided into the following domains: (i) epidemiologic, (ii) diagnostic, (iii) therapeutic, (iv) prognostic, (v) psychosocial aspects, perceptions and quality of life, (vi) preventive, (vii) recent advances, (viii) research methods, and (ix) others. The subdomains were consecutively categorized within each domain and a code was given to each (Table 1).

### 2.2. Categorization of an Article into Domains and Subdomains

The categorization of a selected article into the domains and subdomains was based on a previously published article in dental traumatology [8]. It was envisaged that the criteria for categorization must primarily be as per the participants (P), intervention or exposure (I/E), comparator (C), and outcome/s (O) (PE/ICO) of the research question. A preliminary categorization exercise based on this original framework [6] was performed by two reviewers and studies were assorted into domains and subdomains (Table 1). An inter-examiner variability was observed in this method as several studies appeared to simultaneously qualify in multiple domains and subdomains. This was attributed to the subjectivity in the importance of the components of PE/ICO among the reviewers.

**Figure 1:**
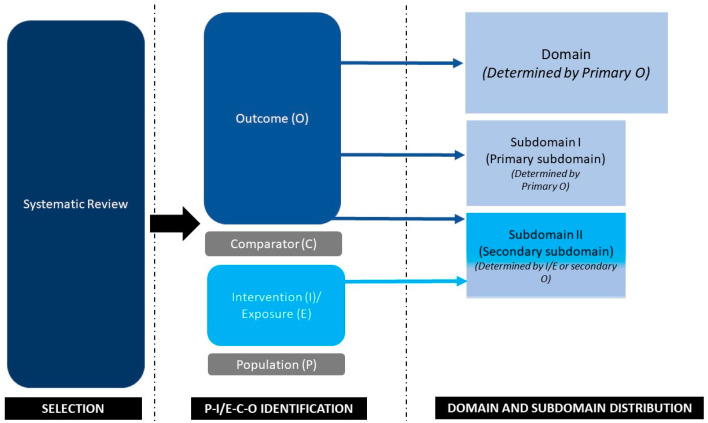
Basis of categorization of domains and subdomains.

In order to establish uniformity in categorization, the expert group recommended that the primary outcome should be considered as the basis for selecting the domain. Within each domain, the studies were further categorized into two subdomains: subdomain I (primary subdomain), which would always be a sub-classification of the respective domain (primary outcome), and subdomain II (secondary subdomain), which was based on the intervention or exposure (secondary outcome) and could be part of any of the nine domains (Figure 1; Table 1). The agreement in the new categorization method was found to be good (Cohen’s Kappa value—0.92), in a sample of 10 studies. It was proposed that any disagreement in this exercise be resolved by expert group discussion.

### 2.3. Search Strategy

PubMed, EMBASE, Lilacs, Web of Science, Cochrane reviews, Scopus, and Joanna Briggs Institute of evidence-based medicine databases were searched on 18 October 2021 without any restriction of language or year of publication. The search strategy utilizing the text words and MeSH terms based on the PE/ICO of the research question was formulated. Boolean tools “AND” and “OR” with “systematic review”, “review”, and “systematic review and meta-analysis” were used with keywords in various combinations. The strategy was suitably modified for different databases (Supplementary Materials S2a and S2b). Two authors (S.S. and Se.S.) performed the literature search independently according to this pre-defined strategy. EndNote reference management software (EndNote X 8.2 for Windows, Clarivate Analytics, Philadelphia, PA, USA) was used for removing duplicates. The same authors scrutinized the titles and abstracts as per the inclusion and exclusion criteria (Supplementary Material S2a) based on a previous EM study [8]. 

The full texts of selected articles were downloaded and screened in the second stage. There was a high level of agreement between the two reviewers in both stages of the scrutiny (Cohen’s Kappa 0.88–0.93). Any disagreement was resolved by consulting a third reviewer (N.T.). Google scholar, opengrey.eu, and worldcat.org were searched for gray literature. The references of the selected articles and hand searching of the specialty journals of CLP, orthodontics, and oral and maxillofacial or plastic surgery were also performed to identify all the possible sources of SRs and MAs related to patients with CLP. The search was updated on 18 February 2022. The registries of PROSPERO, Joanna Briggs Institute, Cochrane, and Open Science Framework were also searched on the same date to identify the listed ongoing SR protocols. The titles of these ongoing studies were also subjected to search in various databases to rule out if they were already published.

### 2.4. Data Extraction

A data extraction sheet was self-designed and pre-piloted on 10 selected articles. Two reviewers (S.S. and A.K.L.) recorded the following outcomes in a Microsoft (MS) Excel spreadsheet: (a) domain and code; (b) subdomain I and code; (c) subdomain II and code; (d) primary author; (e) year of publication; (f) journal of publication; (g) number of reviewers; (h) aims and objectives in PI/ECO; (i) protocol: registered/unregistered; (j) recommendations followed (Cochrane/PRISMA); (k) limitations of year and language used; (m) hand searching/citation searching; (n) gray literature search; (o) number of search engines; (p) name of search engines; (q) number of studies; (r) risk of bias tool/quality assessment used; (s) meta-analysis done/not done; (t) conclusion; (u) grade of conclusion.

The conclusions of studies were graded as conclusive, partially conclusive, or inconclusive. There was considerable agreement among the reviewers in this step (Cohen’s Kappa 0.84–96). Any disagreements were resolved by consulting the senior reviewer (N.T.).

### 2.5. Quality Analysis and Risk of Bias

The risk of bias (ROB)in the included reviews was assessed using ROBIS-I [18] and quality assessment was performed using AMSTAR 2 [19] tools. The reporting errors (RE) of the SRs and MAs were assessed by using the PRISMA checklist [10]. These were performed by two reviewers (S.S. and Se.S.) with good agreement (Cohen’s Kappa for ROBIS-I: 0.81–0.88; for AMSTAR 2:0.81–0.92; and for PRISMA checklist: 0.90–0.96). Any disagreements were resolved by consulting the senior reviewer (N.T.).

### 2.6. Data Presentation

The expert group analyzed the extracted data and the results of quality analysis. Since the data were expected to be considerable, their effective presentation was planned by using tables and an “Abacus evidence mapping (EM) plot” (for ROBIS-I, AMSTAR 2, and the PRISMA checklist). The Abacus EM plot is a graphical representation with the *x*-axis depicting the domains and the *y*-axis having blocks representing the categories of ROB/quality/RE. For example, the Abacus EM plot for AMSTAR 2 has four blocks representing critically low, low, moderate, and high grades in ascending order of quality on the *y*-axis. Within each block, the articles are represented by white and black beads with a unique identification number for each article. These numbers are the same as provided in the table describing their characteristics and across other supplementary material. Black-colored beads represent the articles with registered protocols and white-colored beads represent the unregistered studies. These beads are arranged according to the year of publication and their quality. Visualization of similarities (VOS) viewer software (VOSviewer version 1.6.18, Leiden University, The Netherlands) was used to diagrammatically present the co-occurrence of domains and subdomains of the included studies [20]. Co-occurrence (proportionate to the size of the circle) represents the number of studies including the particular domain and subdomain, whereas the link strength (thickness of the connecting line between two domains and/or subdomains) represents the association between connected circles. The diagrammatic representation uses various colors and circles representing various domains and subdomains. Due to the very large volume of data, this innovative modality was used to assess various areas of CLP at a glance.

## 3. Results

**Figure 2:**
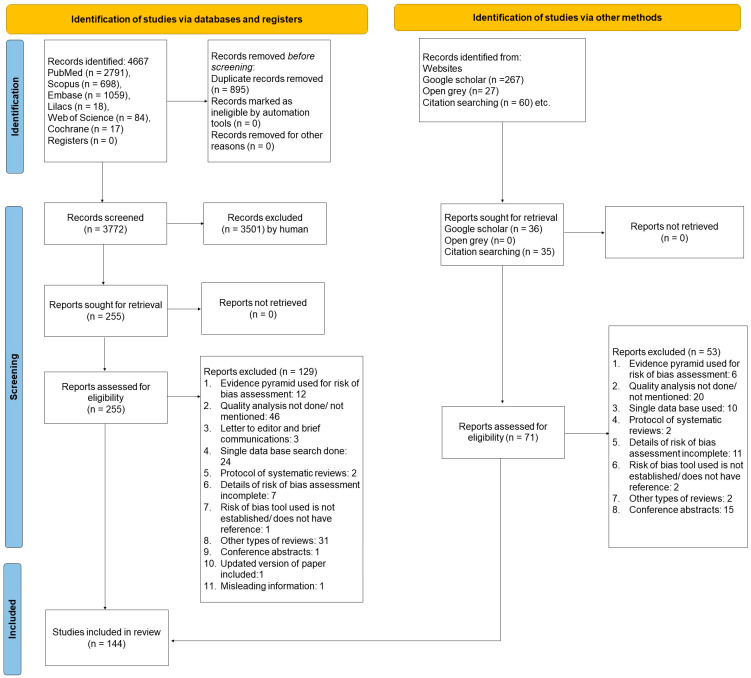
PRISMA 2020 flowchart including searches of databases, registers, and other sources.

A total of 4667 records were identified from the databases and registers. Initial scrutiny was carried out on 3772 titles and abstracts, followed by full-text evaluation of 255 articles. A total of 126 articles were found to be eligible for inclusion. The gray literature search identified 36 records, out of which full-text scrutiny of 15 was performed. Similarly, 35 articles were identified from reference searching and were further scrutinized. These steps resulted in the inclusion of 18 more SRs. Altogether, 144 SRs and MAs [21,22,23,24,25,26,27,28,29,30,31,32,33,34,35,36,37,38,39,40,41,42,43,44,45,46,47,48,49,50,51,52,53,54,55,56,57,58,59,60,61,62,63,64,65,66,67,68,69,70,71,72,73,74,75,76,77,78,79,80,81,82,83,84,85,86,87,88,89,90,91,92,93,94,95,96,97,98,99,100,101,102,103,104,105,106,107,108,109,110,111,112,113,114,115,116,117,118,119,120,121,122,123,124,125,126,127,128,129,130,131,132,133,134,135,136,137,138,139,140,141,142,143,144,145,146,147,148,149,150,151,152,153,154,155,156,157,158,159,160,161,162,163,164] were included in this EM (Figure 2). The list of excluded articles with reasons is provided as Supplementary Material S3.

### 3.1. Characteristics of the Included Studies

The included SRs and MAs were published between January 2008 to February 2022. There was an increasing trend in their numbers with time. The maximum number of published articles were from the year 2021 (n = 29) [28,46,47,48,55,61,62,63,64,65,69,76,81,82,99,100,101,102,111,128,129,130,142,143,145,148,153,154,161]. It was observed that only three studies performed before 2010 could qualify during the two-stage scrutiny [72,112,113]. Among the journals, the highest number of papers (n = 30) was published in the Cleft Palate Craniofacial Journal (CPCJ) [21,35,46,47,48,49,63,65,66,76,86,92,94,100,101,115,116,121,123,125,128,137,140,142,146,150,152,155,161,162] followed by The Cochrane Library (n = 8) [9,16,68,88,92,93,100,106] and the International Journal of Oral and Maxillofacial Surgery (n = 7) [31,72,89,93,106,110,135]. There was diversity among the journals of different specialties that had published the SRs related to CLP (Supplementary Material S4). The number of reviewers involved in the SRs ranged from 2 (n = 6) [63,110,114,121,134,137] to 15 (n = 1) [41]. Language restrictions in inclusion criteria were observed in 67 studies [23,25,26,27,28,32,34,37,40,47,48,49,57,59,60,61,63,64,65,71,72,73,74,75,76,77,83,85,90,92,94,95,96,99,100,102,105,106,110,111,114,116,118,119,122,125,127,129,130,131,133,134,135,140,141,142,143,145,147,150,151,152,153,155,156,162,164] and time-related restrictions (publication year/date) in 22 studies [21,22,24,27,28,37,38,40,47,61,69,78,80,105,109,130,131,138,151,156,160,162] while the remaining 77 SRs had not used any restrictions in their inclusion criteria. A search for gray literature was performed in 83 SRs [22,25,26,27,28,29,31,35,36,37,38,39,40,41,43,44,45,50,54,56,57,60,62,64,66,67,68,76,77,79,80,81,82,83,84,87,88,89,91,93,99,102,103,104,105,108,109,111,112,113,114,115,116,117,118,119,120,121,122,123,124,125,126,128,132,133,134,138,141,142,143,144,147,148,149,151,153,154,156,157,160,161,163] while three SRs had less clarity in this respect [45,96,109]. Hand searching and reference searching were performed in 117 reviews [21,22,23,25,26,28,29,30,31,32,33,34,35,36,37,38,39,40,41,42,44,46,47,48,49,50,51,52,53,54,55,57,58,60,62,64,65,66,67,68,69,70,71,72,74,75,77,78,79,80,81,82,83,84,85,87,88,89,90,91,92,93,95,97,98,102,103,104,107,108,109,110,111,112,113,114,115,116,117,119,120,121,122,123,124,125,126,127,128,130,132,133,134,135,137,138,140,141,142,143,144,146,147,148,149,150,151,152,153,154,156,157,158,159,160,161,163]. The number of databases searched ranged from 2 (n = 18) [22,23,27,34,35,37,38,40,45,48,61,92,102,109,130,138,142,143] to 10 (n = 3) [113,114] and the number of primary studies included in the SRs and MAs ranged from 1 [112,113,126,147] to 144 [58]. There was variability in the methods used for quality analysis/ROB with the Cochrane Collaboration tool being the most popular method used (n = 33) [21,36,41,43,73,75,81,86,88,89,90,94,95,97,108,110,111,112,113,114,115,120,122,123,124,126,130,132,147,149,155,160,163]. This is represented in Supplementary Material S4.

### 3.2. Categorization under Domains and Subdomains

The maximum number of SRs and MAs could be categorized in the therapeutic domain (n = 58) [73,74,75,76,77,78,79,80,81,82,83,84,85,86,87,88,89,90,91,92,93,94,95,96,97,98,99,100,101,102,103,104,105,106,107,108,109,110,111,112,113,114,115,116,117,118,119,120,121,122,123,124,125,126,127,128,129,130], followed by the epidemiologic (n = 46) [21,22,23,24,25,26,27,28,29,30,31,32,33,34,35,36,37,38,39,40,41,42,43,44,45,46,47,48,49,50,51,52,53,54,55,56,57,58,59,60,61,62,63,64,65,66] and prognostic (n = 15) [131,132,133,134,135,136,137,138,139,140,141,142,143] domains. The lowest number of studies (n = 2) was seen in the domains of research methods [161,162] and recent advances [163,164]. The detailed categorization of SRs and MAs in primary and secondary subdomains is presented in Supplementary Material S4.

### 3.3. Co-Occurrence and Link Strength of Domains and Subdomains

**Figure 3:**
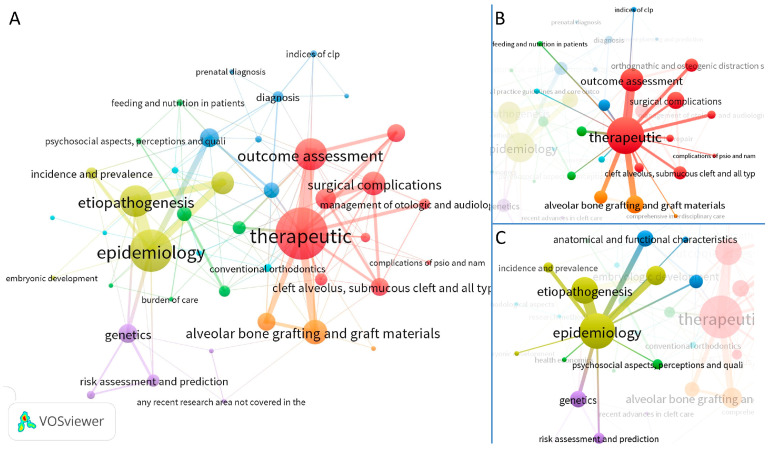
Visual map showing the relationship of domains and subdomains with each other using VOS viewer software. Co-occurrence (proportionate to the size of the circle) represents the number of studies including the particular domain and subdomain, whereas the link strength (thickness of the connecting line between two domains and/or subdomains) represents the association between connected circles. The diagrammatic representation uses various colors and circles representing various domains and subdomains. (**A**) co-occurrence and link strength between domains and subdomains; (**B**) link strength of the therapeutic domain with other domains and subdomains, (**C**) link strength of the epidemiologic domain with other domains and subdomains.

The co-occurrence and link strength of the domains and subdomains of the SRs were represented using a visual map in Figure 3A, using VOS viewer software. It revealed the therapeutic domain as the most recurrent domain shown by the largest size of the representative circle in the visual map. This was followed by the epidemiologic domain as the second largest domain. The co-occurrence and link strength of the epidemiologic and therapeutic domains with other domains and subdomains is shown in Figure 3B,C.

### 3.4. Detailed Description of Studies under the Epidemiologic Domain

**Figure 4:**
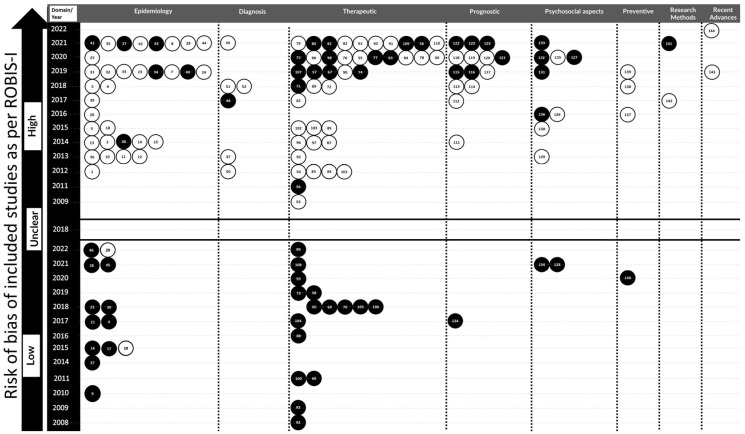
Abacus evidence mapping (EM) plot showing risk of bias of systematic reviews as per ROBIS-I, with three blocks representing low, unclear, and high risk of bias on the *y*-axis, and domain on the *x*-axis. Within each block, the articles are represented by white and black beads with a unique identification number for each article. These numbers are the same as provided in the table describing their characteristics and across other supplementary material. These beads are arranged according to the year of publication. Black beads represent studies that were registered in PROSPERO or similar registries while white beads represent unregistered studies.

**Figure 5:**
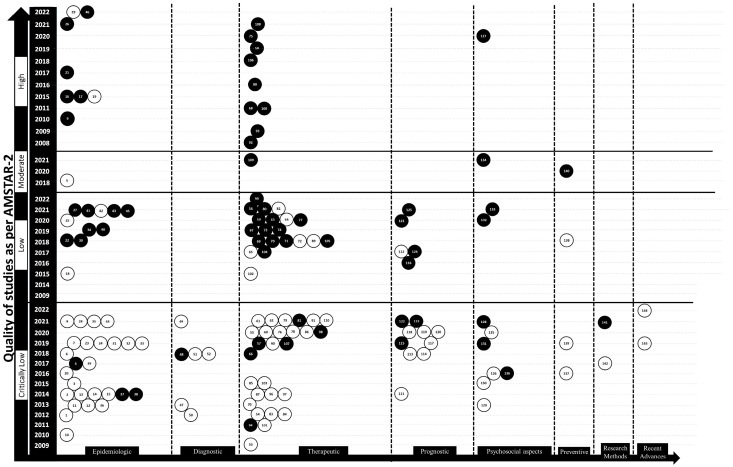
Abacus evidence mapping (EM) plot showing quality of systematic reviews as per AMSTAR 2, with four blocks representing critically low, low, moderate, and high grades in ascending order on the *y*-axis, and domain on the *x*-axis. Within each block, the articles are represented by white and black beads with a unique identification number for each article. These numbers are the same as provided in the table describing their characteristics and across other supplementary material. These beads are arranged according to the year of publication. Black beads represent studies that were registered in PROSPERO or similar registries while white beads represent unregistered studies.

The study characteristics under this domain and their further categorization under various subdomains are detailed in Supplementary Material S4. Among 46 SRs [26,27,28,29,30,31,32,33,34,35,36,37,38,39,40,41,42,43,44,45,46,47,48,49,50,51,52,53,54,55,56,57,58,59,60,61,62,63,64,65,66], only 17 [24,29,36,37,41,42,46,47,50,54,57,58,60,61,63,66] registered their protocol prior to conducting the study (Figure 4). Meta-analysis was performed in 34 of the SRs [21,22,23,24,25,26,28,30,31,33,34,35,36,37,38,39,40,41,42,44,45,46,48,49,50,51,52,53,55,56,57,62,63,66] (Supplementary Material S4). As per ROBIS-I, high ROB was observed in 33 SRs [21,22,23,25,26,27,28,30,31,32,33,34,35,38,40,43,44,45,47,48,51,52,53,54,55,56,58,59,60,61,62,63,64] (Figure 4) and low ROB in 13 SRs [24,29,36,37,39,41,42,46,49,50,57,65,66]. Similarly, as per AMSTAR 2, 11 were found to be of low quality [38,42,45,47,50,54,60,61,62,63,65] and 26 were graded as critically low [21,22,23,24,26,27,28,30,31,32,33,34,35,40,43,44,48,51,52,53,55,56,57,58,59,64] in quality (Figure 5). Furthermore, PRISMA scores showed that 31 SRs had low RE [21,22,23,24,25,32,33,34,36,37,38,39,41,42,45,46,47,48,49,50,53,54,55,57,58,59,60,62,63,64,66] (Supplementary Material S6). Five studies [22,32,61,64,65] in this domain were observed to be inconclusive (Supplementary Material S5).

### 3.5. Detailed Description of Studies under the Diagnostic Domain

The study characteristics under this domain and their further categorization under various subdomains are detailed in Supplementary Material S4. Among six SRs [67,68,69,70,71,72], only one [68] had registered its protocol prior to conducting the study (Figure 4). Meta-analysis had been performed in only one SR [69]. All the studies had high ROB (Figure 4) as per ROBIS-I and were graded as critically low as per AMSTAR 2 (Figure 5) [67,68,69,70,71,72]. Similarly, one SR had low RE [68] and five had moderate RE (Supplementary Material S6) [67,69,70,71,72]. In this domain, three SRs [67,69,72] were conclusive, two [70,71] were partially conclusive, whereas one study [68] was inconclusive (Supplementary Material S5).

### 3.6. Detailed Description of Studies under the Therapeutic Domain

The study characteristics under this domain and their further categorization under various subdomains are detailed in Supplementary Material S4. Among 58 SRs [67,68,69,70,71,72], only 30 [76,77,78,79,83,86,87,88,89,90,91,93,94,95,97,100,101,108,112,113,114,118,119,120,124,125,126,127,128,129] had their protocol registered prior to conducting the study (Figure 4). Meta-analysis was performed in 21 SRs [76,78,81,83,84,88,89,90,91,92,98,99,105,106,108,111,115,119,124,125,129] (Supplementary Material S4). High ROB was found in 42 of them [73,74,75,76,77,80,81,82,83,84,85,87,91,92,94,95,96,97,98,99,100,101,102,103,104,105,106,107,109,110,111,114,115,116,117,118,121,122,123,127,129,130] as per ROBIS-I (Figure 4). As per AMSTAR 2, 28 SRs [73,74,75,77,80,81,82,86,96,98,99,101,103,104,105,106,107,110,111,114,115,116,117,118,121,123,127,130] were graded as critically low in quality and 20 were graded as low quality [76,79,83,84,85,87,89,90,91,92,93,94,97,100,102,109,119,122,124,125] (Figure 5). PRISMA scores revealed that 36 SRs had low RE [73,74,75,76,77,78,79,81,82,86,87,88,89,90,91,93,94,97,98,100,101,106,107,108,111,112,113,114,117,119,120,124,125,126,127,128] (Supplementary Material S6). In this domain, 27 SRs [74,77,79,82,85,86,87,88,92,101,108,109,110,112,113,114,115,116,117,118,121,122,123,125,126,127,128] were inconclusive (Supplementary Material S5).

### 3.7. Detailed Description of Studies under the Prognostic Domain

The study characteristics under this domain and their further categorization under various subdomains are detailed in Supplementary Material S4. Among 15 SRs [131,132,133,134,135,136,137,138,139,140,141,142,143,144,145], only 7 [135,136,141,142,143,144,145] had their protocol registered prior to conducting the study (Figure 4). Meta-analysis was performed in 6 of the studies [133,140,142,143,144,145] (Supplementary Material S4). High ROB was observed in 14 studies [131,132,133,134,135,136,137,138,139,140,141,142,143,145] as per ROBIS-I (Figure 4) and 10 were graded as critically low [131,133,134,135,137,138,139,140,142,143] in quality as per AMSTAR 2 (Figure 5). As per the PRISMA scores, 13 studies had low RE [131,132,133,135,136,137,138,139,140,141,142,143] (Supplementary Material S6). In this domain, two studies [133,142] were found to be inconclusive (Supplementary Material S5).

### 3.8. Detailed Description of Studies under the Psychosocial Aspects, Perceptions and Quality of Life Domain

The study characteristics under this domain and their further categorization under various subdomains are detailed in Supplementary Material S4. Among 11 SRs [146,147,148,149,150,151,152,153,154,155,156], 7 [147,148,151,152,153,154,156] had their protocol registered prior to conducting the study (Figure 4). Meta-analysis was performed in three of the studies [150,153,154]. High ROB was observed in nine SRs [146,147,149,150,151,152,153,155,156] as per ROBIS-I (Figure 4) and seven SRs were graded as critically low in quality as per AMSTAR 2 (Figure 5) [146,148,149,150,151,155,156]. Similarly, five SRs had low RE [147,148,153,154,156] and six had moderate RE [146,149,150,151,152,155] (Supplementary Material S6). In this domain, three SRs were inconclusive (Supplementary Material S5).

### 3.9. Detailed Description of Studies under the Preventive Domain

The study characteristics under this domain and their further categorization under various subdomains are detailed in Supplementary Material S4. Among four SRs [157,158,159,160], only one [160] had its protocol registered before commencing the study (Figure 4). High ROB was observed in three SRs [157,158,159] as per ROBIS-I (Figure 4) and two [157,159] were graded as critically low as per AMSTAR 2 (Figure 5). All four included studies had moderate RE (Supplementary Material S6). In this domain, all four SRs were partially conclusive (Supplementary Material S5) [157,158,159,160].

### 3.10. Detailed Description of Studies under the Research Methods Domain

The study characteristics under this domain and their further categorization under subdomains are detailed in Supplementary Material S4. Among the two SRs [161,162], one [161] had its protocol registered before commencing the study (Figure 4). Both of the SRs had high ROB as per ROBIS-I (Figure 4) and were graded as critically low as per AMSTAR 2 (Figure 5). As per PRISMA reporting scores, one study each had low RE and moderate RE (Supplementary Material S6). In this domain, one SR was conclusive [162] and the other [161] was partially conclusive (Supplementary Material S5).

### 3.11. Detailed Description of Studies under the Recent Advances in the Cleft Care Domain

The two SRs in this domain [163,164] were published in 2019 and 2022, and neither registered the protocol prior to commencing the study. A meta-analysis was conducted by Shanbhag et al. [163]. Both the studies had high ROB as per ROBIS-I (Figure 4) and were graded as critically low in quality as per AMSTAR 2 (Figure 5). As per PRISMA reporting scores, both studies had moderate RE (Supplementary Material S6). In this domain, one SR was conclusive [164], whereas the other [163] was inconclusive (Supplementary Material S5).

The registries of PROSPERO, Joanna Briggs Institute, Cochrane, and Open Science Framework showed that there were altogether 118 protocols registered and ongoing in the field of cleft lip and palate (Supplementary Material S7). The protocols were categorized into various domains and subdomains and it was found that most of the ongoing studies belonged to the therapeutic domain (n = 41) followed by the prognostic domain (n = 27) and the epidemiologic domain (n = 23). Only 3 studies each were categorized under the domains of ‘research methods’ and ‘recent advances’.

## 4. Discussion

In the era of evidence-based treatment protocols, it is essential to evaluate the level of evidence that exists in the different aspects related to CLP. The present study was designed with this rationale in mind with a carefully designed protocol for reducing all possible sources of bias and limitations. Since CLP is a field involving multiple specialties, it was not surprising to find a large number of SRs from diverse disciplines [21,22,24,25,28,29,30,33,34,39,42,43,52,54,56,57,59,60,61,64,69,73,77,90,95,107,148]. It was an extensive task to identify the domains and subdomains of this field and categorize the included SRs. However, the expert panel attempted to reduce the subjectivity in the vital step of categorization of SRs by developing a newer and more precise method. The concept of a secondary subdomain based on the intervention/exposure/secondary outcome could easily justify the SR categorization. The comprehensive search strategy and strict eligibility criteria ensured the inclusion of only methodologically-sound articles.

Based on the categorization of the conclusions of SRs, 27% of SRs (n = 39) were found to be inconclusive, 40% (n = 57) as partially conclusive, and only 33% SRs (n = 48) as conclusive. However, this phenomenon of lack of conclusiveness can be attributed to multiple reasons, most importantly to the significant paucity in the existing CLP data, both at the primary and secondary levels. One of the important aspects of this finding is that it has the potential to guide future CLP researchers to the domains and subdomains with inconclusive studies, find the lacunae, and perform studies to improve the quality of evidence in the future. Similarly, the analysis presented in this evidence mapping shall also act as a ready reckoner for researchers to avoid performing SRs in areas with deficient data. Overall, this shall help minimize the noise due to the excessive number of inconclusive SRs in relation to CLP.

The maximum number of SRs belonged to the therapeutic (40.3%) and epidemiologic domains (30.9%). This indicates the obvious tendency of contemporary researchers to find the need to establish the prevalence, anatomical aspects, etiopathogenesis, or clinical aspects related to management, ranging from pre-surgical infant orthopedics to surgical protocols, cleft orthodontics, and other accessory issues. 

It was interesting to find a greater number of SRs on alveolar bone grafting and the etiopathogenesis of CLP as compared to other subdomains. This highlights the lack of conclusive evidence related to questions such as the choice of graft materials, techniques, or timing, along with the prognosis of each protocol. Similarly, a large number of SRs in relation to the etiopathogenesis of CLP can be expected due to the abundance of original studies presenting data from global cleft registries and the etiological aspects of this developmental anomaly. Further, understanding the etiopathogenesis of a disease is one of the basic research areas that needs to be addressed. Since the first SRs in this domain date back to 2010, the time span of more than a decade could also account for the increased number of papers in this domain. The ongoing studies reflect the growing trend of research interest in the prognostic domain; however, the therapeutic domain still remains the mainstay when compared to all other domains. It appears that the most common focus for researchers at present is the comparison of the efficacy of different treatment modalities, methods, or protocols as well as their long-term effects and complications. The lowest number of SRs was found in the domain of ‘recent advances’. This is a matter of fact because there are not many primary studies in order to conduct such a SR.

One of the first steps in conducting a SR is to register the study protocol in registries such as PROSPERO prior to conducting the SR. This increases the transparency of the methods and allows appropriate analysis after the work has been published. It was very surprising to note that many SRs in the various domains of CLP did not have an a priori registration of their protocols. Moreover, this aspect was common even in the studies published in well-read and high-impact journals. It was even more interesting to observe that even the studies published within the last 5 years had not registered their protocols in PROSPERO or any other registries. This not only raises concerns over the methodological robustness of these studies but also could be a reason for the duplication of SRs on the same topic.

The VOS viewer software is a newer aid for performing bibliometric analyses [20]. It is able to assess the co-occurrence and the strength of links in the keywords and other characteristics of included studies. This visual method was innovatively employed to present the relationships between the domains and subdomains of the included articles. These diagrams also reinforce the dominance of the therapeutic and epidemiologic domains. Furthermore, complex interrelations are observed like a network-web, highlighting the ubiquitous interdisciplinary nature of CLP.

Wormald and Evans in 2018 stated that SRs might suffer from several shortcomings and must not be blindly accepted as the highest clinically relevant source of evidence [165]. Several research groups have attempted to provide tools or checklists for critically analyzing the quality of SRs [10,18,19]. The present study utilized three such methods to adequately evaluate the methodological strengths and weaknesses of the included SRs [10,18,19]. The ROBIS-I tool, introduced in 2016, was used to assess the ROB of the SRs through a three-stage process [18]. It was found that 77% of the SRs exhibited high ROB. This trend continued across all the domains with low ROB ranging from 0 to 28% (Figure 4). The most common paucities observed in SRs, as per ROBIS-I, were the lack of pre-defined objectives and eligibility criteria, restrictions in eligibility criteria based on sources of information, lack of efforts made to minimize error in ROB assessment, heterogeneity of the primary studies, and ROB not detailed. AMSTAR -2 is another method that analyses the quality of SRs through a series of critical and non-critical questions [19]. It was found that 85% of the included studies were either critically low or low in quality, while high-quality studies made up only 12.5%. Among the various domains, the proportion of critically low studies ranged from 60 to 100%. The majority of the included SRs were found to have low REs (Supplementary Material S6). Previous work in dental traumatology utilized ROBIS-I and PRISMA [8]. The study by Madera et al. used AMSTAR 2 as the method of quality analysis of SRs [7]. Another EM in pediatric dentistry by Mejàre et al. also used AMSTAR 2 for assessing the ROB of included studies in their EM study [9]. The analysis of quality, assessment of ROB, and reporting errors in the present EM through multiple tools presents the quality of evidence and identifies methodological paucities of the existing SRs to a greater extent, which is not habitually carried out. There exists a thin line between quality analysis and assessment of ROB and they are often used interchangeably in several studies. Though both tools have similar parameters, ROBIS-I provides a stepwise protocol for assessing the sources of bias, and AMSTAR 2 helps in understanding the critical threats to the quality. The quality of the studies as interpreted by AMSTAR 2 was cross-checked and verified against the ROB using the ROBIS-I tool, increasing the robustness of the methodology and making the evidence mapping more concrete and reliable.

The findings of the 144 included SRs were too extensive to present as a table; hence, the expert group devised a method called Abacus EM Plot which was a modification of a previously used Bubble Diagram [8]. This could effectively summarize the findings of the three quality-assessment methods and provide information about the distribution of SRs in terms of domains, year of publication, and protocol registration. This is another strength of this evidence mapping where a large amount of data has been compared using a clear diagrammatic representation. A unique addition to this schema was a column with year of publication as shown in Figure 4 and Figure 5. This could further improve the systematic arrangement of studies in descending order of year from top to bottom in various categories. It was also noted that the SRs showed variation in the quality scores when assessed by the three tools and leads to another research question if such differences are statistically significant or not. Furthermore, evaluation of ROB and quality of the studies emphasized the need for improvement of their quality by addressing the deficiencies highlighted in this evidence mapping. 

From the current evidence, we could also find that there have been various studies focusing on a specific cleft phenotype. This is much needed since we lack evidence of the efficacy of certain treatment modalities in specific phenotypes or sub-phenotypes of CLP. Presenting data for CLP as a whole might not make much sense since there are important differences between different phenotypes, Although there are various challenges in studying these phenotypes individually [166], future studies in CLP should focus on finding evidence for specific phenotypes.

Any EM can suffer from limitations, including an incomplete literature search, biased inclusion criteria, and the subjectivity of the quality-assessment tools. Although careful planning and execution were the hallmarks of the present study, the complete absence of these potential limitations cannot be guaranteed. The outcomes of this study are dependent upon the last date of the literature search and hence require a periodic update.

## 5. Conclusions

The majority of included SRs were categorized in the therapeutic and prognostic domains, with most of them showing high ROB and critically-low quality regardless of the source journal. It is essential to fill the existing gaps with good-quality studies because these domains are directly related to the care of patients CLP and special healthcare needs. The clinical care of patients with CLP and the development of evidence-based protocols are also dependent upon the presence of high-quality data. This evidence mapping attempts to provide more clarity on the quality of existing evidence in CLP, and helps researchers identify the paucities in existing inconclusive SRs. These results might serve as a starting point for the global interdisciplinary CLP research community and encourage high-quality evidence-based research.

## Figures and Tables

**Table 1:** Domains and subdomains related to cleft lip and palate. A specific alphabet and color coding were created for different domains and subdomains under them. For example, the domain of ‘Epidemiology’ and its subdomains have a light blue color with code A assigned to the domain and subdomains as A1–A6.

Domains and Subdomains Related to Cleft Lip and Palate			
Code	Domain	Code	Subdomain
**A**	Epidemiologic	**A1**	Incidence and prevalence
		**A2**	Etiopathogenesis
		**A3**	Embryologic development
		**A4**	Genetics
		**A5**	Syndromes and anomalies of head and neck in relation to CLP
		**A6**	Anatomical and functional characteristics
**B**	Diagnostic	**B1**	Classifications
		**B2**	Indices of CLP
		**B3**	Prenatal diagnosis
		**B4**	Clinical diagnostic methods
		**B5**	Conventional radiography and 2D imaging
		**B6**	Advanced radiography including 3D imaging and 3D printing
		**B7**	Photography and records
**C**	Therapeutic	**C1**	Treatment planning and prediction
		**C2**	Feeding and nutrition in patients
		**C3**	Presurgical infant orthopedics (PSIO) and naso-alveolar molding (PNAM)
		**C4**	Early orthodontics
		**C5**	Orthodontic and surgical perspectives of secondary alveolar bone grafting
		**C6**	Conventional orthodontics
		**C7**	Late and retreatment adult orthodontics
		**C8**	Cleft lip repair
		**C9**	Cleft alveolus, submucous cleft, and all types of cleft palate closure/repair
		**C10**	Alveolar bone grafting and graft materials
		**C11**	Orthognathic and osteogenic distraction surgical orthodontics
		**C12**	Other surgical aspects of cleft lip and palate including revisions, scar management, post-operative pain, and anesthesia
		**C13**	Comprehensive interdisciplinary care
		**C14**	Management of speech and hearing problems
		**C15**	Outcome assessment
**D**	Prognostic	**D1**	Effects/complications of untreated CLP
		**D2**	Effects/complications of PSIO and PNAM
		**D3**	Effects/complications of orthodontic treatment
		**D4**	Effects/complications of surgical treatment and/or alveolar bone grafting
		**D5**	Obstructive sleep apnea
		**D6**	Oral health aspects including dental caries and other dental problems
		**D7**	Systemic problems
**E**	Psychosocial aspects, perceptions and quality of life	**E1**	Psychosocial aspects, perceptions and quality of life of family members of the patients with CLP, lay people, and healthcare professionals
		**E2**	Psychosocial aspects, perceptions and quality of life of patients with CLP
		**E3**	Burden of care
		**E4**	Health economics
		**E5**	Support groups and charity associations
**F**	Preventive	**F1**	Genetic counselling
		**F2**	Risk assessment and prediction
		**F3**	Public awareness
		**F4**	Patient awareness
		**F5**	Awareness of medical and dental specialists
**G**	Research methods	**G1**	Clinical practice guidelines and core outcome sets
		**G2**	Ethical aspects of research
		**G3**	Methodological aspects
**H**	Recent advances in cleft care	**H1**	Any recent research area not covered in the above categorization
**I**	Others	**I1**	Any area not covered in above categorization

## Data Availability

All the data generated have been included in the article.

## Supplementary Materials

The following supporting information can be downloaded at: https://www.mdpi.com/article/10.3390/jcm12186002/s1, Supplementary Material S1: Flowchart showing tasks performed in evidence mapping of systematic reviews in cleft lip and palate. Supplementary Material S2a: Details of the Population (P), Exposure (E), Comparator (C), Outcome (O) elements of the research question, the search strategy, and inclusion and exclusion criteria. Supplementary Material S2b: Detailed search strategy for each database. Supplementary Material S3: List of excluded articles with reasons. Supplementary Material S4: Detailed characteristics of the included studies including author, year, journal of publication, number of reviewers, language limits, year of publication limit, hand/reference searching, gray literature search, number of databases used, number of included studies, risk of bias tool used for quality assessment and meta-analysis (abbreviations: Y = present; N = not present). Supplementary Material S5: Detailed characteristics of the included studies including author, year, aims, and objectives in P (Population) I/E (Intervention/exposure) C (Comparison) O (Outcome), and grade of conclusion. Supplementary Material S6: Abacus evidence mapping (EM) plot showing PRISMA reporting scores of systematic reviews. Supplementary Material S7: Table showing details of ongoing studies registered in PROSPERO and their categorization as domains and subdomains of cleft lip and palate.
