# Supplementary material for: Evidence Mapping and Quality Analysis of Systematic Reviews on Various Aspects Related to Cleft Lip and Palate
Source: J Clin Med. 2023 Sep 16;12(18):6002. doi: 10.3390/jcm12186002 (PMC10532364; doi:10.3390/jcm12186002)
Supplement: Supplementary file 1 [file jcm-12-06002-s001.zip › Annexure 2.pdf]

Annexure 2(a): Details of the Population (P), Exposure (E), Comparator (C), Outcome (O) elements of the research question, the search strategy, and inclusion & exclusion criteria.

**PICO elements of the Research Question**	
**Participants/population:** Individuals with cleft lip and palate<br>**Intervention(s), exposure(s):** Diagnostic and management protocols along with follow up assessments<br>**Comparator(s)/control:** Not required<br>**Outcome:**<br>**Main outcomes:**<br>1. Systematic Mapping of the domains related to Cleft lip and palate with available systematic reviews.<br>2. Demographic mapping of the reviews in terms of journal, region and year<br>3. Category wise assessment of systematic reviews in terms of demographics, number of reviewers, speciality of reviewers, clear definition of review methods, use of endo note, risk of bias assessment: tool, number of assessors, meta-analysis performed: model used, types of plots, barriers in performing it.<br>4. Research question of reviews in terms of PICO.<br>5. Quality assessment of the systematic reviews.<br>**Additional outcome(s).**<br>1. Identification of the domains which do not have systematic reviews.<br>2. Identification of the domains which have systematic reviews with high risk of bias.<br>3. Future directions for the systematic reviews and researches to be done related to Cleft lip and palate.	
**Search Strategy**	
<u>Field 1:</u><br>Cleft Lip and Palate OR Oral Cleft OR Cleft OR Cleft Palate OR Facial Cleft OR Cleft Lip OR Cleft Alveolus<br><u>Field 2:</u><br>Prevalence OR Incidence OR Epidemiology OR Etiology OR Risk factors OR Classification OR Imaging OR Advanced Imaging OR PreSurgical Naso Alveolar Molding (PNAM) OR Surgical Repair OR Timing of surgical treatment OR obturator OR Alveolar grafting OR Bone grafting OR Caries OR Orthodontic Treatment OR Timing of Orthodontic Treatment OR Surgical Healing OR Complications OR relapse OR Feeding OR Psychological aspects OR Behavioural Problems OR Quality of Life OR Oral Health Related Quality<br><u>Field 3:</u><br>Systematic review OR review or meta analysis	
**Inclusion Criteria**	**Exclusion Criteria**
1. Systematic Reviews done in any domain related to Cleft lip and palate.<br>2. Clearly defined search strategy with involvement of two or more reviewers.<br>3. Search performed in at least 2 search engines.<br>4. Quality assessment of the included studies should have been performed by using a validated tool or a self-designed checklist based on a credible source of appraisal of articles.	1. The reviews performed on the studies where risk of bias assessment of included studies was done using evidence pyramid.

5. The most recent/updated review from the same research group will be included.	

Annexure 2(b): Detailed search strategy for each database

PUBMED- Number of results: 2791

((("cleft lip"[MeSH Terms] OR ("cleft"[All Fields] AND "lip"[All Fields]) OR "cleft lip"[All Fields]) AND ("palatalization"[All Fields] OR "palatalized"[All Fields] OR "palatally"[All Fields] OR "palatals"[All Fields] OR "palate"[MeSH Terms] OR "palate"[All Fields] OR "palatal"[All Fields] OR "palates"[All Fields])) OR (("mouth"[MeSH Terms] OR "mouth"[All Fields] OR "oral"[All Fields]) AND ("cleft"[All Fields] OR "clefted"[All Fields] OR "clefting"[All Fields] OR "clefts"[All Fields])) OR ("cleft"[All Fields] OR "clefted"[All Fields] OR "clefting"[All Fields] OR "clefts"[All Fields]) OR ("cleft palate"[MeSH Terms] OR ("cleft"[All Fields] AND "palate"[All Fields]) OR "cleft palate"[All Fields]) OR (("face"[MeSH Terms] OR "face"[All Fields] OR "facial"[All Fields] OR "facials"[All Fields]) AND ("cleft"[All Fields] OR "clefted"[All Fields] OR "clefting"[All Fields] OR "clefts"[All Fields])) OR ("cleft lip"[MeSH Terms] OR ("cleft"[All Fields] AND "lip"[All Fields]) OR "cleft lip"[All Fields]) OR (("cleft"[All Fields] OR "clefted"[All Fields] OR "clefting"[All Fields] OR "clefts"[All Fields]) AND ("tooth socket"[MeSH Terms] OR ("tooth"[All Fields] AND "socket"[All Fields]) OR "tooth socket"[All Fields] OR "alveolus"[All Fields]))) AND ((("epidemiology"[MeSH Subheading] OR "epidemiology"[All Fields] OR "prevalence"[All Fields] OR "prevalence"[MeSH Terms] OR "prevalance"[All Fields] OR "prevalences"[All Fields] OR "prevalence s"[All Fields] OR "prevalent"[All Fields] OR "prevalently"[All Fields] OR "prevalents"[All Fields] OR ("epidemiology"[MeSH Subheading] OR "epidemiology"[All Fields] OR "incidence"[All Fields] OR "incidence"[MeSH Terms] OR "incidences"[All Fields] OR "incident"[All Fields] OR "incidents"[All Fields]) OR ("epidemiologies"[All Fields] OR "epidemiology"[MeSH Subheading] OR "epidemiology"[All Fields] OR "epidemiology"[MeSH Terms] OR "epidemiology s"[All Fields]) OR ("aetiologie"[All Fields] OR "aetiologies"[All Fields] OR "aetiology"[All Fields] OR "etiologies"[All Fields] OR "etiology"[MeSH Subheading] OR "etiology"[All Fields] OR "causality"[MeSH Terms] OR "causality"[All Fields]) OR ("risk factors"[MeSH Terms] OR ("risk"[All Fields] AND "factors"[All Fields]) OR "risk factors"[All Fields]) OR ("classification"[MeSH Terms] OR "classification"[All Fields] OR "classifications"[All Fields] OR "classification"[MeSH Subheading] OR "classification s"[All Fields] OR "classificator"[All Fields] OR "classificators"[All Fields]) OR ("image"[All Fields] OR "image s"[All Fields] OR "imaged"[All Fields] OR "imager"[All Fields] OR "imager s"[All Fields] OR "imagers"[All Fields] OR "images"[All Fields] OR "imaging"[All Fields] OR "imaging s"[All Fields] OR "imagings"[All Fields]) OR ("adv imaging"[Journal] OR ("advanced"[All Fields] AND "imaging"[All Fields]) OR "advanced imaging"[All Fields]) OR (("phys rev e"[Journal] OR "phys rev e stat nonlin soft matter phys"[Journal] OR "pre"[All Fields]) AND ("surgical procedures, operative"[MeSH Terms] OR ("surgical"[All Fields] AND "procedures"[All Fields] AND "operative"[All Fields]) OR "operative surgical procedures"[All Fields] OR "surgical"[All Fields] OR "surgically"[All Fields] OR "surgicals"[All Fields]) AND ("nose"[MeSH Terms] OR "nose"[All Fields] OR "naso"[All Fields]) AND ("alveolar"[All Fields] OR "alveolarization"[All Fields] OR "alveolars"[All Fields]) AND ("fungi"[MeSH Terms] OR "fungi"[All Fields] OR "mold"[All Fields] OR "molds"[All Fields] OR "mould"[All Fields] OR "molded"[All Fields] OR "molding"[All Fields] OR "moldings"[All Fields] OR "moulded"[All Fields] OR "moulding"[All Fields] OR "mouldings"[All Fields] OR "moulds"[All Fields]))) AND "PNAM"[All Fields]) OR (("surgical procedures, operative"[MeSH Terms] OR ("surgical"[All Fields] AND "procedures"[All Fields] AND "operative"[All Fields]) OR "operative surgical procedures"[All Fields] OR "surgical"[All Fields] OR "surgically"[All Fields] OR "surgicals"[All Fields]) AND ("repairability"[All Fields] OR "repairable"[All Fields] OR "repaire"[All Fields] OR "repaired"[All Fields] OR "repairment"[All Fields] OR "wound healing"[MeSH Terms] OR ("wound"[All Fields] AND "healing"[All Fields]) OR "wound healing"[All Fields] OR "repair"[All Fields] OR "repairing"[All Fields] OR "repairs"[All Fields])) OR ("Timing-of-surgical"[All Fields] AND ("therapeutics"[MeSH Terms] OR "therapeutics"[All Fields] OR "treatments"[All Fields] OR "therapy"[MeSH Subheading] OR "therapy"[All Fields] OR "treatment"[All Fields] OR "treatment s"[All Fields])) OR ("obturate"[All Fields] OR "obturated"[All Fields] OR "obturating"[All Fields] OR "obturation"[All Fields] OR "obturations"[All Fields] OR "obturator"[All Fields] OR "obturators"[All Fields] OR "obturing"[All Fields]) OR (("alveolar"[All Fields] OR "alveolarization"[All Fields] OR "alveolars"[All Fields]) AND ("graft s"[All Fields] OR "grafted"[All Fields] OR "graftings"[All Fields] OR "transplantation"[MeSH Subheading] OR "transplantation"[All Fields] OR "grafting"[All Fields] OR "transplantation"[MeSH Terms] OR "grafts"[All Fields] OR "transplants"[MeSH Terms] OR "transplants"[All Fields] OR "graft"[All Fields])) OR ("bone transplantation"[MeSH Terms] OR ("bone"[All Fields] AND "transplantation"[All Fields]) OR "bone transplantation"[All Fields] OR ("bone"[All Fields] AND "grafting"[All Fields]) OR "bone grafting"[All Fields]) OR ("carie"[All Fields] OR "dental caries"[MeSH Terms] OR ("dental"[All Fields] AND "caries"[All Fields]) OR "dental caries"[All Fields] OR "caries"[All Fields]) OR (("orthodontal"[All Fields] OR "orthodontic"[All Fields] OR "orthodontical"[All Fields] OR "orthodontically"[All Fields] OR "orthodontics"[MeSH Terms] OR "orthodontics"[All Fields]) AND ("therapeutics"[MeSH Terms] OR "therapeutics"[All Fields] OR "treatments"[All Fields] OR "therapy"[MeSH Subheading] OR "therapy"[All Fields] OR "treatment"[All Fields] OR "treatment s"[All Fields])) OR "Orthodontic-Treatment-Time"[All Fields] OR (("surgical procedures,

operative"[MeSH Terms] OR ("surgical"[All Fields] AND "procedures"[All Fields] AND "operative"[All Fields]) OR "operative surgical procedures"[All Fields] OR "surgical"[All Fields] OR "surgically"[All Fields] OR "surgicals"[All Fields]) AND ("healed"[All Fields] OR "wound healing"[MeSH Terms] OR ("wound"[All Fields] AND "healing"[All Fields]) OR "wound healing"[All Fields] OR "healing"[All Fields] OR "healings"[All Fields] OR "heals"[All Fields])) OR ("complicances"[All Fields] OR "complicate"[All Fields] OR "complicated"[All Fields] OR "complicates"[All Fields] OR "complicating"[All Fields] OR "complication"[All Fields] OR "complication s"[All Fields] OR "complications"[MeSH Subheading] OR "complications"[All Fields]) OR ("recurrence"[MeSH Terms] OR "recurrence"[All Fields] OR "relapse"[All Fields] OR "relapses"[All Fields] OR "relapsing"[All Fields] OR "relapsed"[All Fields] OR "relapser"[All Fields] OR "relapsers"[All Fields]) OR ("feeding"[All Fields] OR "feedings"[All Fields] OR "feeds"[All Fields]) OR (("psychologic"[All Fields] OR "psychological"[All Fields] OR "psychologically"[All Fields] OR "psychologization"[All Fields] OR "psychologized"[All Fields] OR "psychologizing"[All Fields]) AND ("aspect"[All Fields] OR "aspects"[All Fields])) OR ("problem behavior"[MeSH Terms] OR ("problem"[All Fields] AND "behavior"[All Fields]) OR "problem behavior"[All Fields] OR ("behavioural"[All Fields] AND "problems"[All Fields]) OR "behavioural problems"[All Fields]) OR ("quality of life"[MeSH Terms] OR ("quality"[All Fields] AND "life"[All Fields]) OR "quality of life"[All Fields]) OR (("oral health"[MeSH Terms] OR ("oral"[All Fields] AND "health"[All Fields]) OR "oral health"[All Fields]) AND ("family"[MeSH Terms] OR "family"[All Fields] OR "relation"[All Fields] OR "relatability"[All Fields] OR "relatable"[All Fields] OR "related"[All Fields] OR "relates"[All Fields] OR "relating"[All Fields] OR "relational"[All Fields] OR "relations"[All Fields]) AND ("qualities"[All Fields] OR "quality"[All Fields] OR "quality s"[All Fields]))) AND ("systematic review"[Publication Type] OR "systematic reviews as topic"[MeSH Terms] OR "systematic review"[All Fields] OR ("review"[Publication Type] OR "review literature as topic"[MeSH Terms] OR "review"[All Fields]) OR ("meta analysis"[Publication Type] OR "meta analysis as topic"[MeSH Terms] OR "meta analysis"[All Fields]))

Translations

Cleft Lip: "cleft lip"[MeSH Terms] OR ("cleft"[All Fields] AND "lip"[All Fields]) OR "cleft lip"[All Fields]

Palate: "palatalization"[All Fields] OR "palatalized"[All Fields] OR "palatally"[All Fields] OR "palatals"[All Fields] OR "palate"[MeSH Terms] OR "palate"[All Fields] OR "palatal"[All Fields] OR "palates"[All Fields] OR "palate's"[All Fields]

Oral: "mouth"[MeSH Terms] OR "mouth"[All Fields] OR "oral"[All Fields]

Cleft: "cleft"[All Fields] OR "clefted"[All Fields] OR "clefting"[All Fields] OR "clefts"[All Fields]

Cleft: "cleft"[All Fields] OR "clefted"[All Fields] OR "clefting"[All Fields] OR "clefts"[All Fields]

Cleft Palate: "cleft palate"[MeSH Terms] OR ("cleft"[All Fields] AND "palate"[All Fields]) OR "cleft palate"[All Fields]

Facial: "face"[MeSH Terms] OR "face"[All Fields] OR "facial"[All Fields] OR "facials"[All Fields]

Cleft: "cleft"[All Fields] OR "clefted"[All Fields] OR "clefting"[All Fields] OR "clefts"[All Fields]

Cleft Lip: "cleft lip"[MeSH Terms] OR ("cleft"[All Fields] AND "lip"[All Fields]) OR "cleft lip"[All Fields]

Cleft: "cleft"[All Fields] OR "clefted"[All Fields] OR "clefting"[All Fields] OR "clefts"[All Fields]

Alveolus: "tooth socket"[MeSH Terms] OR ("tooth"[All Fields] AND "socket"[All Fields]) OR "tooth socket"[All Fields] OR "alveolus"[All Fields]

Prevalence: "epidemiology"[Subheading] OR "epidemiology"[All Fields] OR "prevalence"[All Fields] OR "prevalence"[MeSH Terms] OR "prevalance"[All Fields] OR "prevalences"[All Fields] OR "prevalence's"[All Fields] OR "prevalent"[All Fields] OR "prevalently"[All Fields] OR "prevalents"[All Fields]

Incidence: "epidemiology"[Subheading] OR "epidemiology"[All Fields] OR "incidence"[All Fields] OR "incidence"[MeSH Terms] OR "incidences"[All Fields] OR "incident"[All Fields] OR "incidents"[All Fields]

Epidemiology: "epidemiologies"[All Fields] OR "epidemiology"[Subheading] OR "epidemiology"[All Fields] OR "epidemiology"[MeSH Terms] OR "epidemiology's"[All Fields]

Etiology: "aetiologie"[All Fields] OR "aetiologies"[All Fields] OR "aetiology"[All Fields] OR "etiologies"[All Fields] OR "etiology"[Subheading] OR "etiology"[All Fields] OR "causality"[MeSH Terms] OR "causality"[All Fields]

Risk factors: "risk factors"[MeSH Terms] OR ("risk"[All Fields] AND "factors"[All Fields]) OR "risk factors"[All Fields]

Classification: "classification"[MeSH Terms] OR "classification"[All Fields] OR "classifications"[All Fields] OR "classification"[Subheading] OR "classification's"[All Fields] OR "classificator"[All Fields] OR "classificators"[All Fields]

Imaging: "image"[All Fields] OR "image's"[All Fields] OR "imaged"[All Fields] OR "imager"[All Fields] OR "imager's"[All Fields] OR "imagers"[All Fields] OR "images"[All Fields] OR "imaging"[All Fields] OR "imaging's"[All Fields] OR "imagings"[All Fields]

Advanced Imaging: "Adv Imaging"[Journal:__jid9884108] OR ("advanced"[All Fields] AND "imaging"[All Fields]) OR "advanced imaging"[All Fields]

Pre: "Phys Rev E"[Journal:__jid101676019] OR "Phys Rev E Stat Nonlin Soft Matter Phys"[Journal:__jid101136452] OR "pre"[All Fields]

Surgical: "surgical procedures, operative"[MeSH Terms] OR ("surgical"[All Fields] AND "procedures"[All Fields] AND "operative"[All Fields]) OR "operative surgical procedures"[All Fields] OR "surgical"[All Fields] OR "surgically"[All Fields] OR "surgicals"[All Fields]

Naso: "nose"[MeSH Terms] OR "nose"[All Fields] OR "naso"[All Fields]

Alveolar: "alveolar"[All Fields] OR "alveolarization"[All Fields] OR "alveolars"[All Fields]

Moulding: "fungi"[MeSH Terms] OR "fungi"[All Fields] OR "mold"[All Fields] OR "molds"[All Fields] OR "mould"[All Fields] OR "molded"[All Fields] OR "molding"[All Fields] OR "moldings"[All Fields] OR "moulded"[All Fields] OR "moulding"[All Fields] OR "mouldings"[All Fields] OR "moulds"[All Fields]

Surgical: "surgical procedures, operative"[MeSH Terms] OR ("surgical"[All Fields] AND "procedures"[All Fields] AND "operative"[All Fields]) OR "operative surgical procedures"[All Fields] OR "surgical"[All Fields] OR "surgically"[All Fields] OR "surgicals"[All Fields]
Repair: "repairability"[All Fields] OR "repairable"[All Fields] OR "repaire"[All Fields] OR "repaired"[All Fields] OR "repairment"[All Fields] OR "wound healing"[MeSH Terms] OR ("wound"[All Fields] AND "healing"[All Fields]) OR "wound healing"[All Fields] OR "repair"[All Fields] OR "repairing"[All Fields] OR "repairs"[All Fields]
treatment: "therapeutics"[MeSH Terms] OR "therapeutics"[All Fields] OR "treatments"[All Fields] OR "therapy"[Subheading] OR "therapy"[All Fields] OR "treatment"[All Fields] OR "treatment's"[All Fields]
obturator: "obturate"[All Fields] OR "obturated"[All Fields] OR "obturating"[All Fields] OR "obturation"[All Fields] OR "obturations"[All Fields] OR "obturator"[All Fields] OR "obturators"[All Fields] OR "obturing"[All Fields]
Alveolar: "alveolar"[All Fields] OR "alveolarization"[All Fields] OR "alveolars"[All Fields]
grafting: "graft's"[All Fields] OR "grafted"[All Fields] OR "graftings"[All Fields] OR "transplantation"[Subheading] OR "transplantation"[All Fields] OR "grafting"[All Fields] OR "transplantation"[MeSH Terms] OR "grafts"[All Fields] OR "transplants"[MeSH Terms] OR "transplants"[All Fields] OR "graft"[All Fields]
Bone grafting: "bone transplantation"[MeSH Terms] OR ("bone"[All Fields] AND "transplantation"[All Fields]) OR "bone transplantation"[All Fields] OR ("bone"[All Fields] AND "grafting"[All Fields]) OR "bone grafting"[All Fields]
Caries: "carie"[All Fields] OR "dental caries"[MeSH Terms] OR ("dental"[All Fields] AND "caries"[All Fields]) OR "dental caries"[All Fields] OR "caries"[All Fields]
Orthodontic: "orthodontal"[All Fields] OR "orthodontic"[All Fields] OR "orthodontical"[All Fields] OR "orthodontically"[All Fields] OR "orthodontics"[MeSH Terms] OR "orthodontics"[All Fields]
Treatment: "therapeutics"[MeSH Terms] OR "therapeutics"[All Fields] OR "treatments"[All Fields] OR "therapy"[Subheading] OR "therapy"[All Fields] OR "treatment"[All Fields] OR "treatment's"[All Fields]
Surgical: "surgical procedures, operative"[MeSH Terms] OR ("surgical"[All Fields] AND "procedures"[All Fields] AND "operative"[All Fields]) OR "operative surgical procedures"[All Fields] OR "surgical"[All Fields] OR "surgically"[All Fields] OR "surgicals"[All Fields]
Healing: "healed"[All Fields] OR "wound healing"[MeSH Terms] OR ("wound"[All Fields] AND "healing"[All Fields]) OR "wound healing"[All Fields] OR "healing"[All Fields] OR "healings"[All Fields] OR "heals"[All Fields]
Complications: "complicances"[All Fields] OR "complicate"[All Fields] OR "complicated"[All Fields] OR "complicates"[All Fields] OR "complicating"[All Fields] OR "complication"[All Fields] OR "complication's"[All Fields] OR "complications"[Subheading] OR "complications"[All Fields]
relapse: "recurrence"[MeSH Terms] OR "recurrence"[All Fields] OR "relapse"[All Fields] OR "relapses"[All Fields] OR "relapsing"[All Fields] OR "relapsed"[All Fields] OR "relapser"[All Fields] OR "relapsers"[All Fields]
Feeding: "feeding"[All Fields] OR "feedings"[All Fields] OR "feeds"[All Fields]
Psychological: "psychologic"[All Fields] OR "psychological"[All Fields] OR "psychologically"[All Fields] OR "psychologization"[All Fields] OR "psychologized"[All Fields] OR "psychologizing"[All Fields]
aspects: "aspect"[All Fields] OR "aspects"[All Fields]
Behavioural Problems: "problem behavior"[MeSH Terms] OR ("problem"[All Fields] AND "behavior"[All Fields]) OR "problem behavior"[All Fields] OR ("behavioural"[All Fields] AND "problems"[All Fields]) OR "behavioural problems"[All Fields]
Quality of Life: "quality of life"[MeSH Terms] OR ("quality"[All Fields] AND "life"[All Fields]) OR "quality of life"[All Fields]
Oral Health: "oral health"[MeSH Terms] OR ("oral"[All Fields] AND "health"[All Fields]) OR "oral health"[All Fields]
Related: "family"[MeSH Terms] OR "family"[All Fields] OR "relation"[All Fields] OR "relatability"[All Fields] OR "relatable"[All Fields] OR "related"[All Fields] OR "relates"[All Fields] OR "relating"[All Fields] OR "relational"[All Fields] OR "relations"[All Fields]
Quality: "qualities"[All Fields] OR "quality"[All Fields] OR "quality's"[All Fields]
Systematic review: "systematic review"[Publication Type] .or. "systematic reviews as topic"[MeSH Terms] .or. "systematic review"[All Fields]
review: "review"[Publication Type] .or. "review literature as topic"[MeSH Terms] .or. "review"[All Fields]
meta analysis: "meta-analysis"[Publication Type] .or. "meta-analysis as topic"[MeSH Terms] .or. "meta-analysis"[All Fields]

EMBASE – Number of results: 1059

('cleft lip palate':ti,ab,kw OR 'oral cleft':ti,ab,kw OR cleft:ti,ab,kw OR 'cleft palate':ti,ab,kw OR 'facial cleft':ti,ab,kw OR 'cleft lip':ti,ab,kw OR 'cleft alveolus':ti,ab,kw) AND ((prevalence:ti,ab,kw OR incidence:ti,ab,kw OR epidemiology:ti,ab,kw OR etiology:ti,ab,kw OR 'risk factors':ti,ab,kw OR classification:ti,ab,kw OR imaging:ti,ab,kw OR 'advanced imaging':ti,ab,kw OR 'pre surgical naso alveolar moulding':ti,ab,kw) AND pnam:ti,ab,kw OR 'surgical repair':ti,ab,kw OR 'timing-of-surgical treatment':ti,ab,kw OR obturator:ti,ab,kw OR 'alveolar grafting':ti,ab,kw OR 'bone grafting':ti,ab,kw OR caries:ti,ab,kw OR 'orthodontic treatment':ti,ab,kw OR 'orthodontic treatment time':ti,ab,kw OR 'surgical healing':ti,ab,kw OR complications:ti,ab,kw OR relapse:ti,ab,kw OR feeding:ti,ab,kw OR 'psychological aspects':ti,ab,kw OR 'behavioural problems':ti,ab,kw OR 'quality of life':ti,ab,kw OR 'oral health related quality':ti,ab,kw) AND ('systematic review':ti,ab,kw OR review:ti,ab,kw OR 'meta analysis':ti,ab,kw)

WEB OF SCIENCES- Number of results: 84

Cleft Lip Palate OR Oral Cleft OR Cleft OR Cleft Palate OR Facial Cleft OR Cleft Lip OR Cleft Alveolus **(Title) and** Prevalence OR Incidence OR Epidemiology OR Etiology OR Risk factors OR Classification OR Imaging OR Advanced Imaging OR Pre Surgical Naso Alveolar Moulding (pram) OR Surgical Repair OR Timing-of-surgical treatment OR obturator OR Alveolar grafting OR Bone grafting OR Caries OR Orthodontic Treatment OR Orthodontic-Treatment-Time OR Surgical Healing OR Complications OR relapse OR Feeding OR Psychological aspects OR Behavioural Problems OR Quality of Life OR Oral Health Related Quality **(Title) and** Systematic review OR review or meta analysis **(Title)**

LILACS- Number of results: 18

(cleft lip palate OR oral cleft OR cleft OR cleft palate OR facial cleft OR cleft lip OR cleft alveolus) AND (db:("LILACS"))

COCHRANE- Number of results: 17

Cleft Lip Palate OR Oral Cleft OR Cleft OR Cleft Palate OR Facial Cleft OR Cleft Lip OR Cleft Alveolus in Title Abstract Keyword AND Prevalence OR Incidence OR Epidemiology OR Etiology OR Risk factors OR Classification OR Imaging OR Advanced Imaging OR Pre Surgical Naso Alveolar Moulding (PNAM) OR Surgical Repair OR Timing-of-surgical treatment OR obturator OR Alveolar grafting OR Bone grafting OR Caries OR Orthodontic Treatment OR Orthodontic-Treatment-Time OR Surgical Healing OR Complications OR relapse OR Feeding OR Psychological aspects OR Behavioural Problems OR Quality of Life OR Oral Health Related Quality in Title Abstract Keyword - (Word variations have been searched)

SCOPUS- Number of results: 698

cleft AND lip AND palate OR oral AND cleft OR cleft OR cleft AND palate OR facial AND cleft OR cleft AND lip OR cleft AND alveolus
