# Supplementary material for: Evidence Mapping and Quality Analysis of Systematic Reviews on Various Aspects Related to Cleft Lip and Palate
Source: J Clin Med. 2023 Sep 16;12(18):6002. doi: 10.3390/jcm12186002 (PMC10532364; doi:10.3390/jcm12186002)
Supplement: Supplementary file 1 [file jcm-12-06002-s001.zip › Annexure 3.pdf]

Annexure 3: List of excluded articles with reasons

1. **<u>EVIDENCE PYRAMID WAS USED FOR RISK OF BIAS ASSESSMENT (12 electronic search, 6 other resources, Total= 18)</u>**

1. Abbott MM, Meara JG. Nasoalveolar molding in cleft care: is it efficacious? Plast Reconstr Surg. 2012 Sep;130(3):659-666. doi: 10.1097/PRS.0b013e31825dc10a. PMID: 22929251.

2. Bedi G, Vyas KS, Chung MT, Morrison SD, Asaad M, Mardini S. Telemedicine in International Cleft Care: A Systematic Review. Cleft Palate Craniofac J. 2021 Dec;58(12):1547-1555. doi: 10.1177/1055665621989140. Epub 2021 Feb 4. PMID: 33535816.

3. Bookman LB, Melton KR, Pan BS, Bender PL, Chini BA, Greenberg JM, Saal HM, Taylor JA, Elluru RG. Neonates with tongue-based airway obstruction: a systematic review. Otolaryngol Head Neck Surg. 2012 Jan;146(1):8-18. doi: 10.1177/0194599811421598. Epub 2011 Sep 16. PMID: 21926259.

4. Cho MJ, Rohrich RJ. Level of Evidence on Platelet-rich Plasma in Plastic Surgery. Plast Reconstr Surg Glob Open. 2021 Apr 15;9(4):e3379. doi: 10.1097/GOX.0000000000003379. PMID: 33868870; PMCID: PMC8049385.

5. Duarte GA, Ramos RB, Cardoso MC. Feeding methods for children with cleft lip and/or palate: a systematic review. Braz J Otorhinolaryngol. 2016 Sep-Oct;82(5):602-9. doi: 10.1016/j.bjorl.2015.10.020. Epub 2016 Mar 2. PMID: 26997574.

6. Virani FR, Chua EC, Timbang MR, Hsieh TY, Senders CW. Three-Dimensional Printing in Cleft Care: A Systematic Review. Cleft Palate Craniofac J. 2021 May 7:10556656211013175. doi: 10.1177/10556656211013175. Epub ahead of print. PMID: 33960208.

7. Kaura AS, Srinivasa DR, Kasten SJ. Optimal Timing of Alveolar Cleft Bone Grafting for Maxillary Clefts in the Cleft Palate Population. J Craniofac Surg. 2018 Sep;29(6):1551-1557. doi: 10.1097/SCS.0000000000004680. PMID: 29916970.

8. Maillard S, Retrouvey JM, Ahmed MK, Taub PJ. Correlation between Nasoalveolar Molding and Surgical, Aesthetic, Functional and Socioeconomic Outcomes Following Primary Repair Surgery: a Systematic Review. J Oral Maxillofac Res. 2017 Sep 30;8(3):e2. doi: 10.5037/jomr.2017.8302. PMID: 29142654; PMCID: PMC5676312.

9. Neumann S, Romonath R. Effectiveness of nasopharyngoscopic biofeedback in clients with cleft palate speech: a systematic review. Logoped Phoniatr Vocol. 2012 Oct;37(3):95-106. doi: 10.3109/14015439.2011.638669. Epub 2011 Dec 7. PMID: 22145632.

10. Pan W, Wu C, Yang Z, Duan Z, Su Z, Wang P, Zheng Q, Li C. Secondary Alveolar Bone Grafting and Iliac Cancellous Bone Harvesting for Patients With Alveolar Cleft. J Craniofac Surg. 2016 Jun;27(4):883-91. doi: 10.1097/SCS.0000000000002603. PMID: 27244214.

11. Pereira V, Sell D, Tuomainen J. The impact of maxillary osteotomy on speech outcomes in cleft lip and palate: an evidence-based approach to evaluating the literature. Cleft Palate Craniofac J. 2013 Jan;50(1):25-39. doi: 10.1597/11-116. Epub 2011 Nov 21. PMID: 22103908.

12. Reddy RR, Gosla Reddy S, Vaidhyanathan A, Bergé SJ, Kuijpers-Jagtman AM. Maxillofacial growth and speech outcome after one-stage or two-stage palatoplasty in unilateral cleft lip and palate. A systematic review. J Craniomaxillofac Surg. 2017 Jun;45(6):995-1003. doi: 10.1016/j.jcms.2017.03.006. Epub 2017 Mar 22. PMID: 28427835.

13. Sinclair N, Gharb BB, Papay F, Rampazzo A. Soft Tissue Reconstruction in Patients With Hemifacial Microsomia: A Systematic Review of the Literature. J Craniofac Surg. 2019 May/Jun;30(3):879-887. doi: 10.1097/SCS.0000000000005320. PMID: 30817535.

14. Sitzman TJ, Coyne SM, Britto MT. The Burden of Care for Children With Unilateral Cleft Lip: A Systematic Review of Revision Surgery. Cleft Palate Craniofac J. 2016 Jul;53(4):84-94. doi: 10.1597/14-202. Epub 2015 Aug 6. PMID: 26247708.

15. Tache A, Mommaerts MY. The need for maxillary osteotomy after primary cleft surgery: A systematic review framing a retrospective study. J Craniomaxillofac Surg. 2020 Oct;48(10):919-927. doi: 10.1016/j.jcms.2020.07.005. Epub 2020 Jul 19. PMID: 32768251.

16. Uzel A, Alparslan ZN. Long-term effects of presurgical infant orthopedics in patients with cleft lip and palate: a systematic review. Cleft Palate Craniofac J. 2011 Sep;48(5):587-95. doi: 10.1597/10-008. Epub 2010 Sep 17. PMID: 20858135.

17. Wermker K, Jung S, Joos U, Kleinheinz J. Dental implants in cleft lip, alveolus, and palate patients: a systematic review. Int J Oral Maxillofac Implants. 2014 Mar-Apr;29(2):384-90. doi: 10.11607/jomi.3303. PMID: 24683564.

18. Wu C, Pan W, Feng C, Su Z, Duan Z, Zheng Q, Hua C, Li C. Grafting materials for alveolar cleft reconstruction: a systematic review and best-evidence synthesis. Int J Oral Maxillofac Surg. 2018 Mar;47(3):345-356. doi: 10.1016/j.ijom.2017.08.003. Epub 2017 Aug 31. PMID: 28863859.

2. QUALITY ANALYSIS OR RISK OF BIAS ASSESSMENT OF INCLUDED STUDIES NOT PERFORMED (46-electronic search, 20-other resources, Total=66)

1. Abbas DB, Lavin C, Fahy EJ, Choo H, Truong MT, Bruckman KC, Khosla RK, Lorenz HP, Momeni A, Wan DC. A Systematic Review of Mandibular Distraction Osteogenesis Versus Orthodontic Airway Plate for Airway Obstruction Treatment in Pierre Robin Sequence. Cleft Palate Craniofac J. 2022 Mar;59(3):365-376. doi: 10.1177/10556656211011886. Epub 2021 Jun 2. PMID: 34075816.

2. Wahaj A, Hafeez K, Zafar MS. Role of bone graft materials for cleft lip and palate patients: A systematic review. The Saudi Journal for Dental Research. 2016 Jan 1;7(1):57-63.

3. Suzuki A, Yoshioka H, Summakia D, Desai NG, Jun G, Jia P, Loose DS, Ogata K, Gajera MV, Zhao Z, Iwata J. MicroRNA-124-3p suppresses mouse lip mesenchymal cell proliferation through the regulation of genes associated with cleft lip in the mouse. BMC Genomics. 2019 Nov 14;20(1):852. doi: 10.1186/s12864-019-6238-4. PMID: 31727022; PMCID: PMC6854646.

4. Alanko OM, Svedström-Oristo AL, Tuomisto MT. Patients' perceptions of orthognathic treatment, well-being, and psychological or psychiatric status: a systematic review. Acta Odontol Scand. 2010 Sep;68(5):249-60. doi: 10.3109/00016357.2010.494618. PMID: 20513168.

5. Al-Namankany A, Alhubaishi A. Effects of cleft lip and palate on children's psychological health: A systematic review. J Taibah Univ Med Sci. 2018 Jun 5;13(4):311-318. doi: 10.1016/j.jtumed.2018.04.007. PMID: 31435341; PMCID: PMC6694901.

6. Amooee A, Dastgheib SA, Niktabar SM, Noorishadkam M, Lookzadeh MH, Mirjalili SR, Heiranizadeh N, Neamatzadeh H. Association of Fetal MTHFR 677C > T Polymorphism with Non-Syndromic Cleft Lip with or without Palate Risk: A Systematic Review and Meta-Analysis. Fetal Pediatr Pathol. 2021 Aug;40(4):337-353. doi: 10.1080/15513815.2019.1707918. Epub 2019 Dec 27. PMID: 31880477.

7. Asaad M, Dey JK, Al-Mouakeh A, Manjouna MB, Nashed MA, Rajesh A, Boonipat T, Bite U. Eye-Tracking Technology in Plastic and Reconstructive Surgery: A Systematic Review. Aesthet Surg J. 2020 Aug 14;40(9):1022-1034. doi: 10.1093/asj/sjz328. PMID: 32010929.

8. Barbosa TS, Gavião MB. Oral health-related quality of life in children: part II. Effects of clinical oral health status. A systematic review. Int J Dent Hyg. 2008 May;6(2):100-7. doi: 10.1111/j.1601-5037.2008.00293.x. PMID: 18412721.

9. Batista LR, Triches TC, Moreira EA. Desenvolvimento bucal e aleitamento materno em crianças com fissura labiopalatal. Revista Paulista de Pediatria. 2011;29:674-9.

10. Bell R, Cowan K, Marston AP. Metanalysis of alloplastic materials versus autologous fat for injection augmentation pharyngoplasty treatment of velopharyngeal insufficiency. Int J Pediatr Otorhinolaryngol. 2021 Jul;146:110738. doi: 10.1016/j.ijporl.2021.110738. Epub 2021 Apr 28. PMID: 33957548.

11. Bittermann GK, de Ruiter AP, Janssen NG, Bittermann AJ, van der Molen AM, van Es RJ, Rosenberg AJ, Koole R. Management of the premaxilla in the treatment of bilateral cleft of lip and palate: what can the literature tell us? Clin Oral Investig. 2016 Mar;20(2):207-17. doi: 10.1007/s00784-015-1589-y. Epub 2015 Sep 16. PMID: 26374747; PMCID: PMC4762918.

12. Blessmann Weber JB, de Macedo Menezes L, Azeredo F, Lessa Filho LS. Volumetric assessment of alveolar clefts: a literature review. *J Oral Pathol Med*. 2017;46(8):569-573. doi:10.1111/jop.12548

13. Cabanillas-Aquino AG, Rojas-Yauri MC, Atoche-Socola KJ, Arriola-Guillén LE. Assessment of craniofacial and dental characteristics in individuals with treacher collins syndrome. A review. J Stomatol Oral Maxillofac Surg. 2021 Nov;122(5):511-515. doi: 10.1016/j.jormas.2020.10.011. Epub 2020 Nov 7. PMID: 33166690.

14. Cárdenas-Nieto D, Forero-Castro M, Esteban-Pérez C, Martínez-Lozano J, Briceño-Balcázar I. The 22q11.2 Microdeletion in Pediatric Patients with Cleft Lip, Palate, or Both and Congenital Heart Disease: A Systematic Review. J Pediatr Genet. 2020 Mar;9(1):1-8. doi: 10.1055/s-0039-1698804. Epub 2019 Oct 23. PMID: 31976137; PMCID: PMC6976326.

15. Chang IA, Bassiri Gharb B, Papay FA, Rampazzo A. Repair Sequence and Management of the Premaxilla in Patients With Bilateral Cleft Lip and Palate: A Systematic Review of the Literature. J Craniofac Surg. 2021 Sep 23. doi: 10.1097/SCS.0000000000008184. Epub ahead of print. PMID: 34560740.

16. de Ladeira PR, Alonso N. Protocols in cleft lip and palate treatment: systematic review. Plast Surg Int. 2012;2012:562892. doi: 10.1155/2012/562892. Epub 2012 Nov 1. PMID: 23213503; PMCID: PMC3503280.

17. Elhaddaoui R, Bahije L, Zaoui F, Rerhrhaye W. Calendrier de la greffe osseuse et séquences d'éruption canine dans les cas de fentes labio-alvéolo-palatines: revue systématique. Orthodontie Française. 2017 Jun 1;88(2).

18. Forna F, McConnell M, Kitabire FN, Homsy J, Brooks JT, Mermin J, Weidle PJ. Systematic review of the safety of trimethoprim-sulfamethoxazole for prophylaxis in HIV-infected pregnant women: implications for resource-limited settings. AIDS Rev. 2006 Jan-Mar;8(1):24-36. PMID: 16736949.

19. Goldberg O, Moretti M, Levy A, Koren G. Exposure to nitrofurantoin during early pregnancy and congenital malformations: a systematic review and meta-analysis. J Obstet Gynaecol Can. 2015 Feb;37(2):150-156. doi: 10.1016/S1701-2163(15)30337-6. PMID: 25767948.

20. Grimes CE, Henry JA, Maraka J, Mkandawire NC, Cotton M. Cost-effectiveness of surgery in low- and middle-income countries: a systematic review. World J Surg. 2014 Jan;38(1):252-63. doi: 10.1007/s00268-013-2243-y. PMID: 24101020.

21. Grover R, Barnett A, Rampazzo A, Papay F, Gharb BB. Outcomes of Sphincter Pharyngoplasty in the Cleft Population. J Craniofac Surg. 2018 Jun;29(4):908-913. doi: 10.1097/SCS.0000000000004289. PMID: 29750723.

22. Hassan T, Naini FB, Gill DS. The effects of orthognathic surgery on speech: a review. J Oral Maxillofac Surg. 2007 Dec;65(12):2536-43. doi: 10.1016/j.joms.2007.05.018. PMID: 18022481.

23. Lancaster HS, Lien KM, Haas J, Ellis P, Scherer NJ. Reading Development in Children With Nonsyndromic Cleft Palate With or Without Cleft Lip: Meta-analysis and Systematic Review. Cleft Palate Craniofac J. 2021 Sep 13:10556656211039871. doi: 10.1177/10556656211039871. Epub ahead of print. PMID: 34516236.

24. Hunt O, Burden D, Hepper P, Johnston C. The psychosocial effects of cleft lip and palate: a systematic review. Eur J Orthod. 2005 Jun;27(3):274-85. doi: 10.1093/ejo/cji004. PMID: 15947228.

25. Janssen NG, Weijs WL, Koole R, Rosenberg AJ, Meijer GJ. Tissue engineering strategies for alveolar cleft reconstruction: a systematic review of the literature. Clin Oral Investig. 2014 Jan;18(1):219-26. doi: 10.1007/s00784-013-0947-x. Epub 2013 Feb 22. PMID: 23430342.

26. Jentink J, Dolk H, Loane MA, Morris JK, Wellesley D, Garne E, de Jong-van den Berg L; EUROCAT Antiepileptic Study Working Group. Intrauterine exposure to carbamazepine and specific congenital malformations: systematic review and case-control study. BMJ. 2010 Dec 2;341:c6581. doi: 10.1136/bmj.c6581. PMID: 21127116; PMCID: PMC2996546.

27. Jodeh DS, Buller M, Rottgers SA. The Impact of Presurgical Infant Orthopedics on Oronasal Fistula Rates Following Cleft Repair: A Meta-Analysis. Cleft Palate Craniofac J. 2019 May;56(5):576-585. doi: 10.1177/1055665618806104. Epub 2018 Oct 31. PMID: 30379094.

28. Johnson CY, Little J. Folate intake, markers of folate status and oral clefts: is the evidence converging? Int J Epidemiol. 2008 Oct;37(5):1041-58. doi: 10.1093/ije/dyn098. Epub 2008 Jun 26. PMID: 18583393.

29. Kanatas AN, Rogers SN. A systematic review of patient self-completed questionnaires suitable for oral and maxillofacial surgery. Br J Oral Maxillofac Surg. 2010 Dec;48(8):579-90. doi: 10.1016/j.bjoms.2009.12.004. Epub 2009 Dec 31. PMID: 20045231.

30. Klassen AF, Tsangaris E, Forrest CR, Wong KW, Pusic AL, Cano SJ, Syed I, Dua M, Kainth S, Johnson J, Goodacre T. Quality of life of children treated for cleft lip and/or palate: a systematic review. J Plast Reconstr Aesthet Surg. 2012 May;65(5):547-57. doi: 10.1016/j.bjps.2011.11.004. Epub 2011 Nov 25. PMID: 22118856.

31. Lancaster HS, Lien KM, Chow JC, Frey JR, Scherer NJ, Kaiser AP. Early Speech and Language Development in Children With Nonsyndromic Cleft Lip and/or Palate: A Meta-Analysis. J Speech Lang Hear Res. 2019 Dec 13;63(1):14-31. doi: 10.1044/2019_JSLHR-19-00162. Erratum in: J Speech Lang Hear Res. 2020 Aug 10;63(8):2731. PMID: 31841365; PMCID: PMC7213476.

32. Paniagua LM, Signorini AV, Costa SS, Collares MV, Dornelles S. Velopharyngeal dysfunction: a systematic review of major instrumental and auditory-perceptual assessments. Int Arch Otorhinolaryngol. 2013 Jul;17(3):251-6. doi: 10.7162/S1809-97772013000300004. PMID: 25992022; PMCID: PMC4423245.

33. Li YH, Yang J, Zhang JL, Liu JQ, Zheng Z, Hu DH. BMP4 rs17563 polymorphism and nonsyndromic cleft lip with or without cleft palate: A meta-analysis. Medicine (Baltimore). 2017 Aug;96(31):e7676. doi: 10.1097/MD.0000000000007676. PMID: 28767592; PMCID: PMC5626146.

34. Shrime MG, Sleemi A, Ravilla TD. Charitable platforms in global surgery: a systematic review of their effectiveness, cost-effectiveness, sustainability, and role training. World J Surg. 2015 Jan;39(1):10-20. doi: 10.1007/s00268-014-2516-0. PMID: 24682278; PMCID: PMC4179995.

35. McGrattan K, Ellis C. Team-oriented care for orofacial clefts: a review of the literature. Cleft Palate Craniofac J. 2013 Jan;50(1):13-8. doi: 10.1597/11-137. Epub 2011 Oct 24. PMID: 22023220.

36. McKinney CM, Glass RP, Coffey P, Rue T, Vaughn MG, Cunningham M. Feeding Neonates by Cup: A Systematic Review of the Literature. Matern Child Health J. 2016 Aug;20(8):1620-33. doi: 10.1007/s10995-016-1961-9. PMID: 27016350; PMCID: PMC5091933.

37. Montagnoli C, Santoro CB, Buzzi T, Bortolus R. Maternal periconceptional nutrition matters. A scoping review of the current literature. J Matern Fetal Neonatal Med. 2021 Aug 15:1-18. doi: 10.1080/14767058.2021.1962843. Epub ahead of print. PMID: 34392783.

38. Mota-Rodríguez AN, Olmedo-Hernández O, Argueta-Figueroa L. A systematic analysis of evidence for surgically accelerated orthodontics. J Clin Exp Dent. 2019 Sep 1;11(9):e829-e838. doi: 10.4317/jced.56048. PMID: 31636876; PMCID: PMC6797450.

39. Timbang MR, Gharb BB, Rampazzo A, Papay F, Zins J, Doumit G. A systematic review comparing Furlow double-opposing Z-plasty and straight-line intravelar veloplasty methods of cleft palate repair. Plastic and Reconstructive Surgery. 2014 Nov 1;134(5):1014-22.

40. Nigh E, Rubio GA, Hillam J, Armstrong M, Debs L, Thaller SR. Autologous fat injection for treatment of velopharyngeal insufficiency. Journal of Craniofacial Surgery. 2017 Jul 1;28(5):1248-54.

41. Nollet PJ, Katsaros C, Van't Hof MA, Kuijpers-Jagtman AM. Treatment outcome in unilateral cleft lip and palate evaluated with the GOSLON yardstick: a meta-analysis of 1236 patients. Plast Reconstr Surg. 2005 Oct;116(5):1255-62. doi: 10.1097/01.prs.0000181652.84855.a3. PMID: 16217465.

42. Palomares-Aguilera M, Inostroza-Allende F, Solar LR. Speech pathology telepractice intervention during the COVID-19 pandemic for Spanish-speaking children with cleft palate: A systematic review. Int J Pediatr Otorhinolaryngol. 2021 May;144:110700. doi: 10.1016/j.ijporl.2021.110700. Epub 2021 Apr 2. PMID: 33862334; PMCID: PMC8016537.

43. Pfaff MJ, Musavi L, Wang MM, Haveles CS, Liu C, Rezzadeh KS, Lee JC. Oral Flora and Perioperative Antimicrobial Interventions in Cleft Palate Surgery: A Review of the Literature. Cleft Palate Craniofac J. 2021 Aug;58(8):990-998. doi: 10.1177/1055665620977363. Epub 2020 Dec 11. PMID: 33302728.

44. Phan M, Conte F, Khandelwal KD, Ockeloen CW, Bartzela T, Kleefstra T, van Bokhoven H, Rubini M, Zhou H, Carels CE. Tooth agenesis and orofacial clefting: genetic brothers in arms? Hum Genet. 2016 Dec;135(12):1299-1327. doi: 10.1007/s00439-016-1733-z. Epub 2016 Oct 3. PMID: 27699475; PMCID: PMC5065589.

45. Raveendran M. Simulation-Based Training Models for Cleft Palate Repair: A Systematic Review. Cleft Palate Craniofac J. 2020 Apr;57(4):506-511. doi: 10.1177/1055665619877768. Epub 2019 Sep 25. PMID: 31554411.

46. Reichert C, Götz W, Smeets R, Wenghöfer M, Jäger A. The impact of nonautogenous bone graft on orthodontic treatment. Quintessence Int. 2010 Sep;41(8):665-672. PMID: 20657856.

47. Chang IA, Bassiri Gharb B, Papay FA, Rampazzo A. Repair Sequence and Management of the Premaxilla in Patients With Bilateral Cleft Lip and Palate: A Systematic Review of the Literature. J Craniofac Surg. 2021 Sep 23. doi: 10.1097/SCS.0000000000008184. Epub ahead of print. PMID: 34560740.

48. Rodrigues R, Fernandes MH, Bessa Monteiro A, Furfuro R, Carvalho Silva C, Vardasca R, Mendes J, Manso MC. Are there any solutions for improving the cleft area hygiene in patients with cleft lip and palate? A systematic review. Int J Dent Hyg. 2019 May;17(2):130-141. doi: 10.1111/idh.12385. Epub 2019 Feb 22. PMID: 30697916.

49. Sabbagh HJ, Mossey PA, Innes NP. Prevalence of orofacial clefts in Saudi Arabia and neighboring countries: A systematic review. Saudi Dent J. 2012 Jan;24(1):3-10. doi: 10.1016/j.sdentj.2011.11.001. Epub 2011 Dec 2. PMID: 23960521; PMCID: PMC3723074.

50. Saha A, Shah S, Waknis P, Bhujbal P, Aher S, Vaswani V. Comparison of minimally invasive versus conventional open harvesting technique for iliac bone graft in secondary alveolar bone grafting in cleft palate patients: a systematic review. J Korean Assoc Oral Maxillofac Surg. 2019 Oct;45(5):241-253. doi: 10.5125/jkaoms.2019.45.5.241. Epub 2019 Oct 30. PMID: 31728331; PMCID: PMC6838348.

51. Scolozzi P. Distraction osteogenesis in the management of severe maxillary hypoplasia in cleft lip and palate patients. J Craniofac Surg. 2008 Sep;19(5):1199-214. doi: 10.1097/SCS.0b013e318184365d. PMID: 18812842.

52. Vella SL, Pai NB. A systematic review of the quality of life of carers of children with cleft lip and/or plate. 2012 (91)

53. Sharif F, Rehman IU, Muhammad N, MacNeil S. Dental materials for cleft palate repair. Materials Science and Engineering: C. 2016 Apr 1;61:1018-28.

54. Sharma VP, Bella H, Cadier MM, Pigott RW, Goodacre TE, Richard BM. Outcomes in facial aesthetics in cleft lip and palate surgery: a systematic review. J Plast Reconstr Aesthet Surg. 2012 Sep;65(9):1233-45. doi: 10.1016/j.bjps.2012.04.001. Epub 2012 May 15. Erratum in: J Plast Reconstr Aesthet Surg. 2012 Dec;65(12):1759. PMID: 22591614.

55. Shash H, Al-Halabi B, Jozaghi Y, Aldekhayel S, Gilardino MS. A Review of Tissue Expansion-Assisted Techniques of Cleft Palate Repair. J Craniofac Surg. 2016 May;27(3):760-6. doi: 10.1097/SCS.0000000000002468. PMID: 27035596.

56. Minić S, Trpinac D, Gabriel H, Gencik M, Obradović M. Dental and oral anomalies in incontinentia pigmenti: a systematic review. Clin Oral Investig. 2013 Jan;17(1):1-8. doi: 10.1007/s00784-012-0721-5. Epub 2012 Mar 28. PMID: 22453515.

57. Sreejith VP, Arun V, Devarajan AP, Gopinath A, Sunil M. Psychological Effect of Prenatal Diagnosis of Cleft Lip and Palate: A Systematic Review. Contemp Clin Dent. 2018 Apr-Jun;9(2):304-308. doi: 10.4103/ccd.ccd_673_17. PMID: 29875578; PMCID: PMC5968700.

58. Stewart BT, Hatcher KW, Sengupta A, Burg RV. Cleft-Related Infanticide and Abandonment: A Systematic Review of the Academic and Lay Literature. Cleft Palate Craniofac J. 2018 Jan;55(1):98-104. doi: 10.1177/1055665617721919. Epub 2017 Dec 14. PMID: 34162058.

59. Stothard KJ, Tennant PW, Bell R, Rankin J. Maternal overweight and obesity and the risk of congenital anomalies: a systematic review and meta-analysis. JAMA. 2009 Feb 11;301(6):636-50. doi: 10.1001/jama.2009.113. PMID: 19211471.

60. Tapia VJ, Epstein S, Tolmach OS, Hassan AS, Chung NN, Gosman AA. Health-Related Quality-of-Life Instruments for Pediatric Patients with Diverse Facial Deformities: A Systematic Literature Review. Plast Reconstr Surg. 2016 Jul;138(1):175-187. doi: 10.1097/PRS.0000000000002285. PMID: 27348649.

61. Timbang MR, Gharb BB, Rampazzo A, Papay F, Zins J, Doumit G. A systematic review comparing Furlow double-opposing Z-plasty and straight-line intravelar veloplasty methods of cleft palate repair. Plastic and Reconstructive Surgery. 2014 Nov 1;134(5):1014-22.

62. Trabut S, Friedrich H, Caversaccio M, Negoias S. Challenges in topical therapy of chronic rhinosinusitis: The case of nasal drops application - A systematic review. Auris Nasus Larynx. 2020 Aug;47(4):536-543. doi: 10.1016/j.anl.2020.05.026. Epub 2020 Jul 1. PMID: 32620293.

63. van Hout WM, Mink van der Molen AB, Breugem CC, Koole R, Van Cann EM. Reconstruction of the alveolar cleft: can growth factor-aided tissue engineering replace autologous bone grafting? A literature review and systematic review of results obtained with bone morphogenetic protein-2. Clin Oral Investig. 2011 Jun;15(3):297-303. doi: 10.1007/s00784-011-0547-6. Epub 2011 Apr 5. PMID: 21465220; PMCID: PMC3089722.

64. Vyas KS, Hambrick HR, Shakir A, Morrison SD, Tran DC, Pearson K, Vasconez HC, Mardini S, Gosman AA, Dobke M, Granick MS. A Systematic Review of the Use of Telemedicine in Plastic and Reconstructive Surgery and Dermatology. Ann Plast Surg. 2017 Jun;78(6):736-768. doi: 10.1097/SAP.0000000000001044. PMID: 28328635.

65. Wester JR, Weissman JP, Reddy NK, Chwa ES, Gosain AK. The Current State of Cleft Care in Sub-Saharan Africa: A Narrative Review. Cleft Palate Craniofac J. 2021 Aug 16:10556656211038183. doi: 10.1177/10556656211038183. Epub ahead of print. PMID: 34397305.

66. Yamaguchi K, Lonic D, Lo LJ. Complications following orthognathic surgery for patients with cleft lip/palate: A systematic review. J Formos Med Assoc. 2016 Apr;115(4):269-77. doi: 10.1016/j.jfma.2015.10.009. Epub 2015 Dec 11. PMID: 26686426.

3. LETTER TO EDITOR AND BRIEF COMMUNICATION (Electronic search= 3)

1. Liu Y, Hua F, He H. Nasoalveolar molding therapy may offer positive effects on unilateral clefts of lip and/or palate. Journal of Evidence Based Dental Practice. 2018 Sep 1;18(3):252-4.

2. Ludwig KU, Mangold E, Herms S, Nowak S, Reutter H, Paul A, Becker J, Herberz R, AlChawa T, Nasser E, Böhmer AC, Mattheisen M, Alblas MA, Barth S, Kluck N, Lauster C, Braumann B, Reich RH, Hemprich A, Pötzsch S, Blaumeiser B, Daratsianos N, Kreusch T, Murray JC, Marazita ML, Ruczinski I, Scott AF, Beaty TH, Kramer FJ, Wienker TF, Steegers-Theunissen RP, Rubini M, Mossey PA, Hoffmann P, Lange C, Cichon S, Propping P, Knapp M, Nöthen MM. Genome-wide meta-analyses of nonsyndromic cleft lip with or without cleft palate identify six new risk loci. Nat Genet. 2012 Sep;44(9):968-71. doi: 10.1038/ng.2360. Epub 2012 Aug 5. PMID: 22863734; PMCID: PMC3598617.

3. Nardini G, Flores RL. A systematic review comparing furlow double-opposing z-plasty and straight-line intravelar veloplasty methods of cleft palate repair. *Plast Reconstr Surg*. 2015;135(5):927e-928e. doi:10.1097/PRS.0000000000001185

4. **SINGLE DATA BASE SEARCH DONE (Total=34)**

1. Hackshaw A, Rodeck C, Boniface S. Maternal smoking in pregnancy and birth defects: a systematic review based on 173 687 malformed cases and 11.7 million controls. Hum Reprod Update. 2011 Sep-Oct;17(5):589-604. doi: 10.1093/humupd/dmr022. Epub 2011 Jul 11. PMID: 21747128; PMCID: PMC3156888.

2. Khojasteh A, Kheiri L, Motamedian SR, Nadjmi N. Regenerative medicine in the treatment of alveolar cleft defect: A systematic review of the literature. J Craniomaxillofac Surg. 2015 Oct;43(8):1608-13. doi: 10.1016/j.jcms.2015.06.041. Epub 2015 Jul 26. PMID: 26302939.

3. Bartkowska P, Komisarek O. Scar management in patients after cleft lip repair-Systematic review Cleft lip scar management. J Cosmet Dermatol. 2020 Aug;19(8):1866-1876. doi: 10.1111/jocd.13511. Epub 2020 Jun 9. PMID: 32458543

4. Bykowski MR, Naran S, Winger DG, Losee JE. The Rate of Oronasal Fistula Following Primary Cleft Palate Surgery: A Meta-Analysis. Cleft Palate Craniofac J. 2015 Jul;52(4):e81-7. doi: 10.1597/14-127. Epub 2014 Oct 16. PMID: 25322441

5. Chao TE, Sharma K, Mandigo M, Hagander L, Resch SC, Weiser TG, Meara JG. Cost-effectiveness of surgery and its policy implications for global health: a systematic review and analysis. Lancet Glob Health. 2014 Jun;2(6):e334-45. doi: 10.1016/S2214-109X(14)70213-X. Epub 2014 May 21. PMID: 25103302

6. Cheung LK, Chua HD. A meta-analysis of cleft maxillary osteotomy and distraction osteogenesis. Int J Oral Maxillofac Surg. 2006 Jan;35(1):14-24. doi: 10.1016/j.ijom.2005.06.008. Epub 2005 Sep 8. PMID: 16154316.

7. Chiu YT, Liao YF. Is cleft severity related to maxillary growth in patients with unilateral cleft lip and palate? Cleft Palate Craniofac J. 2012 Sep;49(5):535-40. doi: 10.1597/10-044. Epub 2010 Dec 23. PMID: 21214323.

8. De Grauwe A, Ayaz I, Shujaat S, Dimitrov S, Gbadegbegnon L, Vande Vannet B, Jacobs R. CBCT in orthodontics: a systematic review on justification of CBCT in a paediatric population prior to orthodontic treatment. Eur J Orthod. 2019 Aug 8;41(4):381-389. doi: 10.1093/ejo/cjy066. PMID: 30351398; PMCID: PMC6686083.

9. Chen EK, Zmirou-Navier D, Padilla C, Deguen S. Effects of air pollution on the risk of congenital anomalies: a systematic review and meta-analysis. Int J Environ Res Public Health. 2014 Jul 31;11(8):7642-68. doi: 10.3390/ijerph110807642. PMID: 25089772; PMCID: PMC4143824.

10. Gupta A, Svider PF, Rayess H, Sheyn A, Folbe AJ, Eloy JA, Zuliani G, Carron MA. Pediatric rhinoplasty: A discussion of perioperative considerations and systematic review. Int J Pediatr Otorhinolaryngol. 2017 Jan;92:11-16. doi: 10.1016/j.ijporl.2016.10.027. Epub 2016 Oct 26. PMID: 28012510.

11. Hasslöf P, Twetman S. Caries prevalence in children with cleft lip and palate--a systematic review of case-control studies. Int J Paediatr Dent. 2007 Sep;17(5):313-9. doi: 10.1111/j.1365-263X.2007.00847.x. PMID: 17683319.

12. Heidsieck DS, Smarius BJ, Oomen KP, Breugem CC. The role of the tensor veli palatini muscle in the development of cleft palate-associated middle ear problems. Clin Oral Investig. 2016 Sep;20(7):1389-401. doi: 10.1007/s00784-016-1828-x. Epub 2016 May 7. PMID: 27153847; PMCID: PMC4992026.

13. Kantar RS, Rifkin WJ, Cammarata MJ, Maliha SG, Diaz-Siso JR, Farber SJ, Flores RL. Single-Stage Primary Cleft Lip and Palate Repair: A Review of the Literature. Ann Plast Surg. 2018 Nov;81(5):619-623. doi: 10.1097/SAP.0000000000001543. PMID: 29944528.

14. Khojasteh A, Kheiri L, Motamedian SR, Nadjmi N. Regenerative medicine in the treatment of alveolar cleft defect: A systematic review of the literature. J Craniomaxillofac Surg. 2015 Oct;43(8):1608-13. doi: 10.1016/j.jcms.2015.06.041. Epub 2015 Jul 26. PMID: 26302939.

15. Kim H, Hwang K, Yun SM. Catgut and its Use in Plastic Surgery. J Craniofac Surg. 2020 May/Jun;31(3):876-878. doi: 10.1097/SCS.0000000000006149. PMID: 32195830.

16. Lee YH, Liao YF. Hard palate-repair technique and facial growth in patients with cleft lip and palate: a systematic review. Br J Oral Maxillofac Surg. 2013 Dec;51(8):851-7. doi: 10.1016/j.bjoms.2013.08.012. Epub 2013 Sep 14. PMID: 24045106.

17. Liao YF, Mars M. Hard palate repair timing and facial growth in cleft lip and palate: a systematic review. Cleft Palate Craniofac J. 2006 Sep;43(5):563-70. doi: 10.1597/05-058. PMID: 16986982.

18. Mulyadi M, Kreshanti P, Handayani S, Bangun K. Maxillary growth and speech outcome in patient with cleft lip and palate after two-stage palate repair: a systematic review. Jurnal Plastik Rekonstruksi. 2012;1(4).

19. Munabi NCO, Swanson J, Auslander A, Sanchez-Lara PA, Davidson Ward SL, Magee WP 3rd. The Prevalence of Congenital Heart Disease in Nonsyndromic Cleft Lip and/or Palate: A Systematic Review of the Literature. Ann Plast Surg. 2017 Aug;79(2):214-220. doi: 10.1097/SAP.0000000000001069. PMID: 28570447.

20. Panamonta V, Pradubwong S, Panamonta M, Chowchuen B. Prevalence of Congenital Heart Diseases in Patients with Orofacial Clefts: A Systematic Review. J Med Assoc Thai. 2015 Aug;98 Suppl 7:S22-7. PMID: 26742365.

21. Panamonta V, Pradubwong S, Panamonta M, Chowchuen B. Global Birth Prevalence of Orofacial Clefts: A Systematic Review. J Med Assoc Thai. 2015 Aug;98 Suppl 7:S11-21. PMID: 26742364.

22. Panamonta V, Wichajarn K, Wongswadiwat Y, Panamonta M, Pradubwong S, Chowchuen B. Assessment of Chromosome 22q11.2 Deletion in Patients with Isolated Cleft Palate: A Systematic Review of Prospective Studies. J Med Assoc Thai. 2016 Aug;99 Suppl 5:S194-8. PMID: 29906081.

23. Price KE, Haddad Y, Fakhouri WD. Analysis of the Relationship Between Micrognathia and Cleft Palate: A Systematic Review. Cleft Palate Craniofac J. 2016 Mar;53(2):e34-44. doi: 10.1597/14-238. Epub 2015 Feb 6. PMID: 25658963.

24. Ranzer M, Daniele E, Purnell CA. Perioperative Management of Cleft Lip Repair: A Meta-Analysis and Clinical Practice Guideline. Cleft Palate Craniofac J. 2021 Oct;58(10):1217-1225. doi: 10.1177/1055665620984909. Epub 2021 Jan 5. PMID: 33401938.

25. Stasiak M, Wojtaszek-Słomińska A, Racka-Pilszak B. Current methods for secondary alveolar bone grafting assessment in cleft lip and palate patients - A systematic review. J Craniomaxillofac Surg. 2019 Apr;47(4):578-585. doi: 10.1016/j.jcms.2019.01.013. Epub 2019 Jan 17. PMID: 30733132.

26. Choi KJ, Wlodarczyk JR, Nagengast ES, Wolfswinkel E, Munabi NCO, Yao C, Magee WP 3rd. The Likelihood of Orthognathic Surgery After Orofacial Cleft Repair. J Craniofac Surg. 2021 May 1;32(3):902-906. doi: 10.1097/SCS.0000000000007262. PMID: 33235169.

27. van den Bosch S, Koudstaal M, Versnel S, Maal T, Xi T, Nelen W, Bergé S, Faber M. Patients and professionals have different views on online patient information about cleft lip and palate (CL/P). Int J Oral Maxillofac Surg. 2016 Jun;45(6):692-9. doi: 10.1016/j.ijom.2015.11.018. Epub 2015 Dec 21. PMID: 26718136.

28. Wang F, Wu Y, Zou D, Wang G, Kaigler D. Clinical outcomes of dental implant therapy in alveolar cleft patients: a systematic review. Int J Oral Maxillofac Implants. 2014 Sep-Oct;29(5):1098-105. doi: 10.11607/jomi.3585. PMID: 25216135.

29. Maarse W, Rozendaal AM, Pajkrt E, Vermeij-Keers C, Mink van der Molen AB, van den Boogaard MJ. A systematic review of associated structural and chromosomal defects in oral clefts: when is prenatal genetic analysis indicated? J Med Genet. 2012 Aug;49(8):490-8. doi: 10.1136/jmedgenet-2012-101013. PMID: 22889852.

30. Wlodarczyk JR, Munabi NCO, Wolfswinkel E, Nagengast E, Higuchi EC, Turk M, Urata MM, Hammoudeh JA, Yao C, Magee W 3rd. Midface Growth Potential in Unoperated Clefts: A Systematic Review and Meta-Analysis. J Craniofac Surg. 2021 Oct 21. doi: 10.1097/SCS.0000000000008296. Epub ahead of print. PMID: 34690318.

31. Wlodarczyk JR, Brannon B, Munabi NCO, Wolfswinkel EM, Nagengast ES, Yao CA, Magee W 3rd. A Meta-Analysis of Palatal Repair Timing. J Craniofac Surg. 2021 Mar-Apr 01;32(2):647-651. doi: 10.1097/SCS.0000000000007029. PMID: 33705001.

32. Yang IY, Liao YF. The effect of 1-stage versus 2-stage palate repair on facial growth in patients with cleft lip and palate: a review. Int J Oral Maxillofac Surg. 2010 Oct;39(10):945-50. doi: 10.1016/j.ijom.2010.04.053. Epub 2010 Jul 15. PMID: 20637567

33. Missier MS, Jain RK, Kumar MP. Nasoalveolar molding and their effects on the dentoalveolar arch: A systematic review. Drug Invention Today. 2019 Jan 1;11(1).

34. Azadgoli B, Munabi NC, Fahradyan A, Auslander A, McCullough M, Aflatooni N, Davidson Ward SL, Kumar SR, Sanchez-Lara PA, Swanson J, Magee III WP. Congenital heart disease in patients with cleft lip/palate and its impact on cleft management. The Cleft Palate-Craniofacial Journal. 2020 Aug;57(8):957-66.

5. PROTOCOL OF SYSTEMATIC REVIEWS (TOTAL=2)

1. Forsetlund L, Semb G, Farah MG, Flottorp S. The Scientific Knowledge Base for Treatment of Patients with Cleft Lip, Alveolus and Palate [Internet]. Oslo, Norway: Knowledge Centre for the Health Services at The Norwegian Institute of Public Health (NIPH); 2009 Oct. Report from Norwegian Knowledge Centre for the Health Services (NOKC) No. 29-2009. PMID: 29320130.

2. Ravlyk L, Alawami S, Al-Jewair T. Secondary Alveolar Bone Graft Using Recombinant Human Bone Morphogenetic Protein-2 (rh-BMP2) May Be as Efficacious as Autologous Grafting in Unilateral Cleft Lip and Palate Patients. J Evid Based Dent Pract. 2020 Jun;20(2):101417. doi: 10.1016/j.jebdp.2020.101417. Epub 2020 Feb 19. PMID: 32473802.

6. RISK OF BIAS ASSESSMENT OF INLCUDED STUDIES NOT DETAILED (11-electronic search, 7-other sources, Total=18)

1. Aljohani M, Yates J, Ashley M, O'Malley L. Evaluation of Quality of Life in Adult Individuals With Cleft Lip and/or Palate. J Craniofac Surg. 2021 Mar-Apr 01;32(2):505-508. doi: 10.1097/SCS.0000000000007071. PMID: 33704970.

2. Collins J, Cheung K, Farrokhyar F, Strumas N. Pharyngeal flap versus sphincter pharyngoplasty for the treatment of velopharyngeal insufficiency: a meta-analysis. J Plast Reconstr Aesthet Surg. 2012 Jul;65(7):864-8. doi: 10.1016/j.bjps.2012.01.020. Epub 2012 Mar 28. PMID: 22459796.

3. Fan D, Wu S, Liu L, Xia Q, Tian G, Wang W, Ye S, Wang L, Rao J, Yang X, Yu Z, Xin L, Li S, Duan Z, Zhang T, Wu S, Guo X, Liu Z. Prevalence of non-syndromic orofacial clefts: based on 15,094,978 Chinese perinatal infants. Oncotarget. 2018 Jan 13;9(17):13981-13990. doi: 10.18632/oncotarget.24238. PMID: 29568410; PMCID: PMC5862631.

4. Gallagher ER, Collett BR. Neurodevelopmental and Academic Outcomes in Children With Orofacial Clefts: A Systematic Review. Pediatrics. 2019 Jul;144(1):e20184027. doi: 10.1542/peds.2018-4027. Epub 2019 Jun 12. PMID: 31189616.

5. Garland K, McNeely B, Dubois L, Matic D. Systematic Review of the Long-Term Effects of Presurgical Orthopedic Devices on Patient Outcomes. Cleft Palate Craniofac J. 2022 Feb;59(2):156-165. doi: 10.1177/1055665621998176. Epub 2021 Mar 8. PMID: 33678051; PMCID: PMC8750140.

6. Lee TH, Liu TT, Sung CW, Ou JC, Yeh JJ, Kuo YT, Wang YH. Association between IRF6 rs642961 polymorphism and non-syndromic cleft lip with or without a cleft palate: a systematic review and meta-analysis. Int. J. Clin. Exp. Med. 2018 Jan 1;11:3033-42.

7. Verstraaten J, Kuijpers-Jagtman AM, Mommaerts MY, Bergé SJ, Nada RM, Schols JG; Eurocran Distraction Osteogenesis Group. A systematic review of the effects of bone-borne surgical assisted rapid maxillary

expansion. J Craniomaxillofac Surg. 2010 Apr;38(3):166-74. doi: 10.1016/j.jcms.2009.06.006. Epub 2009 Aug 6. PMID: 19664932.

8. Maarse W, Bergé SJ, Pistorius L, van Barneveld T, Kon M, Breugem C, Mink van der Molen AB. Diagnostic accuracy of transabdominal ultrasound in detecting prenatal cleft lip and palate: a systematic review. Ultrasound Obstet Gynecol. 2010 Apr;35(4):495-502. doi: 10.1002/uog.7472. PMID: 20235140.

9. Matsunaka E, Ueki S, Makimoto K. Impact of breastfeeding or bottle-feeding on surgical wound dehiscence after cleft lip repair in infants: a systematic review protocol. JBI Database System Rev Implement Rep. 2015 Oct;13(10):3-11. doi: 10.11124/jbisrir-2015-2336. PMID: 26571277.

10. Gajera M, Desai N, Suzuki A, Li A, Zhang M, Jun G, Jia P, Zhao Z, Iwata J. MicroRNA-655-3p and microRNA-497-5p inhibit cell proliferation in cultured human lip cells through the regulation of genes related to human cleft lip. BMC Med Genomics. 2019 May 23;12(1):70. doi: 10.1186/s12920-019-0535-2. PMID: 31122291; PMCID: PMC6533741.

11. Mossey PA, Batra P, McIntyre GT. The parental dentocraniofacial phenotype-an orofacial clefting microform. Cleft Palate Craniofac J. 2010 Jan;47(1):22-34. doi: 10.1597/08-158.1. PMID: 20078200.

12. Murphy VE, Wang G, Namazy JA, Powell H, Gibson PG, Chambers C, Schatz M. The risk of congenital malformations, perinatal mortality and neonatal hospitalisation among pregnant women with asthma: a systematic review and meta-analysis. BJOG. 2013 Jun;120(7):812-22. doi: 10.1111/1471-0528.12224. Epub 2013 Mar 26. Erratum in: BJOG. 2014 Apr;121(5):652. PMID: 23530780.

13. Oberhofer HM, Breslin N, Heindel H, Ching J. Analgesic Efficacy of Intraoperative Nerve Blocks for Primary Palatoplasty. J Craniofac Surg. 2021 Mar-Apr 01;32(2):594-596. doi: 10.1097/SCS.0000000000006829. PMID: 33704988.

14. Papadopoulos MA, Koumpridou EN, Vakalis ML, Papageorgiou SN. Effectiveness of pre-surgical infant orthopedic treatment for cleft lip and palate patients: a systematic review and meta-analysis. Orthod Craniofac Res. 2012 Nov;15(4):207-36. doi: 10.1111/j.1601-6343.2012.01552.x. Epub 2012 Aug 16. PMID: 23020693.

15. Ranganathan K, Vercler CJ, Warschausky SA, MacEachern MP, Buchman SR, Waljee JF. Comparative effectiveness studies examining patient-reported outcomes among children with cleft lip and/or palate: a systematic review. Plast Reconstr Surg. 2015 Jan;135(1):198-211. doi: 10.1097/PRS.0000000000000825. PMID: 25539306.

16. Sharif MO, Callery P, Tierney S. The perspectives of children and young people living with cleft lip and palate: a review of qualitative literature. Cleft Palate Craniofac J. 2013 May;50(3):297-304. doi: 10.1597/12-054. Epub 2012 Oct 2. PMID: 23030697.

17. Ponduri S, Bradley R, Ellis PE, Brookes ST, Sandy JR, Ness AR. The management of otitis media with early routine insertion of grommets in children with cleft palate -- a systematic review. Cleft Palate Craniofac J. 2009 Jan;46(1):30-8. doi: 10.1597/07-219.1. Epub 2008 May 15. PMID: 19115800.

18. Wattanawong K, Rattanasiri S, McEvoy M, Attia J, Thakkinstian A. Association between IRF6 and 8q24 polymorphisms and nonsyndromic cleft lip with or without cleft palate: Systematic review and meta-analysis. Birth Defects Res A Clin Mol Teratol. 2016 Sep;106(9):773-88. doi: 10.1002/bdra.23540. Epub 2016 Aug 11. PMID: 27511269; PMCID: PMC5095821.

7. <u>RISK OF BIAS TOOL EITHER SELF DESIGNED (NOT VALIDATED OR TESTED FOR RELIABILITY) OR NOT MENTIONED (1- electronic search, 2-other sources, Total=3)</u>

1. Bede-Ojimadu O, Orisakwe OE. Exposure to Wood Smoke and Associated Health Effects in Sub-Saharan Africa: A Systematic Review. Ann Glob Health. 2020 Mar 20;86(1):32. doi: 10.5334/aogh.2725. PMID: 32211302; PMCID: PMC7082829.

2. Kadir A, Mossey PA, Blencowe H, Moorthie S, Lawn JE, Mastroiacovo P, Modell B. Systematic Review and Meta-Analysis of the Birth Prevalence of Orofacial Clefts in Low- and Middle-Income Countries. Cleft Palate Craniofac J. 2017 Sep;54(5):571-581. doi: 10.1597/15-221. Epub 2016 Jul 19. PMID: 27440051.

3. Téblick S, Ruymaekers M, Van de Casteele E, Nadjmi N. Effect of Cleft Palate Closure Technique on Speech and Middle Ear Outcome: A Systematic Review. J Oral Maxillofac Surg. 2019 Feb;77(2):405.e1-405.e15. doi: 10.1016/j.joms.2018.09.027. Epub 2018 Sep 29. PMID: 30367844.

8. <u>OTHER TYPES OF REVIEWS (31- electronic search, 2-other sources, Total=33)</u>

1. de Blacam C, Baylis AL, Kirschner RE, Smith S, Sell D, Sie KCY, Harris HE, Orr DJA. Core Outcome Set for Reporting Outcomes of Interventions for Velopharyngeal Dysfunction: Final Results of the COS-VPD Initiative. Cleft Palate Craniofac J. 2021 Aug 16:10556656211035026. doi: 10.1177/10556656211035026. Epub ahead of print. PMID: 34398725.
2. Allori AC, Kelley T, Meara JG, Albert A, Bonanthaya K, Chapman K, Cunningham M, Daskalogiannakis J, de Gier H, Heggie AA, Hernandez C, Jackson O, Jones Y, Kangesu L, Koudstaal MJ, Kuchhal R, Lohmander A, Long RE Jr, Magee L, Monson L, Rose E, Sitzman TJ, Taylor JA, Thorburn G, van Eeden S, Williams C, Wirthlin JO, Wong KW. A Standard Set of Outcome Measures for the Comprehensive Appraisal of Cleft Care. Cleft Palate Craniofac J. 2017 Sep;54(5):540-554. doi: 10.1597/15-292. Epub 2016 May 25. PMID: 27223626.

3. Chhajlani R, Chhajlani P, Bonanthaya K, Mahajan RK. Alveolar bone grafting and gingivoperiosteoplasty in bilateral cleft lip and palate. Curr Opin Otolaryngol Head Neck Surg. 2021 Aug 1;29(4):327-332. doi: 10.1097/MOO.0000000000000737. PMID: 34091502.

4. Araruna RD, Vendrúscolo DM. Alimentação da criança com fissura de lábio e/ou palato: um estudo bibliográfico. Revista Latino-americana de enfermagem. 2000;8:99-105.

5. Avşar TS, McLeod H, Jackson L. Health outcomes of smoking during pregnancy and the postpartum period: an umbrella review. BMC Pregnancy Childbirth. 2021 Mar 26;21(1):254. doi: 10.1186/s12884-021-03729-1. PMID: 33771100; PMCID: PMC7995767.

6. Bekisz JM, Fryml E, Flores RL. A Review of Randomized Controlled Trials in Cleft and Craniofacial Surgery. J Craniofac Surg. 2018 Mar;29(2):293-301. doi: 10.1097/SCS.0000000000004100. PMID: 29084117.

7. Bichara LM, Araújo RC, Flores-Mir C, Normando D. Impact of primary palatoplasty on the maxillomandibular sagittal relationship in patients with unilateral cleft lip and palate: a systematic review and meta-analysis. Int J Oral Maxillofac Surg. 2015 Jan;44(1):50-6. doi: 10.1016/j.ijom.2014.08.004. Epub 2014 Sep 10. PMID: 25217008.

8. Bogacka-Konik E, Machorowska-Pieniążek A, Iwanecka-Zduńczyk M. Type and efficacy of materials used in alveolar osteoplasty in patients with primary palate cleft.

9. de Blacam C, Baylis AL, Kirschner RE, Smith SM, Sell D, Sie KCY, Harris HE, Orr DJA. Protocol for the development of a core outcome set for reporting outcomes of management of velopharyngeal dysfunction. BMJ Open. 2020 Aug 13;10(8):e036824. doi: 10.1136/bmjopen-2020-036824. PMID: 32792441; PMCID: PMC7430341.

10. Fitzpatrick B, Coad J, Sell D, Rihtman T. Assessing speech at three years of age in the cleft palate population: a scoping review of assessment practices. Int J Lang Commun Disord. 2020 Mar;55(2):165-187. doi: 10.1111/1460-6984.12517. Epub 2020 Feb 20. PMID: 32077212.

11. Fontanals M, Merritt G, Sierra P, Echaniz G. Anesthetic Considerations and Complications of Cleft Palate Repairs. What's New?. Current Anesthesiology Reports. 2021 Sep;11(3):257-64.

12. Garcés Alvear GA, Moreno Soza MIB, Ormeño Quintana ADP, Gutiérrez Melis CM. Complications During Grayson Presurgical Nasoalveolar Molding Method in Nonsyndromic Infants With Complete Unilateral Cleft Lip and Palate. J Craniofac Surg. 2021 Sep 1;32(6):2159-2162. doi: 10.1097/SCS.0000000000007532. PMID: 34516070.

13. Kearney AM, Gart MS, Brandt KE, Gosain AK. Lessons from American Board of Plastic Surgery Maintenance of Certification Tracer Data: A 16-Year Review of Clinical Practice Patterns and Evidence-Based Medicine in Cleft Palate Repair. Plast Reconstr Surg. 2020 Aug;146(2):371-379. doi: 10.1097/PRS.0000000000007018. PMID: 32740590.

14. Lambert EM, You P, Kacmarynski DS, Rosenberg TL. Adenoidectomy and persistent velopharyngeal insufficiency: Considerations, risk factors, and treatment. Int J Pediatr Otorhinolaryngol. 2021 Oct;149:110846. doi: 10.1016/j.ijporl.2021.110846. Epub 2021 Jul 17. PMID: 34329831.

15. Lindeborg MM, Shakya P, Rai SM, Shaye DA. Optimizing speech outcomes for cleft palate. Curr Opin Otolaryngol Head Neck Surg. 2020 Aug;28(4):206-211. doi: 10.1097/MOO.0000000000000635. PMID: 32520757.

16. Lohmander A, Olsson M. Methodology for perceptual assessment of speech in patients with cleft palate: a critical review of the literature. Cleft Palate Craniofac J. 2004 Jan;41(1):64-70. doi: 10.1597/02-136. PMID: 14697067.

17. Mink van der Molen AB, van Breugel JMM, Janssen NG, Admiraal RJC, van Adrichem LNA, Bierenbroodspot F, Bittermann D, van den Boogaard MH, Broos PH, Dijkstra-Putkamer JJM, van Gemert-Schriks MCM, Kortlever ALJ, Mouës-Vink CM, Swanenburg de Veye HFN, van Tol-Verbeek N, Vermeij-Keers C, de Wilde H, Kuijpers-Jagtman AM. Clinical Practice Guidelines on the Treatment of Patients with Cleft Lip, Alveolus, and Palate: An Executive Summary. J Clin Med. 2021 Oct 20;10(21):4813. doi: 10.3390/jcm10214813. PMID: 34768332; PMCID: PMC8584510.

18. Moreau JL, Caccamese JF, Coletti DP, Sauk JJ, Fisher JP. Tissue engineering solutions for cleft palates. J Oral Maxillofac Surg. 2007 Dec;65(12):2503-11. doi: 10.1016/j.joms.2007.06.648. PMID: 18022477.

19. Murthy PS, Deshmukh S, Bhagyalakshmi A, Srilatha K. Pre surgical nasoalveolar molding: changing paradigms in early cleft lip and palate rehabilitation. J Int Oral Health. 2013 Apr;5(2):70-80. PMID: 24155594; PMCID: PMC3768063.

20. Harman NL, Bruce IA, Callery P, Tierney S, Sharif MO, O'Brien K, Williamson PR. MOMENT--Management of Otitis Media with Effusion in Cleft Palate: protocol for a systematic review of the literature and identification of a core outcome set using a Delphi survey. Trials. 2013 Mar 12;14:70. doi: 10.1186/1745-6215-14-70. PMID: 23497540; PMCID: PMC3716725.

21. Pederson H, Okland T, Boyers LN, Karimkhani C, Rosenfeld RM, Nasser M, Yoong SL, Wolfenden L, Kyu HH, Serina PT, Coggeshall M, Dellavalle RP. Identifying otolaryngology systematic review research gaps: comparing Global Burden of Disease 2010 results with Cochrane Database of Systematic Review content. JAMA Otolaryngol Head Neck Surg. 2015 Jan;141(1):67-72. doi: 10.1001/jamaoto.2014.2700. PMID: 25356671.

22. Penny C, McGuire C, Bezuhly M. A Systematic Review of Feeding Interventions for Infants with Cleft Palate. Cleft Palate Craniofac J. 2021 Oct 29:10556656211051216. doi: 10.1177/10556656211051216. Epub ahead of print. PMID: 34714161.

23. Flowers T, Winters R. Postoperative pain management in pediatric cleft lip and palate repair. Curr Opin Otolaryngol Head Neck Surg. 2021 Aug 1;29(4):294-298. doi: 10.1097/MOO.0000000000000719. PMID: 34183559.

24. Rossell-Perry P, Olivencia-Flores C, Delgado-Jimenez MP, Ormeño-Aquino R. Surgical Nasoalveolar Molding: A Rational Treatment for Bilateral Cleft Lip Nose and Systematic Review. Plast Reconstr Surg Glob Open. 2020 Sep 24;8(9):e3082. doi: 10.1097/GOX.0000000000003082. PMID: 33133938; PMCID: PMC7544269.

25. Salimi N, Aleksejūnienė J, Yen EH, Loo AY. Fistula in Cleft Lip and Palate Patients-A Systematic Scoping Review. Ann Plast Surg. 2017 Jan;78(1):91-102. doi: 10.1097/SAP.0000000000000819. PMID: 27015328.

26. Séblain D, Bourlet J, Sigaux N, Khonsari RH, Chauvel Picard J, Gleizal A. Minimally invasive versus standard approach in LeFort 1 osteotomy in patients with history of cleft lip and palate. J Stomatol Oral Maxillofac Surg. 2018 Jun;119(3):187-191. doi: 10.1016/j.jormas.2018.01.005. Epub 2018 Feb 25. PMID: 29408322.

27. Wong Riff KW, Tsangaris E, Goodacre T, Forrest CR, Pusic AL, Cano SJ, Klassen AF. International multiphase mixed methods study protocol to develop a cross-cultural patient-reported outcome instrument for children and young adults with cleft lip and/or palate (CLEFT-Q). BMJ Open. 2017 Jan 11;7(1):e015467. doi: 10.1136/bmjopen-2016-015467. PMID: 28077415; PMCID: PMC5253569.

28. Zbar RIS, Canady JW. An evidence-based approach to secondary cleft lip nasal deformity. Plast Reconstr Surg. 2011 Feb;127(2):905-909. doi: 10.1097/PRS.0b013e31820463be. Erratum in: Plast Reconstr Surg. 2011 Oct;128(4):1001-2. PMID: 21285795.

29. Campolo A, Heider C, Canete I, Verdugo F, Bravo R, Morovic C, Rada G. Use of autologous platelet derivatives for secondary alveoloplasty in patients with cleft lip and palate: a protocol for a systematic review and meta-analysis. medRxiv. 2021 Jan 1.

30. Armstrong R, White S, Streak J, Tivey D. The effect of nasal introduction of anaesthetic in adults undergoing Fibreoptic Endoscopic Evaluation of Swallowing (FEES): A systematic review protocol. JBI Evidence Synthesis. 2013 Jun 1;11(6):259-74.

31. Sainsbury D, Williams C, de Blacam C, Mullen J, Chadha A, Wren Y, Hodgkinson P; Cleft Multidisciplinary Collaborative. Non-Interventional Factors Influencing Velopharyngeal Function For Speech In Initial Cleft Palate Repair: A Systematic Review Protocol. Syst Rev. 2019 Nov 5;8(1):261. doi: 10.1186/s13643-019-1141-2. PMID: 31690349; PMCID: PMC6833250.

32. Liu R, Li F, Deacon SA, Li H, Chen J, Liu Z, Hu H, Wamalwa P, Qin Y, Li C, Zou S. Pre-surgical orthopaedics (PSO) for infants with complete cleft lip and palate. Cochrane Database of Systematic Reviews. 2010(12).

33. Shoaei S, Ghasemian A, Najafi B, Kasaeian A, Farzadfar F, Hessari H. National and sub-national burden of oral diseases in Iran: 1990-2013, study protocol.

9. <u>CONFERENCE ABSTRACT(1- electronic search, 15- other sources, Total=16)</u>

1. Galvao D, Lopes A, Martins C, Sousa S. Breastfeeding children with cleft LIP and/or palate. 2nd World Congress of Health Research. Aten. Prim. 2014:26
2. ACPA's 75th Annual Meeting Abstracts. The Cleft Palate-Craniofacial Journal. 2018;55(1_suppl):1-123. doi:10.1177/1055665618761376

(The Effect of Presurgical Nasoalveolar Molding (NAM) for Cleft Lip/Palate on Long-term Treatment Outcomes: A Systematic Review, Victoria Allen (1), Kathleen Russell)

3. ACPA Annual Meeting 2021 Abstracts. The Cleft Palate-Craniofacial Journal. 2021;58(4_suppl):1-134. doi:10.1177/1055665621999916

(Evaluation of Optimal Outcome Reporting (OOR) in Cleft Palate Repair, Daniel M Balkin)

4. ACPA Annual Meeting 2021 Abstracts. The Cleft Palate-Craniofacial Journal. 2021;58(4_suppl):1-134. doi:10.1177/1055665621999916 (Materials for Cleft Alveolar Bone Grafting: A Systematic Review and Network Meta-Analysis El-Rabbany et al)

5. ACPA's 77th Annual Meeting Abstracts. The Cleft Palate-Craniofacial Journal. 2020;57(4_suppl):1-143. doi:10.1177/1055665620901804

(The Impact of the Buccal Myomucosal Flap on Speech and Surgical Outcomes in Cleft Palate: A Systematic Review, Haenssler and Perry)

6. ACPA's 77th Annual Meeting Abstracts. The Cleft Palate-Craniofacial Journal. 2020;57(4_suppl):1-143. doi:10.1177/1055665620901804

(Evidence-Based Cleft Care in Lower Resource Settings, Kalmar et al)

7. Annual Meeting of the American Academy of Otolaryngology– Head and Neck Surgery Foundation Los Angeles, CA, October 3-6, 2021

(Enhanced Recovery Following Cleft Palate/Lip Repair: Systematic Review and Meta- Analysis Max Shin (Presenter); Connor Wagner; Aman Prasad; Kevin Chorath, MD; Alvaro G. Moreira, MD; Karthik Rajasekaran, MD)

8. ACPA's 77th Annual Meeting Abstracts. The Cleft Palate-Craniofacial Journal. 2020;57(4_suppl):1-143. doi:10.1177/1055665620901804 (In Search of the Optimal Pain Management Strategy for Children Undergoing Cleft Lip and Palate Repair: A Systematic Review and Meta-Analysis Morzycki et al)

9. European Society for Clinical Nutrition and Metabolism (ESPEN),8-11th of September 2012 (A PROPOSAL OF AN ALGORITHM FOR PRE-SURGICAL NUTRITIONAL INTERVENTION IN OROFACIAL CLEFTS BABIES, Nogueira et al)

10. Plastic and Reconstructive Surgery • April 2014 Supplement

(Patient-Reported Outcomes following Cleft Surgery: A Systematic Review, Ranganathan et al)

11. ACPA Annual Meeting 2021 Abstracts. The Cleft Palate-Craniofacial Journal. 2021;58(4_suppl):1-134. Systematic Review of Clinical Practice Guidelines on Oral Health in Children with Cleft Lip and Palate, Saikia et al)

12. H.H. Sung-Hsieh, D.L. Best, C. Ulloa-Marin, Why do we need a systematic review in cleft lip/palate? International Journal of Oral and Maxillofacial Surgery, Volume 48, Supplement 1, 2019, Page 193, ISSN 0901-5027

13. ACPA's 77th Annual Meeting Abstracts. The Cleft Palate-Craniofacial Journal. 2020;57(4_suppl):1-143. doi:10.1177/1055665620901804 (Barriers to Accessing Timely Surgical Cleft Treatment in Low- and Middle-Income Countries, Upadhyaya et al)

14. Oral Abstract, Track 1

(Maxillary Expansion and Timing of Secondary Alveolar Bone Grafting: A Review of the Current Literature, Wang and Chou) https://doi.org/10.1016/j.joms.2014.06.027

15. Wlodarczyk J, Munabi NCO, Higuchi E, et al. Abstract 148: Natural Trajectory Of Midface Growth In Unoperated Cleft Defects: A Systematic Review And Meta-analysis. Plast Reconstr Surg Glob Open. 2020;8(4 Suppl):99-100. Published 2020 May 13. doi:10.1097/01.GOX.0000667648.39868.9d

16. ACPA's 77th Annual Meeting Abstracts. The Cleft Palate-Craniofacial Journal. 2020;57(4_suppl):1-143. doi:10.1177/1055665620901804

(Natural History of Facial Growth in Unoperated Cleft Defects: A Systematic Review and Meta-Analysis, Wlodarczyk et al)

10. <u>UPDATED VERSION OF PAPER INCLUDED (1- electronic search)</u>

1. Kloukos D, Fudalej P, Sequeira-Byron P, Katsaros C. Maxillary distraction osteogenesis versus orthognathic surgery for cleft lip and palate patients. Cochrane database of systematic reviews. 2016(9).

11. MISLEADING INFORMATION (electronic search=1)

1. Stein MJ, Zhang Z, Fell M, Mercer N, Malic C. Determining postoperative outcomes after cleft palate repair: A systematic review and meta-analysis. J Plast Reconstr Aesthet Surg. 2019;72(1):85-91. doi:10.1016/j.bjps.2018.08.019
   (Though the authors mentioned that the quality analysis was done and mentioned in Appendix 2, not only Appendix 2 lacked details of quality analysis, but the quality analysis of included studies were not found to be detailed in any of the appendices)
