# Supplementary material for: Evidence Mapping and Quality Analysis of Systematic Reviews on Various Aspects Related to Cleft Lip and Palate
Source: J Clin Med. 2023 Sep 16;12(18):6002. doi: 10.3390/jcm12186002 (PMC10532364; doi:10.3390/jcm12186002)
Supplement: Supplementary file 1 [file jcm-12-06002-s001.zip › Annexure 4.pdf]

Annexure 4: Detailed characteristics of included studies as Domain, Subdomain I and code, Subdomain II and code (refer to Figure 1 and Table 1 for subdomains and codes), Author and number of reviewers, reference number (as numbered in the reference list of paper), year, journal of publication, language limits, Time limitation, hand/reference searching, grey literature search, number of databases used, number of included studies, Risk of bias tool used for quality assessment and meta-analysis. (Abbreviation Nos.= Numbers, Y= Present, N= Not present). Color codes have been done for different Domains and subdomains (I and II) are as presented in Table 1.

Domain. A. EPIDEMIOLOGIC													
**Sub-domain I - Incidence and Prevalence (A1)**													
N os.	*Subdomain II and code*	*Author and number of reviewers (n)*	*Reference number*	*Year*	*Journal*	*Language limitation*	*Time limitation*	*Hand/ Reference search*	*Grey literature*	*Number of databases used*	*Number of Included studies*	*Risk of Bias Tool Used/ quality assessment*	*Meta-analysis*
1	Anatomical and functional characteristics A6	Tannure et al (7)	21	2012	Cleft Palate Craniofac J	N	Y	Y	N	4	6	Cochrane Collaboration's tool	Y
2	Anatomical and functional characteristics A6	Khalaf et al (4)	22	2014	Journal of Orthodontics	N	Y	Y	Y	2	61	STROBE	Y
3	Embryologic development A3	Allagh et al (8)	23	2015	PLoS One	Y	N	Y	N	2	19	STROBE	Y
4	N/A	Wang et al (11)	24	2017	Birth Defects Research	N	Y	N	N	4	41	Previous studies	Y
5	Etiopathogenesis A2	Chen et al (6)	25	2018	Arch. Gynecol. Obstet.	Y	N	Y	Y	4	34	Newcastle–Ottawa Scale	Y
6	N/A	Haseli et al (5)	26	2018	J Mazandaran Univ Med Sci	Y	N	Y	Y	5	17	ROBINS-I	Y
7	Genetics A4	Pereira et al (6)	27	2019	Scientific Reports	Y	Y	N	Y	2	13	STROBE	N
8	N/A	Salari et al (6)	28	2021	J. Stomatol. Oral Maxillofac. Surg.	Y	Y	Y	Y	4	69	STROBE	Y
**Sub-domain I - Etiopathogenesis (A2)**													
N os.	*Subdomain II and code*	*Author and number of reviewers (n)*	*Reference number*	*Year*	*Journal*	*Language limit*	*Time limitation*	*Hand/ Reference search*	*Grey literature*	*Number of databases used*	*Number of Included studies*	*Risk of Bias Tool Used/ quality assessment*	*Meta-analysis*
9	Embryologic development A3	Chi et al (4)	29	2010	J Am Acad Dermatol., Cochrane library	N	N	Y	Y	4	7	Newcastle–Ottawa Scale	N
10	Embryologic development A3	Dreier et al (3)	30	2013	PEDIATRICS	N	N	Y	N	3	46	Newcastle–Ottawa Scale	Y
11	Embryologic development A3	Molina-Solana et al (5)	31	2013	Int. J. Oral Maxillofac. Surg.	N	N	Y	Y	5	28	Previous study	Y

Annexure 4: Detailed characteristics of included studies as Domain, Subdomain I and code, Subdomain II and code (refer to Figure 1 and Table 1 for subdomains and codes), Author and number of reviewers, reference number (as numbered in the reference list of paper), year, journal of publication, language limits, Time limitation, hand/reference searching, grey literature search, number of databases used, number of included studies, Risk of bias tool used for quality assessment and meta-analysis. (Abbreviation Nos.= Numbers, Y= Present, N= Not present). Color codes have been done for different Domains and subdomains (I and II) are as presented in Table 1.

12	Risk assessment and prediction F2	Popoff et al (6)	32	2013	Dentistry 3000	Y	N	Y	N	3	8	STROBE	N
13	Embryologic development A3	Bell et al (6)	33	2014	Paediatr. Perinat. Epidemiol	N	N	Y	N	3	33	Newcastle–Ottawa Scale	Y
14	Embryologic development A3	Luteijn et al (3)	34	2014	Human Reproduction	Y	N	Y	N	2	46	Newcastle–Ottawa scale	Y
15	Genetics A4	Sabbagh et al (5)	35	2014	Cleft Palate Craniofac J	N	N	Y	Y	2	16	Newcastle–Ottawa Scale	Y
16	Embryologic development A3	De-Regil et al (4)	36	2015	The Cochrane Library	N	N	Y	Y	4	30	Cochrane Collaboration's tool	Y
17	Embryologic development A3	Izedonmwen et al (3)	37	2015	J Oral Maxillofac Res	Y	Y	Y	Y	2	6	Critical Appraisal Skills Programme methodology checklist	Y
18	Embryologic development A3	Sabbagh et al (6)	38	2015	PLoS ONE	N	Y	Y	Y	2	15	Newcastle-Ottawa Scale	Y
19	Embryologic development A3	Tanoshima et al (6)	39	2015	Clin. Pharm. Therap.	N	N	Y	Y	3	59	Newcastle-Ottawa scale	Y
20	Embryologic development A3	Rao et al (4)	40	2016	J Oral Maxillofac Res	Y	Y	Y	Y	2	8	Previous studies	Y
21	Embryologic development A3	Veroniki et al (15)	41	2017	BMC Medicine	N	N	Y	Y	3	93	Cochrane, Newcastle-Ottawa Scale	Y
22	Embryologic development A3	Liang et al (7)	42	2018	Oncotarget	N	N	Y	N	7	16	Newcastle-Ottawa Scale	Y
23	Embryologic development A3	Jayarajan et al (3)	43	2019	Indian J PlastSurg	N	N	N	Y	4	4	Cochrane Collaboration's tool	N
24	Embryologic development A3	Yin et al (4)	44	2019	Br J Oral Maxillofac Surg.	N	N	Y	Y	3	12	Newcastle-Ottawa Scale	Y
25	Embryologic development A3	Picot et al (6)	45	2020	Birth Defects Research.	N	N	unclear	Y	2	12	ROBINS-I	Y
26	Embryologic development A3	Fell et al (6)	46	2021	Cleft Palate Craniofac J	N	N	Y	N	4	44	Newcastle–Ottawa scale	Y

Annexure 4: Detailed characteristics of included studies as Domain, Subdomain I and code, Subdomain II and code (refer to Figure 1 and Table 1 for subdomains and codes), Author and number of reviewers, reference number (as numbered in the reference list of paper), year, journal of publication, language limits, Time limitation, hand/reference searching, grey literature search, number of databases used, number of included studies, Risk of bias tool used for quality assessment and meta-analysis. (Abbreviation Nos.= Numbers, Y= Present, N= Not present). Color codes have been done for different Domains and subdomains (I and II) are as presented in Table 1.

27	Health economics E4	Kruppa et al (5)	47	2021	Cleft Palate Craniofac J	Y	Y	Y	N	4	17	Newcastle-Ottawa Scale	N
28	Embryologic development A3	Shi et al (6)	48	2021	Cleft Palate Craniofac J	Y	N	Y	N	2	13	Newcastle Ottawa Scale	Y
29	Psychosocial aspects, perceptions and quality of life of patients with CLP E1	Tran et al (7)	49	2022	Cleft Palate Craniofac J	Y	N	Y	N	5	12	Newcastle-Ottawa scale	Y
**Sub-domain I - Genetics A4**													
N os.	*Subdomain II and code*	*Author and number of reviewers (n)*	*Refere nce numbe r*	*Year*	*Journal*	*Language limit*	*Time limitation*	*Hand/ Reference search*	*Grey literature*	*Number of databases used*	*Number of Included studies*	*Risk of Bias Tool Used/ quality assessment*	*Meta-analysis*
30	Risk assessment and prediction F2	Machado et al (6)	50	2018	Birth Defects Res	N	N	Y	Y	5	11	MAStARI assessment	Y
31	Etiopathogenesis A2	Imani et al (4)	51	2019	International orthodontics	N	N	Y	N	4	4	Newcastle–Ottawa scale	Y
32	Etiopathogenesis A2	Imani et al (4)	52	2019	Int. J. Environ. Res. Public Health	N	N	Y	N	5	10	Newcastle–Ottawa scale	Y
33	Etiopathogenesis A2	Imani et al (5)	53	2019	International Orthodontics	N	N	Y	N	4	4	Newcastle–Ottawa scale	Y
34	Risk assessment and prediction F2	Mendes et al (7)	54	2019	J Oral Pathol Med.	N	N	Y	Y	4	4	QUADAS-2	N
35	Etiopathogenesis A2	Imani et al (7)	55	2021	Life	N	N	Y	N	4	12	Newcastle–Ottawa scale	Y
**Sub-domain I - Anatomical and functional characteristics A6**													
N os.	*Subdomain II and code*	*Author and number of reviewers (n)*	*Refere nce numbe r*	*Year*	*Journal*	*Language limit*	*Time limitation*	*Hand/ Reference search*	*Grey literature*	*Number of databases used*	*Number of Included studies*	*Risk of Bias Tool Used/ quality assessment*	*Meta-analysis*
36	N/A	Antonarakis et al (3)	56	2013	Clin Oral Invest	N	N	N	Y	5	4	Previous studies	Y

Annexure 4: Detailed characteristics of included studies as Domain, Subdomain I and code, Subdomain II and code (refer to Figure 1 and Table 1 for subdomains and codes), Author and number of reviewers, reference number (as numbered in the reference list of paper), year, journal of publication, language limits, Time limitation, hand/reference searching, grey literature search, number of databases used, number of included studies, Risk of bias tool used for quality assessment and meta-analysis. (Abbreviation Nos.= Numbers, Y= Present, N= Not present). Color codes have been done for different Domains and subdomains (I and II) are as presented in Table 1.

37	Advanced radiography including 3D imaging and 3D printing B6	Berrocal et al (7)	57	2014	British Institute of Radiology	Y	N	Y	Y	6	15	Previous studies	Y
38	Advanced radiography including 3D imaging and 3D printing B6	Kuijpers et al (5)	58	2014	PLoS ONE	N	N	Y	N	5	144	Previous study	N
39	N/A	Boyce et al (3)	59	2017	Child Care Health Dev.	Y	N	N	N	5	18	Scottish Intercollegiate Guidelines Network	N
40	N/A	Van Dyck et al (4)	60	2019	Forensic Science International	Y	N	Y	Y	3	36	MINORS	N
41	N/A	Gallego et al (4)	61	2021	Nutr Hosp	Y	Y	N	N	2	6	National Heart, Lung and Blood Institute-Quality Assessment Tool	N
42	Etiopathogenesis A2	Marzouk et al (14)	62	2021	JDR Clin. Transl. Res.	N	N	Y	Y	6	51	modified Newcastle-Ottawa Scale	Y
43	Conventional radiography and 2D imaging B5	Murugan and Kailasam (2)	63	2021	Cleft Palate Craniofac J	Y	N	N	N	6	15	Modified Newcastle Ottawa Scale	Y
44	N/A	Southby et al (5)	64	2021	Int J Lang CommunDisord.	Y	N	Y	Y	8	6	CASP	N
45	N/A	Wu et al (7)	65	2021	Cleft Palate Craniofac J	Y	N	Y	N	4	13	Newcastle-Ottawa Scale	N
46	Advanced radiography including 3D imaging and 3D printing B6	Srivastav et al (8)	66	2022	Cleft Palate Craniofac J	N	N	Y	Y	6	6	JBI	Y
**Domain. B. DIAGNOSTIC**													

Annexure 4: Detailed characteristics of included studies as Domain, Subdomain I and code, Subdomain II and code (refer to Figure 1 and Table 1 for subdomains and codes), Author and number of reviewers, reference number (as numbered in the reference list of paper), year, journal of publication, language limits, Time limitation, hand/reference searching, grey literature search, number of databases used, number of included studies, Risk of bias tool used for quality assessment and meta-analysis. (Abbreviation Nos.= Numbers, Y= Present, N= Not present). Color codes have been done for different Domains and subdomains (I and II) are as presented in Table 1.

**Sub-domain I - Indices of CLP B2**													
N os.	*Subdomain II and code*	*Author and number of reviewers (n)*	*Reference number*	*Year*	*Journal*	*Language limit*	*Time limitation*	*Hand/ Reference search*	*Grey literature*	*Number of databases used*	*Number of Included studies*	*Risk of Bias Tool Used/ quality assessment*	*Meta-analysis*
47	Outcome assessment C15	Altalibi et al (5)	67	2013	Eur J Orthod.	N	N	Y	Y	7	13	QUADAS methodologic assessment tool	N
48	Treatment planning and prediction C1	Buj-Acosta et al (5)	68	2017	PLoS ONE	N	N	Y	Y	4	3	QUADAS tool	N
**Sub-domain I - Prenatal diagnosis B3**													
N os.	*Subdomain II and code*	*Author and number of reviewers (n)*	*Reference number*	*Year*	*Journal*	*Language limit*	*Time limitation*	*Hand/ Reference search*	*Grey literature*	*Number of databases used*	*Number of Included studies*	*Risk of Bias Tool Used/ quality assessment*	*Meta-analysis*
49	Conventional radiography and 2D imaging B5	Lai et al (5)	69	2021	J Ultrasound Med	N	Y	Y	N	3	7	QUADAS-2	Y
**Sub-domain I - Advanced radiography including 3D imaging and 3D printing B6**													
N os.	*Subdomain II and code*	*Author and number of reviewers (n)*	*Reference number*	*Year*	*Journal*	*Language limit*	*Time limitation*	*Hand/ Reference search*	*Grey literature*	*Number of databases used*	*Number of Included studies*	*Risk of Bias Tool Used/ quality assessment*	*Meta-analysis*
50	Clinical practice guidelines and Core outcome sets G1	van Vlijmen et al (8)	70	2012	JADA	N	N	Y	N	5	50	Previous studies	N
51	Anatomical and functional characteristics A6	Thierens et al (4)	71	2018	J Oral MaxillofacSurg	Y	N	Y	N	4	15	MINORS	N
52	Anatomical and functional characteristics A6	Thierens et al (4)	72	2018	Int. J. Oral Maxillofac. Surg.	Y	N	Y	N	4	33	MINORS	N
**Domain. C. THERAPEUTIC**													
**Sub-domain I -. Feeding and nutrition in patients C2**													
N os.	*Subdomain II and code*	*Author and number of reviewers (n)*	*Reference*	*Year*	*Journal*	*Language Limit*	*Time limitation*	*Hand/ Reference search*	*Grey literature*	*Number of databases used*	*Number of*	*Risk of Bias Tool Used/ quality assessment*	*Meta-analysis*

Annexure 4: Detailed characteristics of included studies as Domain, Subdomain I and code, Subdomain II and code (refer to Figure 1 and Table 1 for subdomains and codes), Author and number of reviewers, reference number (as numbered in the reference list of paper), year, journal of publication, language limits, Time limitation, hand/reference searching, grey literature search, number of databases used, number of included studies, Risk of bias tool used for quality assessment and meta-analysis. (Abbreviation Nos.= Numbers, Y= Present, N= Not present). Color codes have been done for different Domains and subdomains (I and II) are as presented in Table 1.

			numbe r								*Included studies*		
53	Clinical practice guidelines and Core outcome sets G1	Mei et al (3)	73	2009	Evidence-Based Communication Assessment and Intervention	Y	N	N	N	5	14	Cochrane Collaboration's tool	N
**Sub-domain I -. Presurgical Infant Orthopaedics and Naso-Alveolar Moulding C3**													
N os.	*Subdomain II and code*	*Author and number of reviewers (n)*	*Refere nce numbe r*	*Year*	*Journal*	*Language limit*	*Time limitation*	*Hand/ Reference search*	*Grey literature*	*Number of databases used*	*Number of Included studies*	*Risk of Bias Tool Used/ quality assessment*	*Meta-analysis*
54	Cleft Lip repair C8	Heijden et al (7)	74	2012	Plast. Reconstr. Surg.	Y	N	Y	N	3	12	GRADE	N
55	Outcome assessment C15	Namdar et al (5)	75	2020	Journal of Pediatrics Review	Y	N	Y	N	3	22	Cochrane Collaboration's tool	N
56	Outcome assessment C15	Padovano et al (5)	76	2021	Cleft Palate Craniofac J	Y	N	N	Y	5	12	modified Newcastle-Ottawa scale, Agency for Healthcare Research and Quality (AHRQ) standards, Cochrane collaboration risk of bias tool	Y
**Sub-domain I - Orthodontic and Surgical Perspectives of Secondary Alveolar Bone Grafting C5**													
N os.	*Subdomain II and code*	*Author and number of reviewers (n)*	*Refere nce numbe r*	*Year*	*Journal*	*Language limit*	*Time limitation*	*Hand/ Reference search*	*Grey literature*	*Number of databases used*	*Number of Included studies*	*Risk of Bias Tool Used/ quality assessment*	*Meta-analysis*
57	Advanced radiography including 3D imaging and 3D printing B6	Mulder et al (5)	77	2019	DentomaxillofacRadiol	Y	N	Y	Y	3	38	MINORS	N

Annexure 4: Detailed characteristics of included studies as Domain, Subdomain I and code, Subdomain II and code (refer to Figure 1 and Table 1 for subdomains and codes), Author and number of reviewers, reference number (as numbered in the reference list of paper), year, journal of publication, language limits, Time limitation, hand/reference searching, grey literature search, number of databases used, number of included studies, Risk of bias tool used for quality assessment and meta-analysis. (Abbreviation Nos.= Numbers, Y= Present, N= Not present). Color codes have been done for different Domains and subdomains (I and II) are as presented in Table 1.

58	Advanced radiography including 3D imaging and 3D printing B6	Scalzone et al (6)	78	2019	Progress in Orthodontics	N	Y	Y	N	4	4	GRADE	Y
59	Alveolar bone grafting and graft materials C10	Pinheiro et al (5)	79	2020	OrthodCraniofac Res.	N	N	Y	Y	5	19	MINORS	N
60	Outcome assessment C15	Yu et al (3)	80	2020	Oral And Maxillofacial Radiology	N	Y	Y	Y	3	11	MINORS	N
61	Alveolar bone grafting and graft materials C10	Jahanbin et al (5)	81	2021	J Craniofac Surg.	N	N	Y	Y	5	8	Newcastle-Ottawa evaluation (NRCT) and Cochrane Collaboration’s tool for RCT	Y
62	Outcome assessment C15	Lacerda-Santos et al (8)	82	2021	Eur. J. Dent.	N	N	Y	Y	5	4	ROBINS-I	N
**Sub-domain I - Conventional orthodontics C6**													
N os.	*Subdomain II and code*	*Author and number of reviewers (n)*	*Reference number*	*Year*	*Journal*	*Language limit*	*Time limitation*	*Hand/ Reference search*	*Grey literature*	*Number of databases used*	*Number of Included studies*	*Risk of Bias Tool Used/ quality assessment*	*Meta-analysis*
63	Outcome assessment C15	Palikaraki et al (7)	83	2020	Eur J Orthod.	Y	N	Y	Y	5	9	ROBINS-I	Y
64	Outcome assessment C15	Shafaee et al (4)	84	2020	Iran J Ortho.	N	N	Y	Y	3	9	Newcastle-Ottawa Scale	Y
**Sub-domain I - Cleft Lip repair C8**													
N os.	*Subdomain II and code*	*Author and number of reviewers (n)*	*Reference number*	*Year*	*Journal*	*Language limit*	*Time limitation*	*Hand/ Reference search*	*Grey literature*	*Number of databases used*	*Number of Included studies*	*Risk of Bias Tool Used/ quality assessment*	*Meta-analysis*
65	Effects/Complications of PSIO and PNAM D2	Thierens et al (4)	85	2016	Oral Dis.	Y	N	Y	N	4	15	MINORS	N
66	Outcome assessment C15	Jayarajan et al (3)	86	2018	Cleft Palate Craniofac J	N	N	N	N	4	16	Cochrane Collaboration’s tool	N
**Sub-domain I - Cleft alveolus, sub-mucous cleft and all types of cleft palate closure/repair C9**													

Annexure 4: Detailed characteristics of included studies as Domain, Subdomain I and code, Subdomain II and code (refer to Figure 1 and Table 1 for subdomains and codes), Author and number of reviewers, reference number (as numbered in the reference list of paper), year, journal of publication, language limits, Time limitation, hand/reference searching, grey literature search, number of databases used, number of included studies, Risk of bias tool used for quality assessment and meta-analysis. (Abbreviation Nos.= Numbers, Y= Present, N= Not present). Color codes have been done for different Domains and subdomains (I and II) are as presented in Table 1.

N os.	*Subdomain II and code*	*Author and number of reviewers (n)*	*Reference number*	*Year*	*Journal*	*Language limit*	*Time limitation*	*Hand/ Reference search*	*Grey literature*	*Number of databases used*	*Number of Included studies*	*Risk of Bias Tool Used/ quality assessment*	*Meta-analysis*
67	Effects/Complications of Surgical treatment and/or alveolar bone grafting D4	Salgado et al (6)	87	2019	J CraniomaxillofacSurg	N	N	Y	Y	5	5	Previous study	N
**Sub-domain I - Alveolar bone grafting and graft materials C10**													
N os.	*Subdomain II and code*	*Author and number of reviewers (n)*	*Reference number*	*Year*	*Journal*	*Language limit*	*Time limitation*	*Hand/ Reference search*	*Grey literature*	*Number of databases used*	*Number of Included studies*	*Risk of Bias Tool Used/ quality assessment*	*Meta-analysis*
68	Orthodontic and Surgical Perspectives of Secondary Alveolar Bone Grafting C5	Guo et al (9)	88	2011	The Cochrane Library	N	N	Y	Y	4	2	Cochrane Collaboration's tool and ROBINS-I guidelines	Y
69	Orthodontic and Surgical Perspectives of Secondary Alveolar Bone Grafting C5	Canellas et al (5)	89	2018	Int. J. Oral Maxillofac. Surg.	N	N	Y	Y	5	30	Cochrane Collaboration's tool	Y
70	Orthodontic and Surgical Perspectives of Secondary Alveolar Bone Grafting C5	da Rosa et al (5)	90	2018	Calcif Tissue Int.	Y	N	Y	N	6	10	Cochrane Collaboration's tool	Y
71	Advanced radiography including 3D imaging and 3D printing B6	Kamal et al (8)	91	2018	Br. J. Oral Maxillofac. Surg.	N	N	Y	Y	4	32	MOOSE, STROBE	Y
72	Effects/Complications of Surgical treatment and/or alveolar bone grafting D4	Simpson et al (5)	92	2018	Cleft Palate Craniofac J	Y	N	Y	N	2	12	MINORS	Y

Annexure 4: Detailed characteristics of included studies as Domain, Subdomain I and code, Subdomain II and code (refer to Figure 1 and Table 1 for subdomains and codes), Author and number of reviewers, reference number (as numbered in the reference list of paper), year, journal of publication, language limits, Time limitation, hand/reference searching, grey literature search, number of databases used, number of included studies, Risk of bias tool used for quality assessment and meta-analysis. (Abbreviation Nos.= Numbers, Y= Present, N= Not present). Color codes have been done for different Domains and subdomains (I and II) are as presented in Table 1.

73	Comprehensive Interdisciplinary care C13	Sales et al (4)	93	2019	Int. J. Oral Maxillofac. Surg.	N	N	Y	Y	3	11	Newcastle-Ottawa Scale	N
74	Cleft alveolus, submucous cleft and all types of cleft palate closure/repair C9	Uribe et al (5)	94	2019	Cleft Palate Craniofac J	Y	N	N	N	6	5	Cochrane Collaboration’s tool	N
75	Orthodontic and Surgical Perspectives of Secondary Alveolar Bone Grafting C5	Francisco et al (3)	95	2020	Materials	Y	N	Y	N	4	9	Cochrane Risk of Bias tool and ROBINS-I guidelines	N
76	Presurgical Infant Orthopaedics and Naso-Alveolar Moulding C3	Ma et al (4)	96	2020	J Stomatol Oral MaxillofacSurg	Y	N	unclear	unclear	5	11	MINORS	N
77	Outcome assessment C15	Osorio et al (5)	97	2020	Heliyon	N	N	Y	N	3	15	Cochrane Collaboration’s tool	N
78	Outcome assessment C15	Xiao et al (4)	98	2020	J PlastReconstrAesthet	N	N	Y	N	4	9	Newcastle–Ottawa Scale	Y
79	Orthodontic and Surgical Perspectives of Secondary Alveolar Bone Grafting C5	Alkaabi et al (7)	99	2021	J PlastReconstrAesthetSurg .	Y	N	N	Y	4	15	Jadad scale and the Delphi list	Y
80	Outcome assessment C15	Carbullido et al (9)	100	2021	Cleft Palate Craniofac J	Y	N	N	N	3	32	Newcastle–Ottawa Scale	N
81	Orthodontic and Surgical Perspectives of Secondary Alveolar Bone Grafting C5	Chetpakdeechit et al (4)	101	2021	Cleft Palate Craniofac J	N	N	N	N	4	23	ROBINS-I	N

Annexure 4: Detailed characteristics of included studies as Domain, Subdomain I and code, Subdomain II and code (refer to Figure 1 and Table 1 for subdomains and codes), Author and number of reviewers, reference number (as numbered in the reference list of paper), year, journal of publication, language limits, Time limitation, hand/reference searching, grey literature search, number of databases used, number of included studies, Risk of bias tool used for quality assessment and meta-analysis. (Abbreviation Nos.= Numbers, Y= Present, N= Not present). Color codes have been done for different Domains and subdomains (I and II) are as presented in Table 1.

82	Orthodontic and Surgical Perspectives of Secondary Alveolar Bone Grafting C5	Deshpande et al (3)	102	2021	Int J Dentistry Oral Sci.	Y	N	Y	Y	2	3	CONSORT guidelines	N
**Sub-domain I - Orthognathic and osteogenic distraction surgical orthodontics C11**													
Nos.	*Subdomain II and code*	*Author and number of reviewers (n)*	*Reference number*	*Year*	*Journal*	*Language limit*	*Time limitation*	*Hand/ Reference search*	*Grey literature*	*Number of databases used*	*Number of Included studies*	*Risk of Bias Tool Used/ quality assessment*	*Meta-analysis*
83	Outcome assessment C15	Saltaji et al (5)	103	2012	J Oral MaxillofacSurg	N	N	Y	Y	5	10	Previous study	N
84	Outcome assessment C15	Saltaji et al (5)	104	2012	Angle Orthod.	N	N	Y	Y	5	13	Previous study	N
85	Outcome assessment C15	Viwattanatipa et al (3)	105	2015	Orthodontic waves	Y	Y	N	Y	5	12	Previous study	Y
86	Outcome assessment C15	Jiang et al (8)	106	2020	Int. J. Oral Maxillofac. Surg.	Y	N	N	N	4	141	MINORS	Y
**Sub-domain I - Other surgical aspects of cleft lip and palate including revisions, scar management, post-operative pain and anaesthesia C12**													
Nos.	*Subdomain II and code*	*Author and number of reviewers (n)*	*Reference number*	*Year*	*Journal*	*Language limit*	*Time limitation*	*Hand/ Reference search*	*Grey literature*	*Number of databases used*	*Number of Included studies*	*Risk of Bias Tool Used/ quality assessment*	*Meta-analysis*
87	Effects/Complications of Surgical treatment and/or alveolar bone grafting D4	Suresh et al (4)	107	2014	British Journal of Anaesthesia	N	N	Y	N	3	73	modified Jadad five-point quality scale	N
88	Cleft Lip repair C8	Feriani et al (4)	108	2016	The Cochrane Library	N	N	Y	Y	3	8	Cochrane Collaboration's tool	Y
89	Effects/Complications of Surgical treatment and/or alveolar bone grafting D4	Kendall et al (5)	109	2018	Local Reg. Anesth.	N	Y	Y	Y	2	40	Modified Jadad score	N
90	Alveolar bone grafting and graft materials C10	Tache and Mommaerts (2)	110	2019	Int. J. Oral Maxillofac. Surg.	Y	N	Y	N	4	15	MINORS, Cochrane Collaboration's tool	N
91	Cleft alveolus, submucous cleft and all types of cleft palate closure/repair C9	Liu et al (8)	111	2021	BMJ Open	Y	N	Y	Y	6	13	Cochrane Collaboration's tool	Y
**Sub-domain I - Management of speech and hearing problems C14**													

Annexure 4: Detailed characteristics of included studies as Domain, Subdomain I and code, Subdomain II and code (refer to Figure 1 and Table 1 for subdomains and codes), Author and number of reviewers, reference number (as numbered in the reference list of paper), year, journal of publication, language limits, Time limitation, hand/reference searching, grey literature search, number of databases used, number of included studies, Risk of bias tool used for quality assessment and meta-analysis. (Abbreviation Nos.= Numbers, Y= Present, N= Not present). Color codes have been done for different Domains and subdomains (I and II) are as presented in Table 1.

N os.	*Subdomain II and code*	*Author and number of reviewers (n)*	*Refere nce numbe r*	*Year*	*Journal*	*Language limit*	*Time limitation*	*Hand/ Reference search*	*Grey literature*	*Number of databases used*	*Number of Included studies*	*Risk of Bias Tool Used/ quality assessment*	*Meta-analysis*
92	Cleft alveolus, submucous cleft and all types of cleft palate closure/repair C9	Nasser et al (3)	112	2008	The Cochrane Library	N	N	Y	Y	6	1	Cochrane Collaboration's tool	N
93	N/A	Lee et al (3)	113	2009	The Cochrane Library	N	N	Y	Y	10	1	Cochrane Collaboration's tool	N
94	N/A	Meinusch and Romonath (2)	114	2011	Evidence-Based Communication Assessment and Intervention	Y	N	Y	Y	10	5	Cochrane Collaboration's tool	N
95	N/A	Bessell et al (9)	115	2013	Cleft Palate Craniofac J	N	N	Y	Y	7	17	Cochrane Collaboration's tool	Y
96	Cleft alveolus, submucous cleft and all types of cleft palate closure/repair C9	Gilleard et al (6)	116	2014	Cleft Palate Craniofac J	Y	N	Y	Y	9	26	Previous studies	N
97	Effects/Complications of Surgical treatment and/or alveolar bone grafting D4	Kuo et al (7)	117	2014	PEDIATRICS	N	N	Y	Y	3	9	Previous studies	N
98	N/A	Lane et al (3)	118	2020	Int J Lang CommunDisord.	Y	N	N	Y	6	7	Physiotherapy Evidence Database quality assessment tool (PEDro-P), single-case experimental design (SCED)	N
99	Outcome assessment C15	Sand et al (3)	119	2022	J. Speech Lang. Hear. Re.	Y	N	Y	Y	6	21	ROBINS-I	Y
**Sub-domain I - Outcome assessment C15**													
N os.	*Subdomain II and code*	*Author and number of reviewers (n)*	*Refere nce numbe r*	*Year*	*Journal*	*Language limit*	*Time limitation*	*Hand/ Reference search*	*Grey literature*	*Number of databases used*	*Number of Included studies*	*Risk of Bias Tool Used/ quality assessment*	*Meta-analysis*

Annexure 4: Detailed characteristics of included studies as Domain, Subdomain I and code, Subdomain II and code (refer to Figure 1 and Table 1 for subdomains and codes), Author and number of reviewers, reference number (as numbered in the reference list of paper), year, journal of publication, language limits, Time limitation, hand/reference searching, grey literature search, number of databases used, number of included studies, Risk of bias tool used for quality assessment and meta-analysis. (Abbreviation Nos.= Numbers, Y= Present, N= Not present). Color codes have been done for different Domains and subdomains (I and II) are as presented in Table 1.

100	Feeding and nutrition in patients C2	Bessell et al (6)	120	2011	The Cochrane Library	N	N	Y	Y	5	8	Cochrane Collaboration’s risk of bias tool	N
101	Orthognathic and osteogenic distraction surgical orthodontics C11	Sharshar et al (2)	121	2012	Cleft Palate Craniofac J	N	N	Y	Y	6	4	Previous study	N
102	Orthognathic and osteogenic distraction surgical orthodontics C11	Austin et al (3)	122	2015	OrthodCraniofac Res	Y	N	Y	Y	6	5	Cochrane Collaboration’s risk of bias tool	N
103	Psychosocial aspects, perceptions and quality of life of patients with CLP E2	Norman et al (10)	123	2015	Cleft Palate Craniofac J	N	N	Y	Y	9	2	Cochrane risk of bias assessment tool, RAMbo Assessment of Observational Studies	N
104	Presurgical Infant Orthopaedics and Naso-Alveolar Moulding C3	Hosseini et al (3)	124	2017	PLoS One	N	N	Y	Y	9	20	Cochrane Collaboration's Risk of Bias assessment tool for RCTs	Y
105	Cleft alveolus, submucous cleft and all types of cleft palate closure/repair C9	El-Ashmawi et al (5)	125	2018	Cleft Palate Craniofac J	Y	N	Y	Y	5	13	ROBINS-I	Y
106	Orthognathic and osteogenic distraction surgical orthodontics C11	Kloukos et al (4)	126	2018	The Cochrane Library	N	N	Y	Y	4	1	Cochrane’s tool for assessing risk of bias	N
107	Advanced radiography including 3D imaging and 3D printing B6	Awarun et al (5)	127	2019	DentomaxillofacRadiol.	Y	N	Y	N	4	12	QUADAS-2 protocol	N
108	Indices of CLP B2	Kuang et al (5)	128	2021	Cleft Palate Craniofac J	N	N	Y	Y	4	8	ROBINS-I	N
109	Orthognathic and osteogenic distraction surgical orthodontics C11	Nimbalkar et al (5)	129	2021	Eur J Orthod.	Y	N	unclear	N	3	9	Newcastle-Ottawa scale (NS)	Y
110	Presurgical Infant Orthopaedics and Naso-Alveolar Moulding C3	Sazo et al (4)	130	2021	Odontoestomatología	Y	Y	Y	N	2	25	The Cochrane Collaboration (2011) guideline	N
**Domain. D. PROGNOSTIC**													

Annexure 4: Detailed characteristics of included studies as Domain, Subdomain I and code, Subdomain II and code (refer to Figure 1 and Table 1 for subdomains and codes), Author and number of reviewers, reference number (as numbered in the reference list of paper), year, journal of publication, language limits, Time limitation, hand/reference searching, grey literature search, number of databases used, number of included studies, Risk of bias tool used for quality assessment and meta-analysis. (Abbreviation Nos.= Numbers, Y= Present, N= Not present). Color codes have been done for different Domains and subdomains (I and II) are as presented in Table 1.

**Sub-domain I - Effects/Complications of Surgical treatment and/or alveolar bone grafting D4**													
N os.	*Subdomain II and code*	*Author and number of reviewers (n)*	*Reference number*	*Year*	*Journal*	*Language limit*	*Time limitation*	*Hand/ Reference search*	*Grey literature*	*Number of databases used*	*Number of Included studies*	*Risk of Bias Tool Used/ quality assessment*	*Meta-analysis*
111	Cleft alveolus, submucous cleft and all types of cleft palate closure/ repair C9	Hardwicke et al (3)	131	2014	Plast. Reconstr. Surg.	Y	Y	N	N	4	44	RCT- Detsky scale, Nnrct- Methodological Index for Nn-randomized Studies	N
112	Cleft alveolus, submucous cleft and all types of cleft palate closure/repair C9	Felton et al (5)	132	2017	AAO-HNSF	N	N	Y	Y	3	6	Oxford Centre for Evidence-Based Medicine levels of evidence, Cochrane Handbook	N
113	Cleft alveolus, submucous cleft and all types of cleft palate closure/repair C9	Kappen et al (4)	133	2018	J PlastReconstrAesthetSurg	Y	N	Y	Y	4	17	Newcastle–Ottawa scale, Agency for Health Care Research and Quality (AHRQ)	Y
114	Orthognathic and osteogenic distraction surgical orthodontics C11	Liu et al (2)	134	2018	J. Craniofac. Surg.	Y	N	Y	Y	6	9	Previous study	N
115	Orthognathic and osteogenic distraction surgical orthodontics C11	Dos Santos Alves et al (6)	135	2019	Int. J. Oral Maxillofac. Surg.	Y	N	Y	N	5	32	Previous study	N
116	Cleft alveolus, submucous cleft and all types of cleft palate closure/repair C9	Minatel et al (5)	136	2019	J. Cranio-Maxillo-Fac. Surg.	N	N	N	N	3	13	Newcastle-Ottawa scale	N
117	Cleft alveolus, submucous cleft and all types of cleft palate closure/repair C9	Tache and Mommaerts (2)	137	2019	Cleft Palate Craniofac J	N	N	Y	N	4	36	MINORS	N
118	Conventional radiography and 2D imaging B4	Corthouts et al (4)	138	2020	PlastAesthet Res	N	Y	Y	Y	2	11	Previous study	N

Annexure 4: Detailed characteristics of included studies as Domain, Subdomain I and code, Subdomain II and code (refer to Figure 1 and Table 1 for subdomains and codes), Author and number of reviewers, reference number (as numbered in the reference list of paper), year, journal of publication, language limits, Time limitation, hand/reference searching, grey literature search, number of databases used, number of included studies, Risk of bias tool used for quality assessment and meta-analysis. (Abbreviation Nos.= Numbers, Y= Present, N= Not present). Color codes have been done for different Domains and subdomains (I and II) are as presented in Table 1.

119	Other surgical aspects of cleft lip and palate including revisions, scar management, post-operative pain and anaesthesia C12	Miranda et al (5)	139	2020	J Craniofac Surg.	N	N	N	N	4	18	MINORS	N
120	Cleft alveolus, submucous cleft and all types of cleft palate closure/repair C9	Vandenberg et al (4)	140	2020	Cleft Palate Craniofac J	Y	N	Y	N	4	9	Newcastle-Ottawa scale	Y
121	Orthognathic and osteogenic distraction surgical orthodontics C11	Velasquese al (5)	141	2020	Oral and Maxillofacial Surgery	Y	N	Y	Y	3	10	PRISMA	N
122	Cleft Lip repair C8	Egbunah et al (6)	142	2021	Cleft Palate Craniofac J	Y	N	Y	Y	2	6	Previous study	Y
123	Orthognathic and osteogenic distraction surgical orthodontics C11	Valls-Ontanon et al (4)	143	2021	Journal of Cranio-Maxillo-Facial Surgery	Y	N	Y	Y	2	29	Previous study, GRADE	Y
**Sub-domain I - Oral health aspects including dental caries and other dental problems D6**													
Nos.	*Subdomain II and code*	*Author and number of reviewers (n)*	*Reference number*	*Year*	*Journal*	*Language limit*	*Time limitation*	*Hand/ Reference search*	*Grey literature*	*Number of databases used*	*Number of Included studies*	*Risk of Bias Tool Used/ quality assessment*	*Meta-analysis*
124	N/A	Worth et al (6)	144	2017	British Dental Journal	N	N	Y	Y	10	24	Previous study (Agbaje et al)	Y
125	Conventional orthodontics C6	Vale et al (5)	145	2021	Int J Dent Hygiene.	Y	N	N	N	5	2	ROBINS-I	Y
**Domain. E. PSYCHOSOCIAL ASPECTS, PERCEPTIONS AND QUALITY OF LIFE**													
**Sub-domain I - Psychosocial aspects, perceptions and quality of life of family members of the patients with CLP, lay people and healthcare professionals E1**													
Nos.	*Subdomain II and code*	*Author and number of reviewers (n)*	*Reference number*	*Year*	*Journal*	*Language limit*	*Time limitation*	*Hand/ Reference search*	*Grey literature*	*Number of databases used*	*Number of Included studies*	*Risk of Bias Tool Used/ quality assessment*	*Meta-analysis*
126	N/A	Zhu et al (3)	146	2016	Cleft Palate Craniofac J	N	N	Y	N	5	11	Previous study	N

Annexure 4: Detailed characteristics of included studies as Domain, Subdomain I and code, Subdomain II and code (refer to Figure 1 and Table 1 for subdomains and codes), Author and number of reviewers, reference number (as numbered in the reference list of paper), year, journal of publication, language limits, Time limitation, hand/reference searching, grey literature search, number of databases used, number of included studies, Risk of bias tool used for quality assessment and meta-analysis. (Abbreviation Nos.= Numbers, Y= Present, N= Not present). Color codes have been done for different Domains and subdomains (I and II) are as presented in Table 1.

127	Presurgical Infant Orthopaedics and Naso-Alveolar Moulding C3	El-Ghafour et al (5)	147	2020	Maced J Med Sci	Y	N	Y	Y	7	1	Cochrane Collaboration's tool	N
128	Feeding and nutrition in patients C2	Srivastav et al (6)	148	2021	Spec Care Dentist.	N	N	Y	Y	5	8	JBI	N
**Sub-domain I - Psychosocial aspects, perceptions and quality of life of patients with CLP E2**													
N os.	*Subdomain II and code*	*Author and number of reviewers (n)*	*Reference number*	*Year*	*Journal*	*Language limit*	*Time limitation*	*Hand/ Reference search*	*Grey literature*	*Number of databases used*	*Number of Included studies*	*Risk of Bias Tool Used/ quality assessment*	*Meta-analysis*
129	N/A	Antonarakis et al (3)	149	2013	Community Dent. Health	N	N	Y	Y	6	3	Cochrane Collaboration's tool	N
130	N/A	Herkrath et al (4)	150	2015	Cleft Palate Craniofac J	Y	N	Y	N	8	23	STROBE	Y
131	N/A	Chung et al (5)	151	2019	PlastReconstrSurg Glob Open	Y	Y	Y	Y	3	15	Joanna Briggs Institute (JBI) checklist	N
132	Other surgical aspects of cleft lip and palate including revisions, scar management, post-operative pain and anaesthesia C12	Acum et al (6)	152	2020	Cleft Palate Craniofac J	Y	N	Y	N	5	22	tailored version of the SIGN-50 rating tool	N
133	Anatomical and functional characteristics A6	Glinianaia et al (6)	153	2021	Birth Defects Research	Y	N	Y	Y	5	39	Newcastle–Ottawa Scale (modified)	Y
134	Conventional orthodontics C6	Nascimento et al (5)	154	2021	Eur J Orthod.	N	N	Y	Y	6	13	Fowkes and Fulton qualifier	Y
**Sub-domain I - Burden of care E3**													
N os.	*Subdomain II and code*	*Author and number of reviewers (n)*	*Reference number*	*Year*	*Journal*	*Language limit*	*Time limitation*	*Hand/ Reference search*	*Grey literature*	*Number of databases used*	*Number of Included studies*	*Risk of Bias Tool Used/ quality assessment*	*Meta-analysis*
135	Presurgical Infant Orthopaedics and Naso-Alveolar Moulding C3	Alfonso et al (8)	c	2020	Cleft Palate Craniofac J	Y	N	N	N	5	29	Cochrane risk-of-bias, ROBINS-I Scale, CLARITY Group Risk of Bias Instrument	N

Annexure 4: Detailed characteristics of included studies as Domain, Subdomain I and code, Subdomain II and code (refer to Figure 1 and Table 1 for subdomains and codes), Author and number of reviewers, reference number (as numbered in the reference list of paper), year, journal of publication, language limits, Time limitation, hand/reference searching, grey literature search, number of databases used, number of included studies, Risk of bias tool used for quality assessment and meta-analysis. (Abbreviation Nos.= Numbers, Y= Present, N= Not present). Color codes have been done for different Domains and subdomains (I and II) are as presented in Table 1.

**Sub-domain I - Health economics E4**													
N os.	*Subdomain II and code*	*Author and number of reviewers (n)*	*Reference number*	*Year*	*Journal*	*Language limit*	*Time limitation*	*Hand/ Reference search*	*Grey literature*	*Number of databases used*	*Number of Included studies*	*Risk of Bias Tool Used/ quality assessment*	*Meta-analysis*
136	Burden of care E3	Saxton et al (7)	156	2016	PLoS One	Y	Y	Y	Y	3	86	Previous study	N
**Domain F. PREVENTIVE**													
**Sub-domain I - Risk assessment and prediction F2**													
N os.	*Subdomain II and code*	*Author and number of reviewers (n)*	*Reference number*	*Year*	*Journal*	*Language limit*	*Time limitation*	*Hand/ Reference search*	*Grey literature*	*Number of databases used*	*Number of Included studies*	*Risk of Bias Tool Used/ quality assessment*	*Meta-analysis*
137	Genetics A4	Zhao et al (8)	157	2016	F1000Research	N	N	Y	Y	5	10	CONSORT, STROBE	Y
138	Genetics A4	Wang et al (7)	158	2018	Bioscience Reports	N	N	Y	N	6	7	Newcastle–Ottawa scale	Y
139	Genetics A4	Shu et al (3)	159	2019	Br J Oral Maxillofac Surg.	N	N	Y	N	3	34	Newcastle-Ottawa scale	Y
140	Genetics A4	Zhou et al (7)	160	2020	Birth Defects Research	N	Y	Y	Y	4	56	Newcastle-Ottawa scale and Cochrane's risk of bias tool.	Y
**Domain. G. RESEARCH METHODS**													
**Sub-domain I - Clinical practice guidelines and Core outcome sets G1**													
N os.	*Subdomain II and code*	*Author and number of reviewers (n)*	*Reference number*	*Year*	*Journal*	*Language limit*	*Time limitation*	*Hand/ Reference search*	*Grey literature*	*Number of databases used*	*Number of Included studies*	*Risk of Bias Tool Used/ quality assessment*	*Meta-analysis*
141	Oral health aspects including dental caries and other dental problems D6	Saikia et al (10)	161	2021	Cleft Palate Craniofac J	N	N	Y	Y	6	7	AGREE II	N
**Sub-domain I - Methodological aspects G3**													

Annexure 4: Detailed characteristics of included studies as Domain, Subdomain I and code, Subdomain II and code (refer to Figure 1 and Table 1 for subdomains and codes), Author and number of reviewers, reference number (as numbered in the reference list of paper), year, journal of publication, language limits, Time limitation, hand/reference searching, grey literature search, number of databases used, number of included studies, Risk of bias tool used for quality assessment and meta-analysis. (Abbreviation Nos.= Numbers, Y= Present, N= Not present). Color codes have been done for different Domains and subdomains (I and II) are as presented in Table 1.

N os.	*Subdomain II and code*	*Author and number of reviewers (n)*	*Reference number*	*Year*	*Journal*	*Language limit*	*Time limitation*	*Hand/ Reference search*	*Grey literature*	*Number of databases used*	*Number of Included studies*	*Risk of Bias Tool Used/ quality assessment*	*Meta-analysis*
142	N/A	Hardwicke et al (3)	162	2017	Cleft Palate Craniofac J	Y	Y	N	N	3	65	CONSORT guidelines	N
**Domain. H. RECENT ADVANCES**													
**Sub-domain I - Any recent research area not covered in the above categorization H1**													
N os.	*Subdomain II and code*	*Author and number of reviewers (n)*	*Reference number*	*Year*	*Journal*	*Language limit*	*Time limitation*	*Hand/ Reference search*	*Grey literature*	*Number of databases used*	*Number of Included studies*	*Risk of Bias Tool Used/ quality assessment*	*Meta-analysis*
143	Alveolar bone grafting and graft materials C10	Shanbhag et al (6)	163	2019	J Clin Periodontol	N	N	Y	Y	3	77	Cochrane	Y
144	Genetics A4	Wei et al (6)	164	2022	Archives of Oral Biology	Y	N	N	N	4	16	Newcastle–Ottawa Scale	N
