# Supplementary material for: Evidence Mapping and Quality Analysis of Systematic Reviews on Various Aspects Related to Cleft Lip and Palate
Source: J Clin Med. 2023 Sep 16;12(18):6002. doi: 10.3390/jcm12186002 (PMC10532364; doi:10.3390/jcm12186002)
Supplement: Supplementary file 1 [file jcm-12-06002-s001.zip › Annexure 5.pdf]

Annexure 5: Detailed characteristics of the included studies including Domain, Subdomain I and code, Subdomain II and code (refer to Figure 1 and Table 1 for subdomains and codes), author, reference number (as numbered in the reference list of paper) year, aims and objectives in P (Population) I/E (Intervention/exposure) C (Comparison) O (Outcome), and grade of conclusion. Color codes have been done for different Domains and subdomains (I and II) are as presented in Table 1. Abbreviations: CLP (cleft lip and/ or cleft palate), CL (cleft lip), CP (cleft palate), CLA (Cleft lip alveolus), UCLP (Unilateral cleft lip and palate), BCLP (bilateral cleft lip and palate). Refer to main text of the systematic review using reference number (3rd column).

**Domain. A. EPIDEMIOLOGY**						
Sub-domain I - Incidence and Prevalence A1						
Nos	*Subdomain II and code*	*Author*	*Reference number*	*Year*	*Aim and objectives in PICO : Population (P), Intervention (I), Comparision (C), Outcome (O)*	*Grade of Conclusion*
1	Anatomical and functional characteristics A6	Tannure et al	21	2012	P= Non-syndromic Individuals With CLP,<br>E= CLP<br>C= None<br>O= Prevalence of dental anomalies	Partially conclusive
2	Anatomical and functional characteristics A6	Khalaf et al	22	2014	P= Patients with CLP, patients with systematic conditions, patients with a family history and race<br>E= Hypodontia<br>C= None<br>O= Prevalence of hypodontia, excluding third molars, Prevalence of hypodontia by population type, continent, gender, type of malocclusion, number of missing teeth, type of missing teeth, upper and lower jaws, geographic region	Inconclusive
3	Embryologic development A3	Allagh et al	23	2015	P= Live births and still birth in community or hospitals;<br>E = Orofacial cleft and neural tube defects<br>C= None.<br>O= Birth prevalence of orofacial clefts and neural tube defects in India	Partially conclusive
4	N/A	Wang et al	24	2017	P= Oral clefts<br>I= Prevalence<br>C= None<br>O= Prevalence of oral clefts	Conclusive
5	Etiopathogenesis A2	Chen et al	25	2018	P=Pregnant women with singleton pregnancies,<br>I=In vitro fertilization/intracytoplasmic sperm injection C=Spontaneously conceived singleton pregnancies<br>O=Birth prevalence of congenital malformations	Partially conclusive
6	N/A	Haseli et al	26	2018	P= Children born in Iran<br>E= CLP<br>C= None<br>O= Prevalence	Partially conclusive
7	Genetics A4	Pereira et al	27	2019	P= A ancestry/group of people;<br>I= Genetic testing using ancestry informational markers,<br>C= None<br>O= Outcome: Brazilian ancestry used to construct the research question and search for evidence	Partially conclusive
8	N/A	Salari et al	28	2021	P= CP, CL and CLP<br>I= Global prevalence	Conclusive

Annexure 5: Detailed characteristics of the included studies including Domain, Subdomain I and code, Subdomain II and code (refer to Figure 1 and Table 1 for subdomains and codes), author, reference number (as numbered in the reference list of paper) year, aims and objectives in P (Population) I/E (Intervention/exposure) C (Comparison) O (Outcome), and grade of conclusion. Color codes have been done for different Domains and subdomains (I and II) are as presented in Table 1. Abbreviations: CLP (cleft lip and/ or cleft palate), CL (cleft lip), CP (cleft palate), CLA (Cleft lip alveolus), UCLP (Unilateral cleft lip and palate), BCLP (bilateral cleft lip and palate). Refer to main text of the systematic review using reference number (3rd column).

					C= None<br>O= Global prevalence	
Sub-domain I - Etiopathogenesis A2						
Nos.	*Subdomain II and code*	*Author*	*Reference number*	*Year*	*Aim and objectives in PICO: Population (P), Intervention (I), Comparison (C), Outcome (O)*	*Grade of Conclusion*
9	Embryologic development A3	Chi et al	29	2010	P= Pregnant women,<br>I= Topical corticosteroids during pregnancy<br>C= Control group<br>O= Pregnancy outcomes	Partially conclusive
10	Embryologic development A3	Dreier et al	30	2013	P= Pregnant women<br>E= Fever<br>C= None<br>O= Health Impacts in the Offspring	Partially conclusive
11	Embryologic development A3	Molina-Solana et al	31	2013	P= Pregnant women who gave birth<br>I= Pregnant women exposed to tobacco, alcohol, fever, folic acid and zinc intake, stress, obesity<br>C= None<br>O= Offspring with CLP	Partially conclusive
12	Risk assessment and prediction F2	Popoff et al	32	2013	P=Relatives of individuals with CLP/ Non-syndromic patients with oral clefts<br>E=Oral cancer<br>C=Family history of cancer/family history of CLP<br>O= Prevalence of cancer	Inconclusive
13	Embryologic development A3	Bell et al	33	2014	P= Pregnant women<br>E= Alcohol consumption<br>C= Control group<br>O= Risk of CLP	Partially conclusive
14	Embryologic development A3	Luteijn et al	34	2014	P= Women in the First trimester<br>E= Maternal influenza<br>C= Control group<br>O= Risk of congenital anomalies	Partially conclusive
15	Genetics A4	Sabbagh et al	35	2014	P= Individuals born to consanguineous parents<br>E= Parental consanguinity<br>C= No parental consanguinity<br>O= Non-syndromic orofacial clefts	Conclusive
16	Embryologic development A3	De-Regil et al	36	2015	P= All women who become pregnant or were 12 or fewer weeks' pregnant at the time of the intervention<br>I=Supplementation with folate<br>C= None<br>O= Birth defects	Conclusive

Annexure 5: Detailed characteristics of the included studies including Domain, Subdomain I and code, Subdomain II and code (refer to Figure 1 and Table 1 for subdomains and codes), author, reference number (as numbered in the reference list of paper) year, aims and objectives in P (Population) I/E (Intervention/exposure) C (Comparison) O (Outcome), and grade of conclusion. Color codes have been done for different Domains and subdomains (I and II) are as presented in Table 1. Abbreviations: CLP (cleft lip and/ or cleft palate), CL (cleft lip), CP (cleft palate), CLA (Cleft lip alveolus), UCLP (Unilateral cleft lip and palate), BCLP (bilateral cleft lip and palate). Refer to main text of the systematic review using reference number (3rd column).

17	Embryologic development A3	Izedonmwen et al	37	2015	P= Pre-Maternal Obese/Overweight Women<br>I= WHO recommended BMI<br>C= Pre-Maternal Normal Weight Women<br>O= Risk of Having Offspring with CLP	Conclusive
18	Embryologic development A3	Sabbagh et al	38	2015	P= Pregnant women<br>E= Passive smoking<br>C= Association with active smoking<br>O= CLP in infants	Conclusive
19	Embryologic development A3	Tanoshima et al	39	2015	P= Pregnant women with epilepsy<br>E= Valproic Acid<br>C= Other AED<br>O= Risks of Congenital Malformations	Conclusive
20	Embryologic development A3	Rao et al	40	2016	P= Pregnant women<br>E= Air Pollution<br>C= None<br>O= Risk of Orofacial Clefts in Infants	Partially conclusive
21	Embryologic development A3	Veroniki et al	41	2017	P= Pregnant women<br>I= AEDs of as monotherapy or polytherapy of any dose<br>C= Placebo, no AED treatment or other AEDs alone or in combination<br>O=Incidence of major CMs, overall and by specific type (cardiac malformations, hypospadias, CLP, club foot, inguinal hernia, and undescended testes)	Conclusive
22	Embryologic development A3	Liang et al	42	2018	P= Singleton pregnancies<br>I= In vitro fertilization/intracytoplasmic sperm injection<br>C= Normal controls<br>O= Congenital malformations	Conclusive
23	Embryologic development A3	Jayarajan et al	43	2019	P= Females of reproductive age group,<br>I= High-dose folic acid<br>C= No supplementation or low-dose folic acid supplementation,<br>O= Occurrence or recurrence of CL ±CP or isolated CP.	Partially conclusive
24	Embryologic development A3	Yin et al	44	2019	P= Pregnant women<br>E= Alcohol consumption<br>C= Control group (women without alcohol consumption)<br>O= CLP	Partially conclusive
25	Embryologic development A3	Picot et al	45	2020	P= First trimester of pregnancy, and the risk of MCM, cardiac defects and orofacial clefts<br>I= ondansetron<br>C= None<br>O= malformations	Partially conclusive
26	Embryologic development A3	Fell et al	46	2021	P= Pregnant women or women who have given birth to live-born children in the general population,	Partially conclusive

Annexure 5: Detailed characteristics of the included studies including Domain, Subdomain I and code, Subdomain II and code (refer to Figure 1 and Table 1 for subdomains and codes), author, reference number (as numbered in the reference list of paper) year, aims and objectives in P (Population) I/E (Intervention/exposure) C (Comparison) O (Outcome), and grade of conclusion. Color codes have been done for different Domains and subdomains (I and II) are as presented in Table 1. Abbreviations: CLP (cleft lip and/ or cleft palate), CL (cleft lip), CP (cleft palate), CLA (Cleft lip alveolus), UCLP (Unilateral cleft lip and palate), BCLP (bilateral cleft lip and palate). Refer to main text of the systematic review using reference number (3rd column).

					E= Active cigarette smoking in women during pregnancy measured either by self-reporting or by proxy measurements<br>C= None<br>O= children born with an orofacial cleft.	
27	Health economics E4	Kruppa et al	47	2021	P= Patients with CL/P, I= lower-middle income, low-middle-income, or low income country,<br>I= None<br>C= Patients in high-income countries<br>O= Biological risks or environmental risks	Conclusive
28	Embryologic development A3	Shi et al	48	2021	P= Pregnant women<br>E= Common cold, fever<br>C= None<br>O= risk of oral clefts in the offspring	Partially conclusive
29	Psychosocial aspects, perceptions and quality of life of patients with CLP E1	Tran et al	49	2022	P= Women in preconception period<br>E= Stressful life events<br>C= No stressful life event in preconception period<br>O= Baby with CLP	Partially conclusive
Sub-domain I - Genetics A4						
Nos.	*Subdomain II and code*	*Author*	*Reference number*	*Year*	*Aim and objectives in PICO: Population (P), Intervention (I), Comparison (C), Outcome (O)*	*Grade of Conclusion*
30	Risk assessment and prediction F2	Machado et al	50	2018	P= Patients with NOC in the Brazilian population,<br>I= Analysis of genetic markers,<br>C= Inclusion of unaffected individuals (unrelated subjects for case-control studies and family members for family-based studies),<br>O= Frequency and potential association of genetic markers, S: observational studies (case-control or family-based approaches).	Conclusive
31	Etiopathogenesis A2	Imani et al	51	2019	P= NSCL/P<br>E= rs3758249 and rs4460498 FOXE1 polymorphisms<br>C= Control group without polymorphism of rs3758249 and rs4460498 FOXE1<br>O= Association with NSCL/P	Conclusive
32	Etiopathogenesis A2	Imani et al	52	2019	P=NSCL/P patients with no other systematic disease<br>I= rs13041247 and rs11696257 v-maf musculoaponeurotic fibrosarcoma oncogene homolog B (MAFB) polymorphisms<br>C= Control group<br>O= Association between polymorphism and NSCLP	Conclusive
33	Etiopathogenesis A2	Imani et al	53	2019	P= Non-syndromic CLP<br>E= BHMT rs3797546 and/or rs3733890 polymorphisms<br>C= Healthy controls	Partially conclusive

Annexure 5: Detailed characteristics of the included studies including Domain, Subdomain I and code, Subdomain II and code (refer to Figure 1 and Table 1 for subdomains and codes), author, reference number (as numbered in the reference list of paper) year, aims and objectives in P (Population) I/E (Intervention/exposure) C (Comparison) O (Outcome), and grade of conclusion. Color codes have been done for different Domains and subdomains (I and II) are as presented in Table 1. Abbreviations: CLP (cleft lip and/ or cleft palate), CL (cleft lip), CP (cleft palate), CLA (Cleft lip alveolus), UCLP (Unilateral cleft lip and palate), BCLP (bilateral cleft lip and palate). Refer to main text of the systematic review using reference number (3rd column).

					O= Risk of NSCL/P	
34	Risk assessment and prediction F2	Mendes et al	54	2019	P= CLP<br>I= Biological material (plasma)<br>C= Healthy individuals<br>O= Association of miRNAs to non-syndromic orofacial clefts	Partially conclusive
35	Etiopathogenesis A2	Imani et al	55	2021	P= Non-syndromic CLP<br>E= ABCA4 polymorphisms (rs560426 and rs481931)<br>C= Healthy controls<br>O= Risk of NSCLP	Conclusive
Sub-domain I - Anatomical and functional characteristics A6						
Nos.	*Subdomain II and code*	*Author*	*Reference number*	*Year*	*Aim and objectives in PICO : Population (P), Intervention (I), Comparison (C), Outcome (O)*	*Grade of Conclusion*
36	N/A	Antonarakis et al	56	2013	P= Non-syndromic UCLP patients with permanent dentition<br>I= Assessment of mesiodistal tooth dimensions on the cleft side C= Mesiodistal tooth dimensions on the non-cleft side, control group<br>O= Mesiodistal tooth dimensions in non-syndromic UCLP patients	Conclusive
37	Advanced radiography including 3D imaging and 3D printing B6	Berrocal et al	57	2014	P= Individuals with CLP<br>I= Computed tomography scans or lateral cephalometric radiographs<br>C= Individuals without CLP<br>O= Cervical vertebrae anomalies	Conclusive
38	Advanced radiography including 3D imaging and 3D printing B6	Kuijpers et al	58	2014	P= Patients with Orofacial Clefts,<br>I= Three-dimensional Imaging Methods,<br>C= None<br>O= Quantitative Analysis of Facial Soft Tissues and Skeletal Morphology	Partially conclusive
39	N/A	Boyce et al	59	2017	P= Individuals with non-syndromic submucous CLP<br>I= Methods to assess speech and language characteristics<br>C= None<br>O= Speech and language characteristics	Partially conclusive
40	N/A	Van Dyck et al	60	2019	P= Patients with CLP<br>I= Dental age estimation, determination of dental development and/or tooth eruption<br>C= Patients without CLP<br>O= Difference in dental age, dental development and/or tooth eruption.	Conclusive
41	N/A	Gallego et al	61	2021	P= Children between 2 & 10 years of age, both sexes<br>E= CLP<br>C= None<br>O= Growth, body weight, height, length, body mass index, and body composition	Inconclusive

Annexure 5: Detailed characteristics of the included studies including Domain, Subdomain I and code, Subdomain II and code (refer to Figure 1 and Table 1 for subdomains and codes), author, reference number (as numbered in the reference list of paper) year, aims and objectives in P (Population) I/E (Intervention/exposure) C (Comparison) O (Outcome), and grade of conclusion. Color codes have been done for different Domains and subdomains (I and II) are as presented in Table 1. Abbreviations: CLP (cleft lip and/ or cleft palate), CL (cleft lip), CP (cleft palate), CLA (Cleft lip alveolus), UCLP (Unilateral cleft lip and palate), BCLP (bilateral cleft lip and palate). Refer to main text of the systematic review using reference number (3rd column).

42	Etiopathogenesis A2	Marzouk et al	62	2021	P= Individuals with non-syndromic orofacial clefts<br>E= Dental anomalies<br>C= Individuals without Oral clefts<br>O= Frequency of dental anomalies (DAs)	Conclusive
43	Conventional radiography and 2D imaging B5	Murugan and Kailasam	63	2021	P= Patients with non-syndromic UCLP and BCLP<br>I= Lateral cephalograms or lateral cephalograms derived from CBCTs, wherein the CBA (N-S-Ba) was measured<br>C= None<br>O= Cranial base angle (N-S-Ba) of patients with CLP and non CLP	Conclusive
44	N/A	Southby et al	64	2021	P= Children born with CP aged up to 18 years<br>E= Speech input processing skills<br>C= Speech input processing skills of typically developing controls or a comparison group from another clinical population<br>O= Speech input processing skills of children born with CLP, CP	Inconclusive
45	N/A	Wu et al	65	2021	P= Patients of any sex and ethnicity with CLP.<br>I= Evaluation of skeletal maturity.<br>C= NCLP children.<br>O= Difference in skeletal maturation between patients with CL/P and NCLP children.	Inconclusive
46	Advanced radiography including 3D imaging and 3D printing B6	Srivastav et al	66	2022	P= Individuals of any age group or gender with CLP, whose assessment of maxillary sinus characteristics has been done by using cone beam computed tomography<br>I= Cone Beam Computed Tomographic assessment of the linear and volumetric characteristics of the maxillary sinus<br>C= Individuals without CLP or the non-affected side of the patients with unilateral CLP<br>O= Anatomic hard and soft tissue variations, 2. Linear measurements of the maxillary sinus, 3. Volumetric measurements of the maxillary sinus	Partially conclusive
**Domain. B. DIAGNOSTIC**						
Sub-domain I - Indices of CLP B2						
Nos .	*Subdomain II and code*	*Author*	*Reference number*	*Year*	*Aim and objectives in PICO: Population (P), Intervention (I), Comparision (C), Outcome (O)*	*Grade of Conclusion*
47	Outcome assessment C15	Altalibi et al	67	2013	P= CLP patients<br>I= various indices of CLP assessment<br>C= gradation of dental models of CLP patients<br>O= reliability and validity of index	Conclusive
48	Treatment planning and prediction C1	Buj-Acosta et al	68	2017	P= patients born with unilateral CLP<br>I= Study models (pre-treatment) and GOSLON rating<br>C= Study models (post-treatment) and GOSLON rating	Inconclusive

Annexure 5: Detailed characteristics of the included studies including Domain, Subdomain I and code, Subdomain II and code (refer to Figure 1 and Table 1 for subdomains and codes), author, reference number (as numbered in the reference list of paper) year, aims and objectives in P (Population) I/E (Intervention/exposure) C (Comparison) O (Outcome), and grade of conclusion. Color codes have been done for different Domains and subdomains (I and II) are as presented in Table 1. Abbreviations: CLP (cleft lip and/ or cleft palate), CL (cleft lip), CP (cleft palate), CLA (Cleft lip alveolus), UCLP (Unilateral cleft lip and palate), BCLP (bilateral cleft lip and palate). Refer to main text of the systematic review using reference number (3rd column).

					O= Predictive validity of the GOSLON Yardstick index	
Sub-domain I - Prenatal diagnosis B3						
Nos.	*Subdomain II and code*	*Author*	*Reference number*	*Year*	*Aim and objectives in PICO: Population (P), Intervention (I), Comparison (C), Outcome (O)*	*Grade of Conclusion*
49	Conventional radiography and 2D imaging B5	Lai et al	69	2021	P= second and third trimester (12–36 weeks) of pregnancy<br>I= prenatal fetal ultrasound<br>C= None<br>O= prenatal detection of CLP (diagnostic accuracy)	Conclusive
Sub-domain I - Advanced radiography including 3D imaging and 3D printing B6						
Nos.	*Subdomain II and code*	*Author*	*Reference number*	*Year*	*Aim and objectives in PICO: Population (P), Intervention (I), Comparison (C), Outcome (O)*	*Grade of Conclusion*
50	Clinical practice guidelines and Core outcome sets G1	van Vlijmen et al	70	2012	P= Orthodontic patients<br>I= CBCT<br>C= None<br>O= Applications of CBCT in orthodontics	Partially conclusive
51	Anatomical and functional characteristics A6	Thierens et al	71	2018	P= CLP patients before intervention<br>I= 3D imaging<br>C= CLP patients after orthopedic or surgical intervention<br>O= soft tissue changes	Partially conclusive
52	Anatomical and functional characteristics A6	Thierens et al	72	2018	P= Patients with repaired or unrepaired CLP<br>I= non-ionizing 3D images acquired with 3D surface scanning or 3D digital stereophotogrammetry<br>C= None<br>O= quantification of facial morphology, symmetry, and appearance	Conclusive
**Domain. C. THERAPEUTIC**						
Sub-domain I - Feeding and nutrition in patients C2						
Nos.	*Subdomain II and code*	*Author*	*Reference number*	*Year*	*Aim and objectives in PICO: Population (P), Intervention (I), Comparision (C), Outcome (O)*	*Grade of Conclusion*
53	Clinical practice guidelines and Core outcome sets G1	Mei et al	73	2009	P= breastfed infants (0 to 6 months) with CL/P<br>I= breastfeeding<br>C= None<br>O= outcome measures of interest—suction and intra-oral pressure, attachment to breast, growth (weight and length), supplemental feeding, feeding duration, and mother satisfaction with advice received	Conclusive
Sub-domain I - Presurgical Infant Orthopaedics and Naso-Alveolar Moulding C3						
Nos.	*Subdomain II and code*	*Author*	*Reference number*	*Year*	*Aim and objectives in PICO: Population (P), Intervention (I), Comparison (C), Outcome (O)*	*Grade of Conclusion*

Annexure 5: Detailed characteristics of the included studies including Domain, Subdomain I and code, Subdomain II and code (refer to Figure 1 and Table 1 for subdomains and codes), author, reference number (as numbered in the reference list of paper) year, aims and objectives in P (Population) I/E (Intervention/exposure) C (Comparison) O (Outcome), and grade of conclusion. Color codes have been done for different Domains and subdomains (I and II) are as presented in Table 1. Abbreviations: CLP (cleft lip and/ or cleft palate), CL (cleft lip), CP (cleft palate), CLA (Cleft lip alveolus), UCLP (Unilateral cleft lip and palate), BCLP (bilateral cleft lip and palate). Refer to main text of the systematic review using reference number (3rd column).

54	Cleft Lip repair C8	Heijden et al	74	2012	P= non-syndromic unilateral CL, jaw, and palate<br>I= naso-alveolar molding plus lip repair<br>C= control group with Lip repair only<br>O= nasal symmetry after PSIO	Inconclusive
55	Outcome assessment C15	Namdar et al	75	2020	P= CLP patients<br>I= Nasoalveolar Molding<br>C= control group<br>O= Positive outcomes with PNAM	Partially conclusive
56	Outcome assessment C15	Padovano et al	76	2021	P= children with UCLP<br>I= NAM<br>C= non-NAM passive PSIO and/or to no appliance-based presurgical treatment<br>O= effects on nasolabial aesthetic outcomes, craniofacial form, and dental arch development after NAM	Partially conclusive
Sub-domain I - Orthodontic and Surgical Perspectives of Secondary Alveolar Bone Grafting C5						
Nos.	*Subdomain II and code*	*Author*	*Reference number*	*Year*	*Aim and objectives in PICO: Population (P), Intervention (I), Comparison (C), Outcome (O)*	*Grade of Conclusion*
57	Advanced radiography including 3D imaging and 3D printing B6	Mulder et al	77	2019	P= CLP patients<br>I= SABG, 3D radiological evaluation<br>C= Nonne<br>O= types of 3D imaging and success and resorption rates after SABG	Inconclusive
58	Advanced radiography including 3D imaging and 3D printing B6	Scalzone et al	78	2019	P= unilateral CLP older than 5 years of age<br>I= radiographic evaluation (CT and/or CBCT) of the cleft area<br>C= traditional iliac crest graft with secondary bone graft using alloplastic material<br>O= radiographic assessment of bone graft volume through the 3D images and the radiographic assessment in the grafted area of alveolar bone height, Length of hospital stay	Conclusive
59	Alveolar bone grafting and graft materials C10	Pinheiro et al	79	2020	P= Subjects with cleft from all ethnical backgrounds with either unilateral or bilateral clefts<br>I= Early and/or conventional secondary alveolar bone grafts using autogenous illiac bone<br>C= Autogenous conventional secondary alveolar bone graft,<br>O= Success rate, bone height, bone volume, tooth eruption, and periodontal status of cleft-adjacent teeth; minimum follow-up time of 6 months in average	Inconclusive
60	Outcome assessment C15	Yu et al	80	2020	P= Patients with unilateral or bilateral non-syndromic CLA or non-syndromic CLP<br>I= secondary alveolar bone grafting, 2D and 3D radiographs<br>C= None<br>O= postoperative change in the cleft defect	Partially conclusive

Annexure 5: Detailed characteristics of the included studies including Domain, Subdomain I and code, Subdomain II and code (refer to Figure 1 and Table 1 for subdomains and codes), author, reference number (as numbered in the reference list of paper) year, aims and objectives in P (Population) I/E (Intervention/exposure) C (Comparison) O (Outcome), and grade of conclusion. Color codes have been done for different Domains and subdomains (I and II) are as presented in Table 1. Abbreviations: CLP (cleft lip and/ or cleft palate), CL (cleft lip), CP (cleft palate), CLA (Cleft lip alveolus), UCLP (Unilateral cleft lip and palate), BCLP (bilateral cleft lip and palate). Refer to main text of the systematic review using reference number (3rd column).

61	Alveolar bone grafting and graft materials C10	Jahanbin et al	81	2021	P= Patients with unilateral or bilateral CLP<br>I= secondary alveolar bone grafting<br>C= patients before or without graft and other methods were performed for grafting<br>O= long term success of the alveolar bone graft.	Partially Conclusive
62	Outcome assessment C15	Lacerda-Santos et al	82	2021	P= Patients with CLP, and mean age between 7 and 14 years<br>I= Secondary alveolar graft surgery in the cleft region<br>C= Not submitted to surgery<br>O= Increased rate of canine eruption, Successful bone graft. Canine impacted due to agenesis of the lateral incisor. Effect of orthodontic treatment before and after bone graft surgery	Inconclusive
Sub-domain I - Conventional orthodontics C6						
Nos.	*Subdomain II and code*	*Author*	*Reference number*	*Year*	*Aim and objectives in PICO: Population (P), Intervention (I), Comparison (C), Outcome (O)*	*Grade of Conclusion*
63	Outcome assessment C15	Palikaraki et al	83	2020	P= Individuals of any age, gender, and ethnicity with UCLP<br>I= Studies with at least one group of UCLP patients treated with a conventional facemask with or without expansion<br>C= It should be a human clinical trial that had a comparable untreated control group of patients with or without UCLP<br>O= Angular and linear cephalometric measurements evaluated by cephalometric analysis before and after treatment.	Partially conclusive
64	Outcome assessment C15	Shafaee et al	84	2020	P= CLP patients<br>I = Face mask therapy, 2D and 3D evaluation<br>C= non-cleft patients<br>O= dimensions of the airway space and relevant landmarks in cephalometric and 3D images after Face mask therapy	Conclusive
Sub-domain I - Cleft Lip repair C8						
Nos.	*Subdomain II and code*	*Author*	*Reference number*	*Year*	*Aim and objectives in PICO: Population (P), Intervention (I), Comparison (C), Outcome (O)*	*Grade of Conclusion*
65	Effects/Complications of PSIO and PNAM D2	Thierens et al	85	2016	P= CLP<br>I= Labial adhesion<br>C= None<br>O= Effect on maxillary arch dimensions and nasolabial esthetics	Inconclusive
66	Outcome assessment C15	Jayarajan et al	86	2018	P= Patients with non-syndromic unilateral CL undergoing rhinoplasty along with primary CL repair<br>I= Use of open rhinoplasty<br>C= Use of closed rhinoplasty technique<br>O= Definitive anatomical measurements of the nose parameters during follow-up after CL repair	Inconclusive
Sub-domain I - Cleft alveolus, sub-mucous cleft and all types of cleft palate closure/repair C9						
Nos.	*Subdomain II and code*	*Author*	*Reference number*	*Year*	*Aim and objectives in PICO: Population (P), Intervention (I), Comparison (C), Outcome (O)*	*Grade of Conclusion*

Annexure 5: Detailed characteristics of the included studies including Domain, Subdomain I and code, Subdomain II and code (refer to Figure 1 and Table 1 for subdomains and codes), author, reference number (as numbered in the reference list of paper) year, aims and objectives in P (Population) I/E (Intervention/exposure) C (Comparison) O (Outcome), and grade of conclusion. Color codes have been done for different Domains and subdomains (I and II) are as presented in Table 1. Abbreviations: CLP (cleft lip and/ or cleft palate), CL (cleft lip), CP (cleft palate), CLA (Cleft lip alveolus), UCLP (Unilateral cleft lip and palate), BCLP (bilateral cleft lip and palate). Refer to main text of the systematic review using reference number (3rd column).

67	Effects/Complications of Surgical treatment and/or alveolar bone grafting D4	Salgado et al	87	2019	P= Unilateral complete CLP<br>I= Early palatoplasty, with hard palate surgery carried out before 18 months<br>C= children subjected to late palatoplasty, with hard palate surgery carried out after 18 months<br>O= assessing through cephalometric radiographs in lateral norm, the differences in the maxillary growth among the groups	Inconclusive
Sub-domain I - Alveolar bone grafting and graft materials C10						
Nos.	*Subdomain II and code*	*Author*	*Reference number*	*Year*	*Aim and objectives in PICO: Population (P), Intervention (I), Comparison (C), Outcome (O)*	*Grade of Conclusion*
68	Orthodontic and Surgical Perspectives of Secondary Alveolar Bone Grafting C5	Guo et al	88	2011	P= Participants have CLP<br>I= Secondary alveolar bone grafting<br>C= Variations in timing, donor sites, bone substitutes and technique<br>O= Bone graft healing (Clinical and radiographic assessment)	Inconclusive
69	Orthodontic and Surgical Perspectives of Secondary Alveolar Bone Grafting C5	Canellas et al	89	2018	P= Patients requiring various oral surgical procedures<br>I= Use of autologous PRF combined or not with another biomaterial<br>C= Comparator or control group: no treatment or another biomaterial combination without PRF<br>O= Success of the procedure, S= studies in humans, including only randomized control trials	Partially conclusive
70	Orthodontic and Surgical Perspectives of Secondary Alveolar Bone Grafting C5	da Rosa et al	90	2018	P= Patients with CLP defects<br>I= Use of rhBMP-2 in biomaterials for bone graft<br>C= Use of iliac crest bone graft (ICBG)<br>O= Bone formation volume and bone filling percentage	Conclusive
71	Advanced radiography including 3D imaging and 3D printing B6	Kamal et al	91	2018	P= Patients with congenital CLP with alveolar cleft defect<br>I= Grafting surgical interventions (autogenous or tissue-engineered bone substitutes), volumetric imaging<br>C= None<br>O= Effectiveness (filling the alveolar cleft defect)	Conclusive
72	Effects/Complications of Surgical treatment and/or alveolar bone grafting D4	Simpson et al	92	2018	P= Patients undergoing primary cleft palate or oronasal fistula repair<br>I= Use of ADM during surgical repair<br>C= None<br>O= Postoperative fistula rate	Inconclusive
73	Comprehensive Interdisciplinary care C13	Sales et al	93	2019	P= Cleft Patients<br>I= Dental implants placed in bone graft area<br>C= None<br>O= Effectiveness	Partially conclusive

Annexure 5: Detailed characteristics of the included studies including Domain, Subdomain I and code, Subdomain II and code (refer to Figure 1 and Table 1 for subdomains and codes), author, reference number (as numbered in the reference list of paper) year, aims and objectives in P (Population) I/E (Intervention/exposure) C (Comparison) O (Outcome), and grade of conclusion. Color codes have been done for different Domains and subdomains (I and II) are as presented in Table 1. Abbreviations: CLP (cleft lip and/ or cleft palate), CL (cleft lip), CP (cleft palate), CLA (Cleft lip alveolus), UCLP (Unilateral cleft lip and palate), BCLP (bilateral cleft lip and palate). Refer to main text of the systematic review using reference number (3rd column).

74	Cleft alveolus, submucous cleft and all types of cleft palate closure/repair C9	Uribe et al	94	2019	P= Patients with CLA<br>I= Reconstruction of the alveolar cleft with rhBMP-2.<br>C= Iliac crest bone graft<br>O= Average bone volume formation and average bone height formation in the alveolar cleft. Mean difference<br>was calculated and pooled by meta-analysis.	Partially conclusive
75	Orthodontic and Surgical Perspectives of Secondary Alveolar Bone Grafting C5	Francisco et al	95	2020	P= Orthodontic patients of any gender or age<br>I= PRF with/without a combined biomaterial<br>C= The control group- participants that underwent treatments approaches without PRF<br>O= Hard tissue reconstruction of alveolar bone,rate of tooth movement	Partially conclusive
76	Presurgical Infant Orthopaedics and Naso-Alveolar Moulding C3	Ma et al	96	2020	P= CLP patients<br>I= Alveolar bone grafting with presurgical orthodontics<br>C= None<br>O= Bone volume and complications after ABG	Partially conclusive
77	Outcome assessment C15	Osorio et al	97	2020	P= Patients with alveolar clefts<br>I= Synthetic bone substitutes and autogenous bone grafts<br>C= Autogenous bone grafts<br>O= Changes in bone density, volume and height, decrease in the rate of bone resorption and bone formation on imaging	Partially conclusive
78	Outcome assessment C15	Xiao et al	98	2020	P= Alveolar cleft patients<br>I= Bone morphogenetic protein-2(BMP-2)<br>C= Iliac cancellous bone grafting (IBCG)<br>O= Effect on alveolar cleft bone grafting(ACBG)	Conclusive
79	Orthodontic and Surgical Perspectives of Secondary Alveolar Bone Grafting C5	Alkaabi et al	99	2021	P= CLP patients who underwent treatment of alveolar bone defects<br>I = Alveolar bone grafting using regenerative bone graft<br>C=Iliac crest graft<br>O= New bone formation.	Partially conclusive
80	Outcome assessment C15	Carbullido et al	100	2021	P= Patients who underwent PABG at 24 months of age or younger and a minimum of 5 year follow-up<br>I= Clinical and radiographic evaluation<br>C= Control, SABG/ non-cleft group<br>O= (1) bone graft survival, (2) craniofacial skeletal relationships, (3) occlusion and arch forms, and (4) recommendations for utilizing PABG in practice	Partially conclusive
81	Orthodontic and Surgical Perspectives of Secondary Alveolar Bone Grafting C5	Chetpakdeechit et al	101	2021	P= CLP patients,<br>I= Surgical technique, grafting material,<br>parameters<br>C= None	Inconclusive

Annexure 5: Detailed characteristics of the included studies including Domain, Subdomain I and code, Subdomain II and code (refer to Figure 1 and Table 1 for subdomains and codes), author, reference number (as numbered in the reference list of paper) year, aims and objectives in P (Population) I/E (Intervention/exposure) C (Comparison) O (Outcome), and grade of conclusion. Color codes have been done for different Domains and subdomains (I and II) are as presented in Table 1. Abbreviations: CLP (cleft lip and/ or cleft palate), CL (cleft lip), CP (cleft palate), CLA (Cleft lip alveolus), UCLP (Unilateral cleft lip and palate), BCLP (bilateral cleft lip and palate). Refer to main text of the systematic review using reference number (3rd column).

					O= Level of the grafted bone, achievement of orthodontic tooth movement into the grafted area, need for re-operation.	
82	Orthodontic and Surgical Perspectives of Secondary Alveolar Bone Grafting C5	Deshpande et al	102	2021	P= Patients undergoing alveolar grafting surgery<br>I= PRP in combination with autogenous iliac bone graft<br>C= Conventional iliac bone graft<br>O= Quantitative bone regeneration	Conclusive
Sub-domain I - Orthognathic and osteogenic distraction surgical orthodontics C11						
Nos.	*Subdomain II and code*	*Author*	*Reference number*	*Year*	*Aim and objectives in PICO : Population (P), Intervention (I), Comparision (C), Outcome (O)*	*Grade of Conclusion*
83	Outcome assessment C15	Saltaji et al	103	2012	P= Patients With CLP<br>I= Maxillary Advancement With Conventional Orthognathic Surgery<br>C= None<br>O= skeletal stability	Conclusive
84	Outcome assessment C15	Saltaji et al	104	2012	P=CLP patients<br>I= Maxillary advancement with distraction osteogenesis<br>C= None<br>O= Long-term skeletal stability	Conclusive
85	Outcome assessment C15	Viwattanatipa et al	105	2015	P= CLP patients<br>I= Orthognathic surgery and distraction osteogenesis<br>C= None<br>O= Mandibular plane change after surgery	Conclusive
86	Outcome assessment C15	Jiang et al	106	2020	P= Non-growing patients with CLP (>14 years of age)<br>I= LFI with rigid fixation, LFIDO, or AMDO was performed to treat maxillary hypoplasia<br>C= None<br>O= Horizontal changes to the maxilla at >6 months after LFI surgery with rigid fixation or after distraction by LFIDO or	Conclusive
Sub-domain I - Other surgical aspects of cleft lip and palate including revisions, scar management, post-operative pain and anaesthesia C12						
Nos.	*Subdomain II and code*	*Author*	*Reference number*	*Year*	*Aim and objectives in PICO: Population (P), Intervention (I), Comparision (C), Outcome (O)*	*Grade of Conclusion*
87	Effects/Complications of Surgical treatment and/or alveolar bone grafting D4	Suresh et al	107	2014	P= Paediatric patients undergoing different surgical procedures I= regional anaesthesia techniques<br>C= None<br>O = Postoperative pain	Partially conclusive
88	Cleft Lip repair C8	Feriani et al	108	2016	P= Children with CL<br>I= Infraorbital nerve block<br>C= Placebo<br>O= Pain score control, Duration of postoperative analgesia, Adverse events, Supplemental analgesic requirements	Inconclusive

Annexure 5: Detailed characteristics of the included studies including Domain, Subdomain I and code, Subdomain II and code (refer to Figure 1 and Table 1 for subdomains and codes), author, reference number (as numbered in the reference list of paper) year, aims and objectives in P (Population) I/E (Intervention/exposure) C (Comparison) O (Outcome), and grade of conclusion. Color codes have been done for different Domains and subdomains (I and II) are as presented in Table 1. Abbreviations: CLP (cleft lip and/ or cleft palate), CL (cleft lip), CP (cleft palate), CLA (Cleft lip alveolus), UCLP (Unilateral cleft lip and palate), BCLP (bilateral cleft lip and palate). Refer to main text of the systematic review using reference number (3rd column).

89	Effects/Complications of Surgical treatment and/or alveolar bone grafting D4	Kendall et al	109	2018	P= Pediatric population<br>I= Regional anesthesia<br>C= Local anesthetics and a control group in pediatric patients undergoing various surgical procedures<br>O= Improvement of postoperative analgesia, complications associated with regional anesthesia	Inconclusive
90	Alveolar bone grafting and graft materials C10	Tache and Mommaerts	110	2019	P= Hospitalized CL and alveolus patients with or without cleft palate undergoing iliac crest bone grafting<br>I= Assessment of intraoperative pain control methods used after alveolar bone grafting<br>C= Evidence<br>O= Duration of hospital stay, subjective pain rating, and degree of impaired mobility.	Inconclusive
91	Cleft alveolus, submucous cleft and all types of cleft palate closure/repair C9	Liu et al	111	2021	P= Patients with CLP repair<br>I= Dexmedetomidine<br>C= Controls (including saline and other drugs)<br>O= Efficaciousness of dexmedetomidine (the primary outcome was the incidence of EA, the secondary outcome was the need for postoperative rescue analgesia and the third outcomes were the incidence of adverse effects)	Partially conclusive
Sub-domain I - Management of speech and hearing problems C14						
Nos.	*Subdomain II and code*	*Author*	*Reference number*	*Year*	*Aim and objectives in PICO: Population (P), Intervention (I), Comparison (C), Outcome (O)*	*Grade of Conclusion*
92	Cleft alveolus, submucous cleft and all types of cleft palate closure/repair C9	Nasser et al	112	2008	P= Children or adolescents, 3 years or older, with an established diagnosis of velopharyngeal insufficiency (VPI) associated with submucous cleft palate<br>I= Surgical procedures, either against each other or no treatment, to correct velopharyngeal insufficiency (VPI) in submucous cleft palate<br>C= None<br>O= Perpetual speech assessment and treatment outcome after surgery	Inconclusive
93	N/A	Lee et al	113	2009	P= Individuals with articulation problems associated with cleft palate<br>I= Electropalatography<br>C= No treatment, delayed treatment, “standard treatment”, or alternative treatment techniques<br>O= Measure of correct articulation of speech sounds targeted in therapy,	Inconclusive
94	N/A	Meinusch and Romonath	114	2011	P= Children with CL and/or palate up to 4;11 years old<br>I= Early language intervention focusing<br>C= None<br>O= Outcomes related to receptive or expressive vocabulary	Inconclusive
95	N/A	Bessell et al	115	2013	P=Children with CP +/- L were included in the review. Studies had to include at least 90% of children with non-syndromic CLP or with no known developmental delay<br>I= Speech language therapy (SLT) intervention,<br>C= No intervention control group or a different SLT intervention were used as comparators,<br>O= Any speech and/or language outcome	Inconclusive

Annexure 5: Detailed characteristics of the included studies including Domain, Subdomain I and code, Subdomain II and code (refer to Figure 1 and Table 1 for subdomains and codes), author, reference number (as numbered in the reference list of paper) year, aims and objectives in P (Population) I/E (Intervention/exposure) C (Comparison) O (Outcome), and grade of conclusion. Color codes have been done for different Domains and subdomains (I and II) are as presented in Table 1. Abbreviations: CLP (cleft lip and/ or cleft palate), CL (cleft lip), CP (cleft palate), CLA (Cleft lip alveolus), UCLP (Unilateral cleft lip and palate), BCLP (bilateral cleft lip and palate). Refer to main text of the systematic review using reference number (3rd column).

96	Cleft alveolus, submucous cleft and all types of cleft palate closure/repair C9	Gilleard et al	116	2014	P= patients with VPI secondary to SMCP<br>I=Various surgical management (Von Langenbeck and pharyngeal flap minimal incision palatopharyngoplasty)<br>C= other surgical methods as control (e.g. incision palatopharyngoplasty combined with either pharyngoplasty or a pharyngeal flap)<br>O= postoperative speech	Inconclusive
97	Effects/Complications of Surgical treatment and/or alveolar bone grafting D4	Kuo et al	117	2014	P=CLP patients<br>I= ventilation tube insertion (Grommet)<br>C= None<br>O= effectiveness, Otitis media	Inconclusive
98	N/A	Lane et al	118	2020	P= children with cleft palate<br>I= early speech interventions<br>C= watchful waiting group, where no direct intervention was provided, or an active, direct therapy group where an alternative intervention was provided<br>O= treatment outcome	Inconclusive
99	Outcome assessment C15	Sand et al	119	2022	P= individuals born with CLP<br>I=Speech-Language Therapy (SLT)<br>C= None<br>O= benefit of SLT (an outcome that is related to speech production, language ability, intelligibility, or PROs)	Partially conclusive
Sub-domain I - Outcome assessment C15						
Nos.	*Subdomain II and code*	*Author*	*Reference number*	*Year*	*Aim and objectives in PICO: Population (P), Intervention (I), Comparision (C), Outcome (O)*	*Grade of Conclusion*
100	Feeding and nutrition in patients C2	Bessell et al	120	2011	P= Babies with CLP<br>I= Various feeding interventions<br>C= unmodified equipment or advice<br>O= growth, development, parental satisfaction	Partially conclusive
101	Orthognathic and osteogenic distraction surgical orthodontics C11	Sharshar et al	121	2012	P= Patients with CLP who have undergone Maxillary Anterior Advancement by Distraction Osteogenesis<br>I= Cephalometric Evaluation<br>C= None<br>O= Upper airway size and nasal resistance	Inconclusive
102	Orthognathic and osteogenic distraction surgical orthodontics C11	Austin et al	122	2015	P= Patients with CLP aged over 16 years with moderate maxillary hypoplasia requiring 4–10 mm of maxillary advancement<br>I= The active intervention of distraction osteogenesis(DO),<br>C= The control intervention of conventional orthognathic surgery (CO)<br>O= 1. Maxillary correction achieved with the two interventions, 2. Post-operative skeletal stability of the maxilla, 3. Speech and velopharyngeal function, 4. Psychological adjustment and quality of life, 5.	Inconclusive

Annexure 5: Detailed characteristics of the included studies including Domain, Subdomain I and code, Subdomain II and code (refer to Figure 1 and Table 1 for subdomains and codes), author, reference number (as numbered in the reference list of paper) year, aims and objectives in P (Population) I/E (Intervention/exposure) C (Comparison) O (Outcome), and grade of conclusion. Color codes have been done for different Domains and subdomains (I and II) are as presented in Table 1. Abbreviations: CLP (cleft lip and/ or cleft palate), CL (cleft lip), CP (cleft palate), CLA (Cleft lip alveolus), UCLP (Unilateral cleft lip and palate), BCLP (bilateral cleft lip and palate). Refer to main text of the systematic review using reference number (3rd column).

					Outcomes relating to harms, risks and benefits.	
103	Psychosocial aspects, perceptions and quality of life of patients with CLP E2	Norman et al	123	2015	P= Non-syndromic CL/P with or without cleft alveolus or individuals with syndromes with no known developmental delay, I= All interventions addressing psychosocial adjustment<br>C= No intervention or that compared either different psychosocial interventions or psychosocial interventions at different points<br>O= Any outcome measures related to psychosocial functioning	Inconclusive
104	Presurgical Infant Orthopaedics and Naso-Alveolar Moulding C3	Hosseini et al	124	2017	P= Children of any age with any kind of non-syndromic CLP defect<br>I= Any type of PSIO appliance<br>C= No treatment or an alternative PSIO protocol<br>O= Treatment outcome (effectiveness)	Partially conclusive
105	Cleft alveolus, submucous cleft and all types of cleft palate closure/repair C9	El-Ashmawi et al	125	2018	P=CLP (Unilateral or bilateral)<br>I=Patients who had only GPP done for alveolar bone reconstruction (primary or secondary) with or without adjunctive PSIO<br>C= Patients without GPP, may comprise patients with CLP evaluated before age of bone graft (during early childhood, Patients with CLP evaluated during late childhood and adolescence, ie, after age of bone graft (SBG group)<br>O= 1: Alveolar bone quality, 2: Facial growth	Inconclusive
106	Orthognathic and osteogenic distraction surgical orthodontics C11	Kloukos et al	126	2018	P= Cleft patients<br>I= Maxillary distraction osteogenesis<br>C= Orthognathic surgery<br>O= Maxillary advancement, Long-term skeletal relapse, Speech, Velopharyngeal function, Psychological status	Inconclusive
107	Advanced radiography including 3D imaging and 3D printing B6	Awarun et al	127	2019	P= Cleft patients after cleft-related treatment during growth<br>I=Three-dimensional (3D) imaging methods<br>C=None<br>O= Quantification of the changes in soft- and hard tissues in cleft patients after cleft-related treatment during growth	Inconclusive
108	Indices of CLP B2	Kuang et al	128	2021	P= BCLP<br>I= Huddart-Bodenham Scoring System and the BCLP Yardstick<br>C= None<br>O= Treatment Outcome	Inconclusive
109	Orthognathic and osteogenic distraction surgical orthodontics C11	Nimbalkar et al	129	2021	P= Adult/non-growing CLP patients (15–30 years)<br>I= Maxillary advancement with DO using RED device<br>C= Young/growing CLP patients (7–14.9 years) O= Horizontal changes at the maxilla at >12 months after DO using RED.<br>The measurement of change at point A on the horizontal reference line using an x-y coordinate system.	Conclusive

Annexure 5: Detailed characteristics of the included studies including Domain, Subdomain I and code, Subdomain II and code (refer to Figure 1 and Table 1 for subdomains and codes), author, reference number (as numbered in the reference list of paper) year, aims and objectives in P (Population) I/E (Intervention/exposure) C (Comparison) O (Outcome), and grade of conclusion. Color codes have been done for different Domains and subdomains (I and II) are as presented in Table 1. Abbreviations: CLP (cleft lip and/ or cleft palate), CL (cleft lip), CP (cleft palate), CLA (Cleft lip alveolus), UCLP (Unilateral cleft lip and palate), BCLP (bilateral cleft lip and palate). Refer to main text of the systematic review using reference number (3rd column).

110	Presurgical Infant Orthopaedics and Naso-Alveolar Moulding C3	Sazo et al	130	2021	P= Patients with CLP<br>I= Presurgical orthopedics<br>C= None<br>O= Effect on esthetics, feeding, and the gap between maxillary segments.	Conclusive
**Domain. D. PROGNOSTIC**						
Sub-domain I - Effects/Complications of Surgical treatment and/or alveolar bone grafting D4						
Nos.	*Subdomain II and code*	*Author*	*Reference number*	*Year*	*Aim and objectives in PICO: Population (P), Intervention (I), Comparison (C), Outcome (O)*	*Grade of Conclusion*
111	Cleft alveolus, submucous cleft and all types of cleft palate closure/repair C9	Hardwicke et al	131	2014	P= Human participants with non-syndromic cleft palate who underwent primary cleft palate repair<br>I= Primary repair of cleft palate; all ages at intervention; all surgical repair techniques<br>C=None<br>O= Number of reported oronasal fistulae as a proportion of the entire cohort	Conclusive
112	Cleft alveolus, submucous cleft and all types of cleft palate closure/repair C9	Felton et al	132	2017	P= Infants with CLP<br>I= Early ventilation tube placement prior to palatoplasty<br>C= CL repair only (control group)<br>O= Prevalence of OME preoperatively, Complications of ventilation tubes, ventilation tube function/patency, hearing loss (conductive) and speech development	Partially conclusive
113	Cleft alveolus, submucous cleft and all types of cleft palate closure/repair C9	Kappen et al	133	2018	P= Patients with unilateral CLP<br>I/E= Gender, the timing of hard palate closure, soft palate closure, the alveolar bone graft, surgical approach (one-stage vs. two-stage palatoplasty), the incidence of fistulas, the incidence of pharyngoplasty<br>C= None<br>O= Influence of treatment factors on long-term maxillary growth	Inconclusive
114	Orthognathic and osteogenic distraction surgical orthodontics C11	Liu et al	134	2018	P= Growing Children with CLP<br>I= Maxillary Distraction Osteogenesis<br>C= None<br>O= Long-Term Skeletal Changes	Conclusive
115	Orthognathic and osteogenic distraction surgical orthodontics C11	Dos Santos Alves et al	135	2019	P= Individuals submitted to Le Fort I osteotomy<br>I= Method to assess cranial nerve function<br>C= None<br>O= Signs and symptoms associated with cranial nerve injuries	Partially conclusive
116	Cleft alveolus, submucous cleft and all types of cleft palate closure/repair C9	Minatel et al	136	2019	P= Non-syndromic patients with CLP<br>I= Patients who underwent vomer flap for correction of cleft in the hard palate<br>C= Patients who underwent other surgical techniques for correction of cleft in the hard palate<br>O= Craniofacial growth (primary outcome); speech development and occurrence of fistula (secondary outcomes).	Conclusive

Annexure 5: Detailed characteristics of the included studies including Domain, Subdomain I and code, Subdomain II and code (refer to Figure 1 and Table 1 for subdomains and codes), author, reference number (as numbered in the reference list of paper) year, aims and objectives in P (Population) I/E (Intervention/exposure) C (Comparison) O (Outcome), and grade of conclusion. Color codes have been done for different Domains and subdomains (I and II) are as presented in Table 1. Abbreviations: CLP (cleft lip and/ or cleft palate), CL (cleft lip), CP (cleft palate), CLA (Cleft lip alveolus), UCLP (Unilateral cleft lip and palate), BCLP (bilateral cleft lip and palate). Refer to main text of the systematic review using reference number (3rd column).

117	Cleft alveolus, submucous cleft and all types of cleft palate closure/repair C9	Tache and Mommaerts	137	2019	P= Non-syndromic CLP patients who underwent primary cleft surgery<br>I= Technique and timing of the primary surgery and type of cleft<br>C= Secondary CLP repair<br>O= Occurrence of the oronasal fistula	Conclusive
118	Conventional radiography and 2D imaging B4	Corthouts et al	138	2020	P= Non-syndromic UCLP patient population over 6 years old who underwent various surgical protocols<br>I= Cephalometric evaluation<br>C=Control or comparison group<br>O= Maxillofacial growth	Partially conclusive
119	Other surgical aspects of cleft lip and palate including revisions, scar management, post-operative pain and anaesthesia C12	Miranda et al	139	2020	P= CLP patients following primary surgery<br>I= Fistula treatment<br>C= None<br>O= Fistula closure, recurrence of fistulas, residual fistulas, suture dehiscence, postoperative infections, failures in healing	Partially conclusive
120	Cleft alveolus, submucous cleft and all types of cleft palate closure/repair C9	Vandenberg et al	140	2020	P= patients with CLP<br>I= Vomer Flap Repair<br>C= None<br>O= incidence of oronasal fistulas	Conclusive
121	Orthognathic and osteogenic distraction surgical orthodontics C11	Velasques et al	141	2020	P= CLP patients<br>I= Le Fort 1 osteotomy<br>C= None<br>O= Maxillary relapse	Partially conclusive
122	Cleft Lip repair C8	Egbunah et al	142	2021	P= Patients with CL undergoing surgical repair<br>I= Techniques of CL repair using resorbable sutures, nonabsorbable sutures, medical adhesives, or any combination of these,<br>C= Various sutures used for CL repair<br>O= Wound healing cosmesis and wound healing complications, quality of life, direct and indirect costs to patients and health services, and participant satisfaction.	Inconclusive
123	Orthognathic and osteogenic distraction surgical orthodontics C11	Valls-Ontanon et al	143	2021	P= CLP patients<br>I= Le Fort I osteotomy<br>C= Different surgical protocols<br>O= Stability, recurrence or surgical complications;	Partially conclusive
Sub-domain I - Oral health aspects including dental caries and other dental problems D6						
Nos.	*Subdomain II and code*	*Author*	*Reference number*	*Year*	*Aim and objectives in PICO: Population (P), Intervention (I), Comparison (C), Outcome (O)*	*Grade of Conclusion*
124	N/A	Worth et al	144	2017	P= Children born with an orofacial cleft<br>I= Prevalence of dental caries	Conclusive

Annexure 5: Detailed characteristics of the included studies including Domain, Subdomain I and code, Subdomain II and code (refer to Figure 1 and Table 1 for subdomains and codes), author, reference number (as numbered in the reference list of paper) year, aims and objectives in P (Population) I/E (Intervention/exposure) C (Comparison) O (Outcome), and grade of conclusion. Color codes have been done for different Domains and subdomains (I and II) are as presented in Table 1. Abbreviations: CLP (cleft lip and/ or cleft palate), CL (cleft lip), CP (cleft palate), CLA (Cleft lip alveolus), UCLP (Unilateral cleft lip and palate), BCLP (bilateral cleft lip and palate). Refer to main text of the systematic review using reference number (3rd column).

					C= Individuals without cleft<br>O= Risk of dental caries	
125	Conventional orthodontics C6	Vale et al	145	2021	P= CLP patients that underwent orthodontic treatment<br>I= Methods for assessment of S. mutans count<br>C= The control group consisted of participants that underwent orthodontic treatment without CLP<br>O= Levels of S. mutans and Lactobacillus higher to 105 CFU/ml	Partially conclusive
**Domain. E. PSYCHOSOCIAL ASPECTS**						
Sub-domain I - Psychosocial aspects, perceptions and quality of life of family members of the patients with CLP, lay people and healthcare professionals E1						
Nos .	*Subdomain II and code*	*Author*	*Reference number*	*Year*	*Aim and objectives in PICO : Population (P), Intervention (I), Comparision (C), Outcome (O)*	*Grade of Conclusion*
126	N/A	Zhu et al	146	2016	P= CLP patients' visualization by lay people<br>I= Full facial images<br>C= CLP visualization by professionals<br>O= Difference in critical appraisal in facial appearance of CLP patients	Inconclusive
127	Presurgical Infant Orthopaedics and Naso-Alveolar Moulding C3	El-Ghafour et al	147	2020	P= Parents with unilateral CLP infants<br>I= AM and NAM appliances<br>C= Untreated control (only surgical lip repair)<br>O= Parents' satisfaction	Inconclusive
128	Feeding and nutrition in patients C2	Srivastav et al	148	2021	P= Parents of children with CLP<br>I= Questionnaires for assessment of parental response to feeding<br>C= Comparator Not applicable<br>O= Outcome Parental response to feeding behaviour	Partially conclusive
Sub-domain I - Psychosocial aspects, perceptions and quality of life of patients with CLP E2						
Nos .	*Subdomain II and code*	*Author*	*Reference number*	*Year*	*Population (P), Intervention (I), Comparision (C), Outcome (O)*	*Conclusion derived from the study*
129	N/A	Antonarakis et al	149	2013	P= Non-syndromic CLP patients<br>I= questionnaire<br>C= healthy non-cleft individuals<br>O= OHRQoL	Conclusive
130	N/A	Herkrath et al	150	2015	P= Individuals with Non-syndromic Orofacial Clefts<br>I= Questionnaire<br>C= children without cleft<br>O= Health-Related and Oral Health–Related Quality of Life	Conclusive

Annexure 5: Detailed characteristics of the included studies including Domain, Subdomain I and code, Subdomain II and code (refer to Figure 1 and Table 1 for subdomains and codes), author, reference number (as numbered in the reference list of paper) year, aims and objectives in P (Population) I/E (Intervention/exposure) C (Comparison) O (Outcome), and grade of conclusion. Color codes have been done for different Domains and subdomains (I and II) are as presented in Table 1. Abbreviations: CLP (cleft lip and/ or cleft palate), CL (cleft lip), CP (cleft palate), CLA (Cleft lip alveolus), UCLP (Unilateral cleft lip and palate), BCLP (bilateral cleft lip and palate). Refer to main text of the systematic review using reference number (3rd column).

131	N/A	Chung et al	151	2019	P= CLP paediatric patients in low-resource areas<br>E= social stigma<br>C= children without CLP/ children with CLP in high income countries<br>O= impact of social stigma and psychosocial aspects	Conclusive
132	Other surgical aspects of cleft lip and palate including revisions, scar management, post-operative pain and anaesthesia C12	Acum et al	152	2020	P=CLP patients at the end of the cleft surgery pathway<br>I= Questionnaire assessing patients related PROs,<br>C= Comparator group<br>O= Psychosocial patient-reported outcomes	Inconclusive
133	Anatomical and functional characteristics A6	Glinianaia et al	153	2021	P= Children with congenital anomalies<br>I= (School test/ Questionnaire-based) or data on special education needs (SEN)<br>C=controls<br>O= academic outcomes and special education needs (SEN)	Conclusive
134	Conventional orthodontics C6	Nascimento et al	154	2021	P= Patients with CL and/or palate of all ages undergoing orthodontic treatment. Syndromic patients or patients with neurological disorders were excluded<br>I= Any form of orthodontic treatment, interceptive and/or corrective, involving all age groups<br>C= Different clinical and demographic factors<br>O= Measurement of OHRQoL through questionnaires	Conclusive
Sub-domain I - Burden of care E3						
Nos.	*Subdomain II and code*	*Author*	*Reference number*	*Year*	*Population (P), Intervention (I), Comparision (C), Outcome (O)*	*Conclusion derived from the study*
135	Presurgical Infant Orthopaedics and Naso-Alveolar Moulding C3	Alfonso et al	155	2020	P= Care givers of CLP patients who underwent Naso-alveolar molding<br>I= Various questionnaires<br>C= None<br>O= Burden of Care of Naso-alveolar molding (physical, psychosocial, or financial burden)	Partially conclusive
Sub-domain I - Health economics E4						
Nos.	*Subdomain II and code*	*Author*		*Year*	*Population (P), Intervention (I), Comparision (C), Outcome (O)*	*Conclusion derived from the study*
136	Burden of care E3	Saxton et al	156	2016	P= Children in Low- and Middle-Income Countries<br>I= Surgical Care<br>C= None<br>O= Economic Analysis/ economic impact	Partially conclusive
**Domain. F. PREVENTIVE**						
Sub-domain I - Risk assessment and prediction F2						

Annexure 5: Detailed characteristics of the included studies including Domain, Subdomain I and code, Subdomain II and code (refer to Figure 1 and Table 1 for subdomains and codes), author, reference number (as numbered in the reference list of paper) year, aims and objectives in P (Population) I/E (Intervention/exposure) C (Comparison) O (Outcome), and grade of conclusion. Color codes have been done for different Domains and subdomains (I and II) are as presented in Table 1. Abbreviations: CLP (cleft lip and/ or cleft palate), CL (cleft lip), CP (cleft palate), CLA (Cleft lip alveolus), UCLP (Unilateral cleft lip and palate), BCLP (bilateral cleft lip and palate). Refer to main text of the systematic review using reference number (3rd column).

Nos.	*Subdomain II and code*	*Author*	*Reference number*	*Year*	*Population (P), Intervention (I), Comparision (C), Outcome (O)*	*Conclusion derived from the study*
137	Genetics A4	Zhao et al	157	2016	P= Non-syndromic cleft of the lip and/or palate<br>E= MTHFD1 polymorphism rs 2236225<br>C= None<br>O= Association between the NSCL/P and MTHFD1 polymorphism rs2236225,	Partially conclusive
138	Genetics A4	Wang et al	158	2018	P= Non syndromic CLP diagnosed clinically<br>E= WNT3 polymorphisms<br>C= Healthy individuals<br>O= NSCL/P susceptibility	Partially conclusive
139	Genetics A4	Shu et al	159	2019	P= Non-syndromic CL with or without palate<br>E= Methylenetetrahydrofolate reductase polymorphisms<br>C= None<br>O= association	Partially conclusive
140	Genetics A4	Zhou et al	160	2020	P= Pregnant women<br>E= Folate supplement use, dietary folate intake, folic acid fortification, biomarkers of folate status, and MTHFR genetic variants<br>C= None<br>O= non-syndromic OFCs	Partially conclusive

Domain. G. RESEARCH METHODS

Sub-domain I - Clinical practice guidelines and Core outcome sets G1

Nos.	*Subdomain II and code*	*Author*	*Reference number*	*Year*	*Population (P), Intervention (I), Comparision (C), Outcome (O)*	*Conclusion derived from the study*
141	Oral health aspects including dental caries and other dental problems D6	Saikia et al	161	2021	P= Children With CLP<br>I= Clinical Practice Guidelines(CPGs) for Oral Health<br>C= None<br>O= scope, quality, adequacy, and consistency of CPGs	Partially conclusive

Sub-domain I - Methodological aspects G3

Nos.	*Subdomain II and code*	*Author*	*Reference number*	*Year*	*Population (P), Intervention (I), Comparision (C), Outcome (O)*	*Conclusion derived from the study*
142	N/A	Hardwicke et al	162	2017	P= Patients with CL and/or cleft palate<br>I= None<br>C= none<br>O= Outcome of RCT, S= Prospective randomized clinical trials (RCTs)	Conclusive

Annexure 5: Detailed characteristics of the included studies including Domain, Subdomain I and code, Subdomain II and code (refer to Figure 1 and Table 1 for subdomains and codes), author, reference number (as numbered in the reference list of paper) year, aims and objectives in P (Population) I/E (Intervention/exposure) C (Comparison) O (Outcome), and grade of conclusion. Color codes have been done for different Domains and subdomains (I and II) are as presented in Table 1. Abbreviations: CLP (cleft lip and/ or cleft palate), CL (cleft lip), CP (cleft palate), CLA (Cleft lip alveolus), UCLP (Unilateral cleft lip and palate), BCLP (bilateral cleft lip and palate). Refer to main text of the systematic review using reference number (3rd column).

**Domain. H. RECENT ADVANCES**						
Sub-domain I - Any recent research area not covered in the above categorization H1						
Nos.	*Subdomain II and code*	*Author*	*Reference number*	*Year*	*Population (P), Intervention (I), Comparision (C), Outcome (O)*	*Conclusion derived from the study*
143	Alveolar bone grafting and graft materials C10	Shanbhag et al	163	2019	P= CLP<br>I= Cell therapy<br>C= Grafting with only biomaterial scaffolds and/or autogenous bone<br>O= Effect of cell therapy in terms of orofacial bone regeneration	Inconclusive
144	Genetics A4	Wei et al	164	2022	P= Non-syndromic CLP patients<br>I= Cell experiment<br>C= Control group<br>O= Association of NSCLP and non-coding RNAs	Conclusive
