# Supplementary material for: Evidence Mapping and Quality Analysis of Systematic Reviews on Various Aspects Related to Cleft Lip and Palate
Source: J Clin Med. 2023 Sep 16;12(18):6002. doi: 10.3390/jcm12186002 (PMC10532364; doi:10.3390/jcm12186002)
Supplement: Supplementary file 1 [file jcm-12-06002-s001.zip › Annexure 7.pdf]

Annexure 7: Table showing details of ongoing studies registered in PROSPERO and their categorization as domains and subdomains of cleft lip and palate. For subdomains (Subdomain I and Subdomain II) and the codes, refer to Table 1 and Figure 1.

**Domain A: EPIDEMIOLOGIC**					
**Subdomain I**			**Incidence And Prevalence, A1**		
**SNo**	**First Author**	**Title**	**Year**	**Subdomain II**	Code
1	Shahd Mustafa CRD42019121276	The Prevalence of Dental Anomalies In Non-Syndromic Individuals With Cleft Lip And/Or Palate: A Systematic Review And Meta-Analysis	2019		
2	Elias Yirdaw CRD42021266844	Prevalence Of Cleft Lip Or/And Cleft Palate And Its Associated Factors: A Systematic Review And Meta-Analysis Of Studies In Africa	2021	Etiopathogenesis	A2
**Subdomain I**			**Etiopathogenesis, A2**		
**SNo**	**First Author**	**Title**	**Year**	**Subdomain II**	Code
3	Gemma Sharp CRD42015026004	Risk Factors for Orofacial Clefts	2015		
4	Puhan He CRD42017074813	The Effect of Folic Acid Supplementation on The Prevention Of Cleft Lip And Palate: A Systematic Review And Meta-Analysis	2017		
5	Estefania Ramírez CRD42018075593	Risk Factors (O, C) Identified in The Presentation Of Non Syndromic Cleft Lip And/Or Cleft Palate(I) In Latin America (P): A Systematic Review	2018		
6	Mengwen Zheng CRD42018111050	RARA Gene Polymorphisms and Nonsyndromic Cleft Lip and Palate Risk: A Systematic Review And Meta-Analysis	2018	Genetics	A4
7	Aida Abou-Zamzam CRD42018104942	Systematic Review and Meta-Analysis of Maternal Folic Acid Supplementation And The Risk Of Orofacial Clefts In Offspring	2018		
8	Lucimara Teixeira Das Neves CRD42020139014	Environmental Risk Factors in The Non-Syndromic Cleft Lip And Palate Etiology: A Systematic Review.	2020		
9	Thirona Naicker CRD42020199361	Systematic Review on The Known Variants And Genes Associated With Orofacial Clefts In Africa	2020	Genetics	A4
10	Azeez Alade CRD42021268825	A Systematic Review and Meta-Analysis To Determine The Association Between Periconceptional Use Of Vitamin A And The Risk Of Giving Birth To A Child With Non-Syndromic Orofacial Clefts (Nsofcs)	2021	Embryologic Development	A3
11	Adriana Da Silva CRD42021295575	Association Between Orofacial Clefts and Cancer: A Systematic Review And Meta-Analysis	2021	Systemic Problems	D7
**Subdomain I**			**Anatomical And Functional Characteristics, A6**		
**SNo**	**First Author**	**Title**	**Year**	**Subdomain II**	Code

Annexure 7: Table showing details of ongoing studies registered in PROSPERO and their categorization as domains and subdomains of cleft lip and palate. For subdomains (Subdomain I and Subdomain II) and the codes, refer to Table 1 and Figure 1.

12	Paulo Martins-Filho<br>CRD42015027327	Analysis Of The Pharyngeal Airway Volume In Children And Adolescents With Cleft Lip And Palate Using Cone Beam Computed Tomography: Systematic Review And Meta-Analysis	2015	Advanced Radiography Including 3d Imaging And 3d Printing	B6
13	Victoria Worth<br>CRD42015020403	Oral Health In (Children With) Cleft Lip And Palate: A Systematic Review And Meta-Analysis	2015	Oral Health Aspects Including Dental Caries And Other Dental Problems	D6
14	Emily Tamie Sá Nakamura<br>CRD42015020041	Simonart's Band Influence On Maxilo-Mandibular Relationship Of Complete Cleft Lip And Palate Patients: A Systematic Review	2015		
15	Anne Marie Kuijpers-Jagtman<br>CRD42018088945	Comparison Of Pharyngeal Airway Space Between Cleft Lip And Palate Patients And Non-Cleft Individuals: A Systematic Review	2018		
16	Evelyn Del Rosario Munayco Pantoja<br>CRD42020209876	Enamel Defects In Non-Syndromic Patients With Cleft Lip And Palate: A Systematic Review And Meta-Analysis	2020		
17	Allessandra Fraga Da Re<br>CRD42020181208	Myofunctional Orofacial Evaluation For Cleft Lip And Palate: Systematic Review	2020		
18	Valerie Hereman<br>CRD42016051796	Tooth Impaction In Cleft Patients: A Systematic Review	2020		
19	Ana Carolina Exner Fernandes Barros<br>CRD42021281041	Prevalence Of Obstructive Sleep Apnea In Pediatric Patients With Cleft Lip And Palate: A Systematic Review	2021	Obstructive Sleep Apnoea	D5
20	Simar Sethi<br>CRD42021292425	Is caries prevalence site-specific in cleft lip and palate patients? : A systematic review and meta-analysis	2021	Oral Health Aspects Including Dental Caries And Other Dental Problems	D6
21	Karina Gabryella maringonda Vicente<br>CRD42021273836	Maxillary Sinus Analysis By Cone Beam Computed Tomography In Patients Diagnosed With Unilateral Cleft Palate: A Systematic Review	2021	Advanced Radiography Including 3d Imaging And 3d Printing	B6
22	Eva Cadenas<br>CRD42022297888	Craniofacial Skeletal Relations In Patients With Cleft Lip And Palate Using Multivariation Analysis	2022		
23	Inês Francisco<br>CRD42022287414	Velopharyngeal Insufficiency Treatment In Cleft Palate Patients: Umbrella Review	2022		
**Domain B: DIAGNOSTIC**					
**Subdomain I**			**Prenatal Diagnosis, B3**		
**SNo**	**First Author**	**Title**	**Year**	**Subdomain II**	**Code**
24	Joana Prota<br>CRD42016036400	Diagnostic Yield Of Chromosomal Microarray For Etiologic Diagnosis Of Typical Orofacial Clefts: A Systematic Review And Meta-Analysis	2016	Genetics	A4
**Subdomain I**			**Clinical Diagnostic Methods, B4**		

Annexure 7: Table showing details of ongoing studies registered in PROSPERO and their categorization as domains and subdomains of cleft lip and palate. For subdomains (Subdomain I and Subdomain II) and the codes, refer to Table 1 and Figure 1.

SNo	First Author	Title	Year	Subdomain II	Code
25	Elena González Cano CRD42021232044	Facial Asymmetry Assessment In Cleft And Non-Cleft Patients	2021		
26	Claudia Acosta Lenis CRD42021291821	Maxillary Distraction Osteogenesis And/Or Le Fort I In Cleft Lip And Palate Patients. Is The 10 Mm Discrepancy A Baseline To Decide?: A Systematic Review	2021	Orthognathic And Osteogenic Distraction Surgical Orthodontics	C11
**Subdomain I**			**Conventional Radiography and 2D Imaging, B5**		
**SNo**	**First Author**	**Title**	**Year**	**Subdomain II**	**Code**
27	Hayley Llandro CRD42015026476	A Systematic Review To Investigate The Factors That Contribute To The Radiographic Success Of Alveolar Bone Grafting In Cleft Lip And Palate Patients	2015	Alveolar Bone Grafting And Graft Materials	C10
28	Karthik Sennimalai CRD42021261941	Can Cephalometric Parameters Predict The Future Need For Orthognathic Surgery In Non-Syndromic Cleft Patients? A Systematic Review And Meta-Analysis	2021	Orthognathic And Osteogenic Distraction Surgical Orthodontics	C11
29	Nameksh Raj Bhupali CRD42021257549	Radiographic Assessment Of Pharyngeal Airway In Non-Syndromic Patients With Repaired Cleft Lip And Palate: A Systematic Review	2021	Anatomical And Functional Characteristics	A6
30	Katarzyna Lewusz CRD42014013079	Two-Dimensional And Three-Dimensional Imaging In Cleft Palate Patients: Systematic Review	2014	Advanced Radiography Including 3d Imaging And 3d Printing	B6
**Subdomain I**			**Advanced Radiography Including 3D Imaging and 3D Printing, B6**		
**SNo**	**First Author**	**Title**	**Year**	**Subdomain II**	Code
31	Ana Maria Ceron-Zapata CRD42017069500	Comparison Of Three-Dimensional Changes Of Nasal Soft Tissue And The Maxillary Arch In Patients With Bilateral Cleft Lip And Palate Treated With Presurgical Orthopaedic Therapy. Systematic Review	2017	Presurgical Infant Orthopaedics And Naso-Alveolar Moulding	C3
32	Victor Fabrizio Cabrerapazmino CRD42021262591	Stereophotogrammetry 3d, Direct Measurements, 2d, Calipter And Plaster Models In Patients With Cleft Lip And Palate: Systematic Review Of Comparative Studies	2021	Photography And Records	B7
33	Anna Pelc CRD42020175924	Three Dimensional Methods Used To Asses Soft Tissue Symmetry In Patients With Ucl/P- Systematic Literature Review.	2020		
**Subdomain I**			**Photography And Records, B7**		
**SNo**	**First Author**	**Title**	**Year**	**Subdomain II**	**Code**
34	Jyotsna Unnikrishnan CRD42022298846	Efficacy Of Digital Intraoral Scanning And Digital Models In Cleft Lip And Palate Care	2022		
**Domain C: THERAPEUTIC**					
**Subdomain I**			**Feeding And Nutrition In Patients, C2**		

Annexure 7: Table showing details of ongoing studies registered in PROSPERO and their categorization as domains and subdomains of cleft lip and palate. For subdomains (Subdomain I and Subdomain II) and the codes, refer to Table 1 and Figure 1.

SNo	First Author	Title	Year	Subdomain II	Code
35	Manjubala Dash CRD42020208437	Feeding Interventions Among Cleft Lip/ Palate Infants- A Systematic Review And Meta Synthesis Of Qualitative, Quantitative And Mixed Methods Studies	2020		
**Subdomain I**			**Presurgical Infant Orthopaedics And Naso-Alveolar Moulding, C3**		
**SNo**	**First Author**	**Title**	**Year**	**Subdomain II**	**Code**
36	Himanshi Aggarwal CRD42020213403	Presurgical Naso-Alveolar Molding Device Fabricated Using Cadcam Versus Conventional Technique For Cleft Lip And Palate Patients: A Systematic Review	2020	Advanced Radiography Including 3d Imaging And 3d Printing	B6
37	Julio Villanueva CRD42020147963	Effectiveness Of Pre-Surgical Orthopedics In The Treatment Of Patients With Unilateral Cleft Lip And Palate: Systematic Review And Meta-Analysis	2020	Effects/Complications Of PSIO And PNAM	D2
38	Prabhat Kumar Chaudhari CRD42020186452	Efficacy Of Digital Nasoalveloar Molding To Reduce The Severity Of The Malformation In Individuals With Cleft Lip And/Or Palate: A Systematic Review	2020	Effects/Complications Of PSIO And PNAM	D2
39	Rathindra Nath Bera CRD42021271267	Presurgical Infant Orthopedics Prior To Definitive Treatment In Patients With Cleft Lip And Palate: A Meta Analysis Of Facial Esthetics, Growth, Fistula Rates And Dentoalveolar Arch Forms	2021	Effects/Complications Of PSIO And PNAM	D2
40	Ana Rabal Solans RD42022303417	Evaluation Of Presurgical Orthopedics In The Cleft Lip And Palate	2022		
**Subdomain I**			**Early Orthodontics, C4**		
**SNo**	**First Author**	**Title**	**Year**	**Subdomain II**	**Code**
41	Hamza Siddiqui CRD42021229018	Efficacy Of Bone-Anchored Maxillary Protraction Over Face-Mask In Patients With A Non-Syndromic Unilateral Cleft Lip And Plate- A Systematic Review	2021		
42	Badri Thiruvenkatachari CRD42022248503	Effectiveness Of Prealveolar Bone Graft Orthodontics For Patients With Non-Syndromic Unilateral Cleft Palate: A Systematic Review And Meta-Analysis	2022		
43	Marina Tavares Costa Nobrega CRD42021240533	Effects Of Maxillary Protraction On The Upper Airway Dimensions Of Growing Individuals With Cleft Lip And Palate: A Systematic Review	2021	Anatomical And Functional Characteristics	A6
**Subdomain I**			**Orthodontic And Surgical Perspectives Of Secondary Alveolar Bone Grafting, C5**		
**SNo**	**First Author**	**Title**	**Year**	**Subdomain II**	**Code**
44	Chenzhou Wu CRD42014009942	What Are The Optimal Choices For Clinicians When Conducting Secondary Bone Grafting For Patients With Alveolar Cleft Defects? A Systematic Review	2014		
45	Mohamed Abd El-Ghafour CRD42021261683	Timing Of Orthodontic Treatment And The Success Of Secondary Alveolar Bone Grafting In Patients With Cleft Lip And Palate	2021		

Annexure 7: Table showing details of ongoing studies registered in PROSPERO and their categorization as domains and subdomains of cleft lip and palate. For subdomains (Subdomain I and Subdomain II) and the codes, refer to Table 1 and Figure 1.

**Subdomain I**			**Conventional Orthodontics, C6**		
**SNo**	**First Author**	**Title**	**Year**	**Subdomain II**	**Code**
46	Lucas Cardinal CRD42017075334	Slow And Rapid Maxillary Expansion On Cleft Lip And Palate Subjects: A Systematic Review	2017		
47	Flavia Preda CRD42021239676	Cbct Assessment Of Rapid Palatal Expansion In Patients With Unilateral Cleft - A Systematic Review	2021	Advanced Radiography Including 3d Imaging And 3d Printing	B6
48	Vaibhav Gandhi CRD42021247529	Skeletal And Dental Effects Of The Bone-Anchored Maxillary Protraction Therapy In Cleft Lip And Palate Patients- A Systematic Review And Meta-Analysis	2021		
49	Jonathan Luyten CRD42022302844	Rapid Maxillary Expansion Versus Slow Maxillary Expansion In Cleft Lip And Palate Patients: A Systematic Review And Meta Analysis	2022		
**Subdomain I**			**Cleft Lip Repair, C8**		
**SNo**	**First Author**	**Title**	**Year**	**Subdomain II**	**Code**
50	Anuj Jain	Comparison Of Octyl – 2 Cyanoacrylate Glue With Other Closure Materials In Terms Of Esthetics And Clinical Outcomes After Cleft Lip Repair Amongst Overall Healthy Patients-A Systematic Review And Meta-Analysis	2021	Outcome Assessment	C15
**Subdomain I**			**Cleft Alveolus, Submucous Cleft And All Types Of Cleft Palate Closure/Repair, C9**		
**SNo**	**First Author**	**Title**	**Year**	**Subdomain II**	**Code**
51	Matthew Fell CRD42021296991	Surgical Approaches To Primarily Reconstruct A Wide Cleft Palate: A Systematic Review	2021		
52	Griffin Bins CRD42021282889	Effect Of Septoplasty In Cleft Repair	2021		
53	Fiachra Sheil CRD42021238882	Efficacy Of Interventions In Preventing Post-Operative Bleeding In Cleft Palate Repair: A Systematic Review	2021		
54	Eric Nagengast CRD42020193982	Timing Of Primary Cleft Palate Surgery And Its Association With Postoperative Velopharyngeal Insufficiency: A Systematic Review	2020		
**Subdomain I**			**Alveolar Bone Grafting And Graft Materials, C10**		
**SNo**	**First Author**	**Title**	**Year**	**Subdomain II**	**Code**
55	Weiyi Pan CRD42014009253	Minimally Invasive Versus Traditional Open Harvesting Techniques For Iliac Bone Graft In Secondary Alveolar Cleft Patients: A Systematic Review	2014		
56	Francisco Filho CRD42016032665	Use Of Rhbmp-2 As An Alternative To Autogenous Bone Grafts For The Treatment Of Alveolar Cleft: A Systematic Review	2016		
57	Leandro Chambrone CRD42016039040	What Is The Best Substitute For Autologous Bone Graft For The Regeneration Of Alveolar Clefts In Humans? A Systematic Review	2016		

Annexure 7: Table showing details of ongoing studies registered in PROSPERO and their categorization as domains and subdomains of cleft lip and palate. For subdomains (Subdomain I and Subdomain II) and the codes, refer to Table 1 and Figure 1.

58	Prakriti Garkhail CRD42020171377	Intra- And Inter-Rater Analysis Of Linear Outcome Measurement In Secondary Alveolar Bone Graft Surgery Of Cleft Lip And Palate: A Systematic Review	2020	Outcome Assessment	C15
59	Manjula Herath CRD42020197451	Grafting Materials For Alveolar Cleft Reconstruction: A Systematic Review And Meta-Analysis	2020		
60	Alice Nobelika.A CRD42021251327	Role Of Regenerative Scaffolds As A Substitute For Autogenous Bone Graft In Cleft Patients-A Systematic Review	2021		
61	Ting Li CRD42022312588	Outcomes Of Platelet-Rich Fibrin And Iliac Cancellous Bone Transplantation On Alveolar Cleft Bone Grafting: A Meta-Analysis	2022		
**Subdomain I**			**Orthognathic And Osteogenic Distraction Surgical Orthodontics, C11**		
**SNo**	**First Author**	**Title**	**Year**	**Subdomain II**	**Code**
62	Hussam Okba CRD42016050108	Maxillary Skeletal Stability In Non-Growing Cleft Lip And Palate Patients With Maxillary Hypoplasia Using Conventional Orthognathic Surgery Versus Internal Distraction Osteogenesis: Systematic Review And Meta-Analysis	2016	Outcome Assessment	C15
63	Ildeu Andrade Jr CRD42018117952	Maxillary Protraction In Cleft Lip And Palate Patients: A Systematic Review.	2018	Conventional Orthodontics	C6
64	Jingadekrishnoji Rao Dayashankara Rao CRD42020204710	A Comparative Evaluation Of Anterior Maxillary Distraction And Le-Fort 1 Osteotomy In Cleft Maxillary Hypoplasia For Speech And Relapse. -A Systematic Review	2020	Outcome Assessment	C15
65	Alia Eid CRD42022301302	Maxillary Distraction Osteogenesis Versus Conventional Orthognathic Surgery In Cleft Lip And Palate Patients: Systematic Review	2022		
**Subdomain I**			**Other Surgical Aspects Of Cleft Lip And Palate Including Revisions, Scar Management, Post-Operative Pain And Anaesthesia,C12**		
**SNo**	**First Author**	**Title**	**Year**	**Subdomain II**	**Code**
66	Shengkai Chen CRD42018090279	The Effects Of Primary Rhinoplasty In Cleft Lip +/- Palate Children	2018		
67	Alexander Morzycki CRD42018115831	Perioperative Analgesia For Children Undergoing Primary Cleft Lip And Palate Repair: A Systematic Review And Meta-Analysis	2018		
68	Christine Ramzy CRD42021129413	Management Of Post-Surgical Scarring In Cleft Lip And/Or Palate After Primary Repair: A Systematic Review	2021		
**Subdomain I**			**Management Of Speech and Hearing Problems, C14**		
**SNo**	**First Author**	**Title**	**Year**	**Subdomain II**	**Code**
69	Yan Yi Lam CRD42019116016	The Role Of Speech Therapy For Improving The Speech Of Children With Cleft Lip And/Or Cleft Palate: A Systematic Review And Meta-Analysis	2019		

Annexure 7: Table showing details of ongoing studies registered in PROSPERO and their categorization as domains and subdomains of cleft lip and palate. For subdomains (Subdomain I and Subdomain II) and the codes, refer to Table 1 and Figure 1.

70	Gabriela Ribeiro Schilling CRD42021209088	Therapies For Velopharyngeal Dysfunction In Children With Cleft Lip And Palate: Systematic Literature Review	2021		
71	Miriam Platte CRD42021244429	Phonological And Articulatory Speech Therapy Interventions For Children With Cleft Palate: A Systematic Review	2021		
**Subdomain I**			**Outcome Assessment, C15**		
**SNo**	**First Author**	**Title**	**Year**	**Subdomain II**	**Code**
72	Philippa Drury CRD42020199893	A Systematic Review Of The Quality Of Photographic Rating Systems For The Assessment Of Aesthetic Outcome Following Cleft Lip Surgery	2020	Photography And Records	B7
73	Shreya S CRD42021261405	Comparison Of Predicted Outcomes In Presurgical Cleft Patients With And Without Presurgical Nasoalveloar Moulding	2021	Presurgical Infant Orthopaedics And Naso-Alveolar Moulding	C3
74	Percy Rossell-Perry CRD42021240526	Does The Use Of Postoperative Nasal Conformers Improve Nasal Outcomes In Cleft Lip And Palate Patients?	2021	Presurgical Infant Orthopaedics And Naso-Alveolar Moulding	C3
75	Jianan Yan CRD42022290070	Comparison Between Aesthetic Assessments Given By The Cleft Patients And The Professionals: A Systematic Review	2022	Psychosocial Aspects, Perceptions And Quality Of Life Of Patients With Clp	E2
**Domain D: PROGNOSTIC**					
**Subdomain I**			**Effects/Complications Of Untreated CLP, D1**		
**SNo**	**First Author**	**Title**	**Year**	**Subdomain II**	**Code**
76	Tarek Balam CRD42020148494	Effect Of The Cleft Lip And Palate On Maxillofacial Growth: A Systematic Review And Network Meta-Analysis	2020	Anatomical And Functional Characteristics	A6
**Subdomain I**			**Effects/Complications Of PSIO And PNAM, D2**		
**SNo**	**First Author**	**Title**	**Year**	**Subdomain II**	**Code**
77	Sávio Gontijo CRD42018107909	Nasal Tissues Changes In Individuals With Unilateral Cleft Lip Or Unilateral Cleft Lip And Palate Treated With Presurgical Orthopedic Therapy: A Systematic Review.	2018		
78	Rajesh Kumar CRD42020175292	Does Presurgical Infant Orthopedics Have Any Long-Term Effect On Maxillary Growth In Patients With Cleft Lip And Palate - A Systematic Review	2020		
79	Sanjeev Datana CRD42020195879	Long Term Outcome Of Presurgical Infant Orthopaedics In Patients With Cleft Lip And Palate—A Systematic Review	2020	Outcome Assessment	C15
80	Li Chunrong CRD42020142026	Long-Term Effects Of Presurgical Nasoalveolarmolding On Unilateral Complete Cleft Lip And Palate: Systematic Review And Meta-Analysis.	2020	Outcome Assessment	C15
81	Ard likitkulthanaporn CRD42021283847	Long-Term Therapeutic Effects Of Pre-Surgical Nasoalveolar Molding In Patients With Cleft Lip And Palate: A Systematic Review And Meta-Analysis.	2021	Outcome Assessment	C15

Annexure 7: Table showing details of ongoing studies registered in PROSPERO and their categorization as domains and subdomains of cleft lip and palate. For subdomains (Subdomain I and Subdomain II) and the codes, refer to Table 1 and Figure 1.

82	Abhijeet Tribhuvan CRD42021234572	Nasal Changes Seen After Presurgical Nasoalveolar Moulding In Unilateral Cleft Lip And Palate Patients: A Systematic Review	2021		
83	Maria Dolores Rocha-Eiroa CRD42022283494	Long-Term Effects Of Presurgical Pediatric Orthopedics On Maxillofacial Growth And Dental Arch Dimensions In Patients With Non-Syndromic Cleft Lip And/Or Palate.	2022	Outcome Assessment	C15
**Subdomain I**			**Effects/Complications Of Orthodontic Treatment, D3**		
**SNo**	**First Author**	**Title**	**Year**	**Subdomain II**	Code
84	Ana Paula Herkrath CRD42018085181	Effect Of Previous Orthodontic Treatment On The Prognosis Of Alveolar Bone Graft In Individuals With Cleft Lip And Palate: A Systematic Review	2018	Alveolar Bone Grafting And Graft Materials	C10
85	Jessica Arieta CRD42020203417	Dental And Skeletal Effects Of Non-Surgical Maxillary Expansion In Patients With Cleft Lip And Palate: A Systematic Review.	2020	Conventional Orthodontics	C6
86	Rodrigo Almeida Nunes Teixeira CRD42020206800	Dentoskeletal Effects Of Rapid Maxillary Expansion In Cleft Patients: A Systematic Review.	2020	Conventional Orthodontics	C6
87	Renata Bastos CRD42021290378	Influence Of The Orthodontic Retainers And Oral Rehabilitation On The Stability Of The Maxillary Dental Arch In Patients With Unilateral Cleft Lip And Palate: A Systematic Review	2020	Conventional Orthodontics	C6
88	Xinqi Huang CRD42021242177	Effect Of Bone-Anchored Maxillary Protraction On Temporomandibular Joint In Patients With Unilateral Cleft Lip And Palate Compared To Tooth-Anchored Maxillary Protraction?	2021	Conventional Orthodontics	C6
**Subdomain I**			**Effects/Complications Of Surgical Treatment And/Or Alveolar Bone Grafting, D4**		
**SNo**	**First Author**	**Title**	**Year**	**Subdomain II**	**Code**
89	Lorena Fernandes Ciaccia CRD42018111851	Stability Of Le Fort I Osteotomy In Cleft Lip And Palate Patients: A Systematic Review	2018	Orthognathic And Osteogenic Distraction Surgical Orthodontics	C11
90	Lurian Minatel CRD42018095714	Impact Of Surgical Protocol For Repair Of Hard Palate On Craniofacial Growth In Patients With Cleft Lip And Palate: Systematic Review And Meta-Analysis	2018		
91	Pedro Henrique Da Hora Sales CRD42020195927	Complications In Orthognathic Surgery In Cleft Patients. A Systematic Review.	2020		
92	Sirius Dan Inaoka CRD42020154944	Does Using Prf Associated With Autogenous Bone Graft In Cleft Patient Improve The Volume And The Density Of The Grafted Bone? A Systematic Review And Meta-Analysis	2020	Alveolar Bone Grafting And Graft Materials	C10
93	Rathindra Nath Bera CRD42020223310	Effect Of Single Stage Versus Two Stage (Soft Palate Followed By Hard Palate Repair) Versus Staged Hard Palate Repair On Facial Growth, Speech, Otologic Function And Occlusion Relationship In Patients With Unilateral And Bilateral Cleft Lip And Palate.	2020	Cleft Alveolus, Submucous Cleft And All Types Of Cleft Palate Closure/Repair	C9

Annexure 7: Table showing details of ongoing studies registered in PROSPERO and their categorization as domains and subdomains of cleft lip and palate. For subdomains (Subdomain I and Subdomain II) and the codes, refer to Table 1 and Figure 1.

94	Tarnjit Sangha CRD42020209080	Network Meta-Analysis: Factors That Predict Success In Bone Grafting In Cleft Lip And Palate	2020		
95	Karina Freitas CRD42020205656	Relapse After Non-Surgical Orthodontic Treatment In Patients With Unilateral Cleft Lip And Palate: A Systematic Review.	2020	Outcome Assessment	C15
96	Lingzhi xie CRD42021258202	Effect Of Age At Premaxillary Osteotomy On Facial Growth In Bilateral Cleft Lip/Palate Patients: A Systematic Review	2021	Anatomical And Functional Characteristics	A4
97	Joanna Janiszwewska – Olszowska CRD42021268957	Influence Of Palatal Surgery On Craniofacial Morphology In Patients With Cleft Palate Without Cleft Lip	2021		
98	Alexander Schulze Wenning CRD42021289846	One-Stage Or Two-Stage Palatoplasty, Comparing Maxillofacial Growth And Speech Outcome In Patients With Uni- And Bilateral Cleft Lip And Palate	2021	Cleft Alveolus, Submucous Cleft And All Types Of Cleft Palate Closure/Repair	C9
99	Cui Cui CRD42021234921	Survival Rate Of Dental Implant In Patients With Cleft Lip And Palate: A Systematic Review And Meta-Analysis	2021	Comprehensive Interdisciplinary Care	C13
**Subdomain I**			**Obstructive Sleep Apnoea, D5**		
**SNo**	**First Author**	**Title**	**Year**	**Subdomain II**	**Code**
100	Milton Chin CRD42020165781	Obstructive Sleep Apnea Following Velopharyngeal Surgery In Children With Non-Syndromic Repaired Cleft Palate: A Systematic Review	2020	Cleft Alveolus, Submucous Cleft And All Types Of Cleft Palate Closure/Repair	C9
**Subdomain I**			**Oral Health Aspects Including Dental Caries And Other Dental Problems, D6**		
**SNo**	**First Author**	**Title**	**Year**	**Subdomain II**	**Code**
101	Vaibhav Sahni CRD42020210178	Does The Presence Of Clefts Alter The Periodontal Status Of Patients?	2020		
**Domain E: PSYCHOSOCIAL ASPECTS, PERCEPTIONS AND QUALITY OF LIFE**					
**Subdomain I**			**Psychosocial Aspects, Perceptions And Quality Of Life Of Family Members Of The Patients With Clp, Lay People And Healthcare Professionals, E1**		
**SNo**	**First Author**	**Title**	**Year**	**Subdomain II**	**Code**
102	Kan Man Carmen Li CRD42021291478	Barriers And Facilitators Of Breastfeeding Amongst Parents Of Infants With Cleft Lip And/Or Palate: A Mixed Methods Systematic Review	2021		
103	Tatiane Cardoso CRD42021270421	Breastfeeding In Children With Cleft Lip And Palate: Breastfeeding Practices And Difficulties	2021		

Annexure 7: Table showing details of ongoing studies registered in PROSPERO and their categorization as domains and subdomains of cleft lip and palate. For subdomains (Subdomain I and Subdomain II) and the codes, refer to Table 1 and Figure 1.

104	Craig Griffiths CRD42017072883	Caregiver-Infant Interactions And Attachment In Children With Orofacial Clefts: A Systematic Review Of The Literature	2017		
105	Sukeshana Srivastav CRD42022306107	Global Status And Trends Of Knowledge, Attitude, Awareness And Practice For Management Of Cleft Lip And Palate Patients Among The Orthodontists	2022		
**Subdomain I**			**Psychosocial Aspects, Perceptions And Quality Of Life Of Patients With CLP, E2**		
**SNo**	**First Author**	**Title**	**Year**	**Subdomain II**	**Code**
106	Ashleigh Nocher CRD42012003097	What Is The Psycho-Social Impact Of Dental And Orthodontic Related Treatment In Patients With Cleft Lip And Palate?	2012		
107	Brent Collett CRD42017079689	Academic Outcomes For Children With Orofacial Clefts: A Systematic Review Of The Literature	2017		
108	Dan Liu CRD42018095134	The Effects Of Cleft Lip And/Or Palate On Patients' Self-Esteem And Psychological Interventions To Improve Their Self-Esteem: A Systematic Review	2018		
109	Kinga Amalia Sandor-Bajusz	The Presence Of Mental Disorders In Individuals With Isolated Cleft Lip And Palate	2020		
110	Camila De Castro Corrêa CRD42021231494	Prevalence Of Social Stigma And Bullying In Individuals With Cleft Lip And Palate: Systematic Review	2021		
**Subdomain I**			**Burden Of Care, E3**		
**SNo**	**First Author**	**Title**	**Year**	**Subdomain II**	**Code**
111	Nuria Bengoechea De La Fuente CRD42022295182	Unmet Needs And Burden In Caregivers Of Patients With Cleft Lip And Palate	2022	Psychosocial Aspects, Perceptions And Quality Of Life Of Family Members Of The Patients With CLP, Lay People And Healthcare Professionals	E1
**Domain G: RESEARCH METHODS**					
**Subdomain I**			**Clinical Practice Guidelines And Core Outcome Sets, G1**		
**Sno**	**First Author**	**Title**	**Year**	**Subdomain II**	**Code**
112	Asha Chowdhar CRD42021224955	Protocols Of Cleft Lip And Palate Treatment And Management Strategies: A Systematic Review.	2021	Treatment Planning And Prediction	C1
113	Peter Anto Johnson CRD42021258625	Clinical Guidelines For Cleft Lip And Palate Care: A Systematic Review	2021		
114	Mohammad Zeinalddin CRD42021280979	Protocols In Presurgical Infant Orthopedic Treatment – An Evidence Based Review	2021	Presurgical Infant Orthopaedics And Naso-Alveolar Moulding	C3

Annexure 7: Table showing details of ongoing studies registered in PROSPERO and their categorization as domains and subdomains of cleft lip and palate. For subdomains (Subdomain I and Subdomain II) and the codes, refer to Table 1 and Figure 1.

Domain H: RECENT ADVANCES					
**Subdomain I**			**Any Recent Research Area Not Covered In The Above Categorization, H1**		
**SNo**	**First Author**	**Title**	**Year**	**Subdomain II**	**Code**
115	Alexandre Borba CRD42017070818	The Use Of Platelet Concentrates (Platelet-Rich Plasma (PRP) And Platelet-Rich Fibrin (PRF)) For The Treatment Of Cleft Lip And/Or Palate Subjects: A Systematic Review	2017		
116	Diandra Sabrina Natsirkalla CRD42021259614	Stem Cell-Based Tissue Engineering For Cleft Defects	2021		
117	Mohame d Zahoor Ulhuqh CRD42021270601	The Clinical Applications Of Artificial Intelligence And Machine Learning In Children With Cleft Lip And Palate. A Systematic Review And Meta-Analysis	2021		
