# Supplementary figures and images for: Evidence Mapping and Quality Analysis of Systematic Reviews on Various Aspects Related to Cleft Lip and Palate
Source: J Clin Med. 2023 Sep 16;12(18):6002. doi: 10.3390/jcm12186002 (PMC10532364; doi:10.3390/jcm12186002)

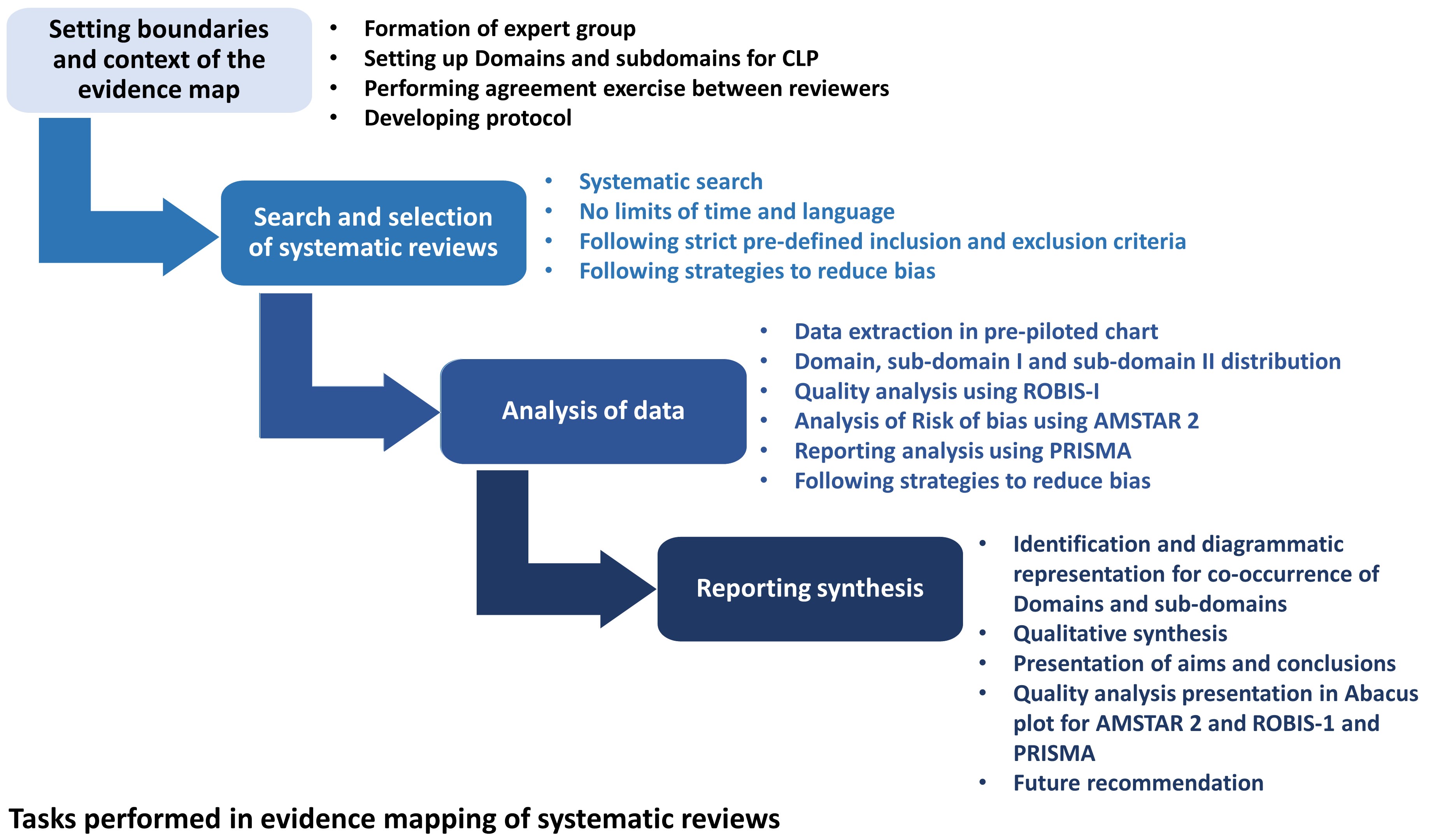

Supplement: Supplementary file 1 [file jcm-12-06002-s001.zip › Annexure 1.jpg]

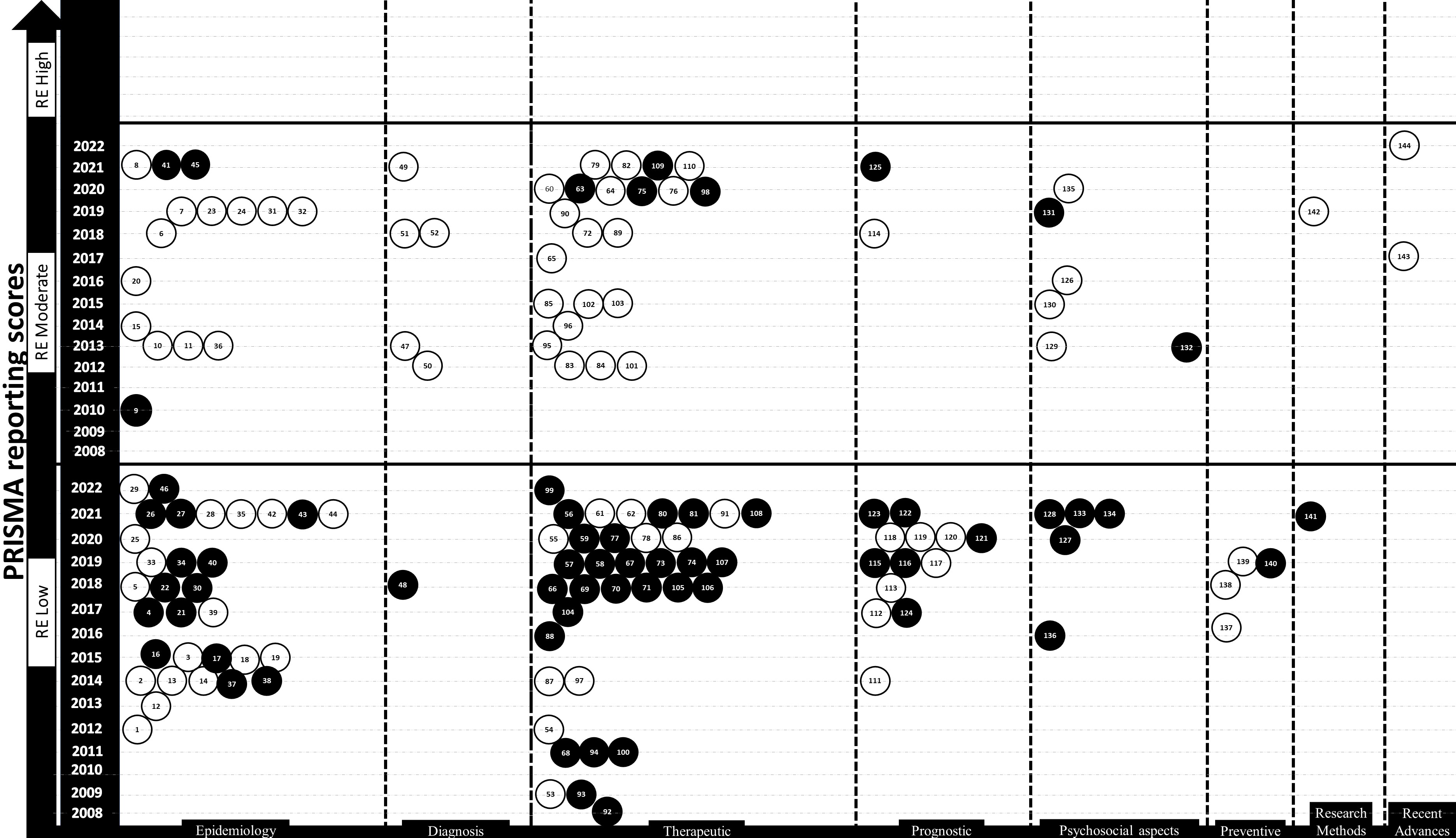

Supplement: Supplementary file 1 [file jcm-12-06002-s001.zip › Annexure 6.jpg]
